# The Impact of a Construction Play on 5- to 6-Year-Old Children’s Reasoning About Stability

**DOI:** 10.3389/fpsyg.2020.01737

**Published:** 2020-07-14

**Authors:** Anke Maria Weber, Timo Reuter, Miriam Leuchter

**Affiliations:** Institute for Children and Youth Education, Educational Sciences, University of Koblenz and Landau, Landau, Germany

**Keywords:** guided play, theory theory, theory-evidence coordination, free play, science learning, intelligence

## Abstract

**Theory:**

Young children have an understanding of basic science concepts such as stability, yet their theoretical assumptions are often not concerned with stability. The literature on theory theory and theory-evidence coordination suggests that children construct intuitive theories about their environment which can be adjusted in the face of counterevidence that cannot be assimilated into the prior theory. With increasing age, children acquire a Center theory when balancing objects and try to balance every object at their middle, succeeding with symmetrical objects. Later, they acquire the basic science concept of stability through learning that the weight distribution of an object is of importance. Thus, they acquire a Mass theory and succeed in balancing asymmetrical objects as well. Fluid and crystallized intelligence might contribute to children’s acquisition of Mass theory. Moreover, their Mass theory might be supported by implementing a playful intervention including (a) material scaffolds and (b) verbal scaffolds.

**Aims:**

We investigated which theories children have about stability and whether these theories can be adjusted to Mass theory by implementing a playful intervention.

**Method:**

A total of 183 5- to 6-year-old children took part in the study with a pre-post-follow-up intervention design. Children’s Mass theory was assessed with an interview in which children explained constructions’ stabilities. The children received a playful intervention with two differing degrees of scaffolding (material scaffolds or material + verbal scaffolds) or no scaffolding.

**Results:**

At first few children used a Mass theory to explain their reasoning. However, after being confronted with counterevidence for the asymmetrical constructions, children changed their explanation and applied a Mass theory. More children in the play group with the highest degree of scaffolding, i.e., material + verbal scaffolds, acquired a Mass theory compared to the other groups. Fluid as well as crystallized intelligence contributed to children’s acquisition of a Mass theory.

**Discussion:**

Counterevidence can support children in their acquisition of a Mass theory. A playful intervention with scaffolding supports children even more.

## Introduction: Science Education

Early knowledge acquisition in the science domain and scientific reasoning lay the foundation for the understanding of complex science concepts, which are relevant throughout the school years and in later life (Eshach and Fried, 2005; Trundle and Saçkes, 2012). Accordingly, studies have demonstrated that science learning and scientific reasoning can be promoted in the early years of childhood (Gelman and Brenneman, 2004; Akerson et al., 2011; Klahr et al., 2011; Cremin et al., 2015).

Children construct intuitive theories to explain what is happening around them, and adjust these theories continuously (Gopnik and Wellman, 2012). These theories encompass science concepts, which might be altered by confronting children with counterevidence (Bonawitz et al., 2012). Promoting young children’s science learning aims at helping them adjust their theories and should consider children’s developmental constraints by considering children’s everyday activities, e.g., their play (Copple and Bredenkamp, 2009). One possibility for such science-related play could be construction play in the form of block play, which is an important leisure activity for young children (Rubin et al., 1978; Pellegrini and Gustafson, 2005; Borriello and Liben, 2018; Verdine et al., 2019). An adult’s guidance might be integrated into children’s play in the form of scaffolding, which might support children’s science learning (cf. van de Pol et al., 2010; Klahr et al., 2011; Mantzicopoulos et al., 2013).

Therefore, children’s science theories might be supported through science-related play that focuses on children’s experiences and encompasses an adult’s scaffolding.

### Theory Theory, Bayesian Inference, and Theory-Evidence Coordination

Fostering scientific reasoning is one goal of science education (Chin, 2007; Klahr et al., 2011). Studies on children’s scientific reasoning rely on literature concerned with theory theory, Bayesian inference and theory-evidence coordination. Theory theory is concerned with the adjustment of children’s intuitive theories when they are confronted with evidence. Bayesian inference focuses on the interaction of intuitive theories with evidence, while theory-evidence coordination investigates the conditions under which children can interpret evidence.

According to theory theory, children construct intuitive theories about their environment, which have similarities with scientific theories (Gopnik and Wellman, 2012). Intuitive and scientific theories share at least five characteristics. (1) They encompass causal representations of the surrounding world, (2) may be hierarchically organized, (3) provide possible explanations for regularities, (4) allow predictions of regularities, and (5) can be adjusted in the face of counterevidence. In this process, not only the explanations for certain subordinate relations but also the general assumptions about regularities can change (Bonawitz et al., 2012; Gopnik and Wellman, 2012). Such adjustment occurs, often gradually, if a child is confronted with counterevidence that cannot be assimilated into their prior theory, either through their own interventions, e.g., in their play, or through observing the interventions of others (Gopnik and Wellman, 2012). According to Gopnik and Meltzoff (1997), children pass through the same developmental processes and therefore have similar representations about the same objects at roughly similar times in their lives. Most children do not test their theories with experiments but adjust them when they are confronted with evidence (Gopnik, 2013). Research has provided support for theory theory, *inter alia*, in the domain of balance (Bonawitz et al., 2012), and biology (Schulz et al., 2007).

Researchers have applied Bayesian inference to the theory theory framework to value the role of probability and prior knowledge on learning processes (Schulz et al., 2007; Gopnik and Wellman, 2012; Gopnik, 2013). Bayesian inference indicates how a learner changes their theory after being confronted with a set of evidence and how children might combine theory and evidence (Schulz et al., 2007). Bayesian models consider prior knowledge and its effect on inductive reasoning as well as how much a person believes one theory to be true. Furthermore, prior beliefs and evidence might interact, e.g., a child might interpret data according to their prior beliefs and dismiss counterevidence (Hawkins et al., 1984; Griffiths et al., 2011; Gopnik, 2013). Children with consistent presumptions will likely change their theory less easily than children with inconsistent presumptions (Gopnik et al., 2001; Sobel et al., 2004; Griffiths et al., 2011).

Studies on theory-evidence coordination have found that young children often face problems relating a theory with evidence (Ruffman et al., 1993; Koerber et al., 2005; Piekny and Maehler, 2013), which seemingly contradicts the results of studies on theory theory (Schulz et al., 2007; Bonawitz et al., 2012). However, taking a closer look at the studies on theory-evidence coordination, these studies showed that young children primarily face problems when evidence was presented in the form of imperfect covariation. Children were more likely to successfully coordinate theory with evidence when the evidence was presented in the form of perfect covariation, which is how the studies on theory theory presented evidence. For example, Sobel et al. (2004) found that children were even able to make inferences from indirect evidence of perfect covariation in the form of data they had not directly observed.

In conclusion, at least three factors might contribute to young children’s science learning with regard to their developmental constraints and should be considered. (1) Children can interpret perfect covariation but face problems with imperfect covariation (Koerber et al., 2005). Therefore, evidence should be presented in the form of perfect covariation. (2) Children have prior theories about science phenomena and often have similar theories at a certain age (Gopnik and Meltzoff, 1997). Therefore, these prior theories should be considered so that the presented evidence relates to children’s intuitive theories. (3) Children with consistent prior theories might need to see counterevidence repeatedly to adjust their theory because this adjustment often happens gradually (Gopnik and Wellman, 2012). Therefore, children should receive enough time to deal with the phenomenon.

The question remains how best to confront young children with evidence relating to their science theories. Children’s developmental constraints can be addressed by allowing for activities that occur in their everyday lives, e.g., play (Copple and Bredenkamp, 2009). Moreover, play might be enriched by scaffolding materials as well as an adult’s verbal support (Zosh et al., 2018).

### Material and Verbal Scaffolds in Guided Play

*Play-based learning* is the mandated pedagogy in early years’ curricula in many countries (Hirsh-Pasek et al., 2009; Pyle et al., 2017) and is regarded as developmentally appropriate practice (Copple and Bredenkamp, 2009). Researchers have widely agreed that play can be characterized as a voluntary, intrinsically motivated, child-directed, and process- rather than goal-oriented behavior that contains elements of choice (Rubin et al., 1983; Pellegrini, 2013; Weisberg et al., 2013; Trawick-Smith, 2015; Daubert et al., 2018).

Zosh et al. (2018) define play as a spectrum that allows for different *types of play*, ranging from *free play* as voluntary, intrinsically motivated, and process-oriented behavior directed by the child to more goal-oriented and adult-directed forms of *playful instruction*. In between these two poles, *guided play* represents a blend of *free play* and *playful instruction* (Zosh et al., 2018). Guided play can be described as a playful activity that is directed by the child, i.e., the child is autonomous to decide what to do, for how long and at what pace. The adult’s role in guided play is to prepare a play environment and support the children’s activities to facilitate learning (Weisberg et al., 2013, 2016; Zosh et al., 2018).

Guided play shares strong commonalities with the guided inquiry principle, which has been identified as one of the most effective approaches to learning and teaching (Mayer, 2004; Alfieri et al., 2011; Lazonder and Kamp, 2012). Researchers have frequently framed guidance in inquiry-based science teaching within the scaffolding construct (Hmelo-Silver et al., 2007). In the scaffolding literature, both material scaffolds and an adult’s verbal scaffolds are considered effective in guiding children’s learning (van de Pol et al., 2010; Martin et al., 2019). Accordingly, guided play can take at least two different forms (Weisberg et al., 2016), guided play with *material scaffolds only*, and guided play with *additional verbal scaffolds*.

In guided play with *material scaffolds*, the adult provides the children with purposefully designed and structured materials aiming at a specific learning objective (Weisberg et al., 2016). Research indicates that children’s explorations with purposefully structured and limited materials can foster science learning (Cook et al., 2011; van Schijndel et al., 2015). In particular, learning materials are effective when they link the learning objective to children’s prior knowledge (Leuchter et al., 2014) and focus children’s attention on those aspects that are essential for understanding (DeLoache, 2014). For example, to foster children’s stability concepts, the adult might provide the children with an assembly of building blocks and a variety of photographs. In guided play with material scaffolds only, the adult initiates the play activities by inviting the children to rebuild the block constructions depicted on the photographs and to explore whether the constructions remain stable or tumble. By building these constructions, the children are likely to face evidence (the construction remains stable or tumbles) that might be incompatible with their intuitive theory (cf. Gopnik and Wellman, 2012). However, beyond initiating children’s explorations, the adult does not intervene in the process.

Research suggests that children show more explorative behaviors when an adult takes a passive role (Bonawitz et al., 2011). In contrast, studies indicate that children’s unguided explorations might not be sufficient to encounter the learning objective (Butts et al., 1994; Chen and Klahr, 1999; Klahr and Nigam, 2004; Sarama and Clements, 2009). In the stability example, children might rebuild the construction inappropriately and thus might witness incorrect evidence or imperfect covariation. Moreover, children might interpret evidence inappropriately to confirm their intuitive but incorrect theory.

In guided play with *additional verbal scaffolds*, the adult not only provides materials but additionally plays along with the children, supports the children’s play verbally and encourages higher order thinking (Chin, 2007; Haden, 2010; Kleickmann et al., 2016; Weisberg et al., 2016; Martin et al., 2019). The adult can use a set of verbal scaffolding techniques to aid children’s cognitive activities (for an overview cf. van de Pol et al., 2010) and support the cognitive processes involved in theory-evidence coordination, thus helping children adjust their intuitive theory. *Activating prior knowledge* by asking questions and prompting the children to express their presumptions, e.g., whether a block construction will remain stable or tumble can facilitate the integration of new aspects into existing schemata (Mayer, 1997; Weinert and Helmke, 1998; Gurlitt and Renkl, 2010). Additionally, *asking for the child’s reasoning*, e.g., by prompting the child to justify their presumptions about the block construction’s stability allows the child to structure their prior knowledge and thinking processes (Hsin and Wu, 2011). *Providing explanations* may help the child coordinate their observations with an evidence-based interpretation of a phenomenon (Murphy and Messer, 2000; Renkl, 2002). *Encouraging comparisons* supports the child in identifying relational similarities or differences between entities by highlighting certain features (Hsin and Wu, 2011; Richey and Nokes-Malach, 2013). Furthermore, *modeling*, i.e., performing certain behaviors and thinking styles, offers a possibility for imitation (Mayer, 2004; Hmelo-Silver et al., 2007).

Research indicates that guided play with *additional verbal scaffolds* promotes children’s science learning more effectively than free play (Pine et al., 1999; Hadzigeorgiou, 2002; Fisher et al., 2011; Reuter and Leuchter, 2020). However, there are only a few studies that have deliberately compared the effectiveness of *material scaffolds* with *additional verbal scaffolds* for children’s science learning. Leuchter and Naber (2019) found that a combination of structured materials and verbal scaffolds supported 6- to 7-year-old children’s learning in the physics domain of force more than only materials, only verbal support or free exploration. Similarly, the results of Hadzigeorgiou (2002) show that 4- to 6-year-olds perform more meaningful activities at an inclined plane to explore the concept of mechanical stability when they received structured materials and verbal scaffolding compared to children who received only materials or played freely.

Studying children’s scientific reasoning in a playful context can aim at unraveling the interplay of material and verbal scaffolds. Concerning children’s reasoning about science phenomena guided play can serve as a developmentally appropriate context to shed a light on (1) children’s theory adjustment, (2) the way their prior theories interact with the evidence provided through the scaffolding materials, and (3) the conditions that may support children to coordinate theory with evidence.

### The Statics Domain and Children’s Beliefs About Balance

Statics can be defined as the state of equilibrium of an object, which in turn is concerned with forces acting on objects that are either at rest or in motion (Riley and Sturges, 1993). Statics is therefore concerned with stability. If the middle of a symmetrical object is supported by a supporting surface, the object will remain stable. Therefore, the consideration of an object’s geometrical center is sufficient when rating symmetrical objects. For an asymmetrical object, however, the mass must be considered because the geometrical center and the center of gravity do not correspond. If the center of gravity of an object is supported, the object will remain in place; however, if it is not supported, the object will tumble. According to Bonawitz et al. (2012); Krist (2010), and Siegler (1976), with increasing age children develop an understanding of the weight distribution so that they can estimate the stability of an asymmetrical object/construction.

Studies with infants have mostly employed the violation of expectation paradigm and have shown that infants have basic knowledge about stability (Baillargeon and Hanko-Summers, 1990). Studies with older children, however, have shown that even preschoolers face problems explaining why certain objects either remain stable or tumble. Krist and Krüger (2012) explain this discrepancy with different approaches of the violation of expectation paradigm and verbal explanations as a possible reason for this ability gap. They state that being surprised (violation of expectation) does not take as much cognitive reasoning as verbally explaining one’s underlying theory.

Young children indeed hold misconceptions about balance. Siegler (1976) and Siegler and Chen (1998) placed different weights at different distances on a fulcrum and asked children to rate the fulcrum’s balance. The researchers found that children from 9 years of age started to consider both weight and distance, while younger children tend to view weight and distance separately. Other studies by Krist et al., have shown that between the ages of three to eight, children’s abilities of balancing symmetrical and asymmetrical blocks and estimating symmetrical as well as asymmetrical objects’ stabilities increase continuously independent of the type of assessment (rating photographs, Krist, 2010; eye tracking, Krist et al., 2018; balancing blocks, Krist et al., 2005). Even though children’s estimation of asymmetrical blocks’ stabilities increased, all three studies found children’s performance on the estimation of symmetrical objects (e.g., cuboids) to be superior to their estimation of asymmetrical objects (e.g., L-shaped objects). As noted earlier, the center of gravity does not correspond to the geometrical center of an asymmetrical object. For symmetrical objects, however, considering their geometrical center is sufficient. Thus, children’s difficulty in estimating the stability of asymmetrical objects indicates that they face problems considering the weight distribution.

Some studies have taken a closer look at children’s theories about balance. Pine et al. (2007) asked 6- to 8-year-old children about their reasoning when balancing beams on a fulcrum and categorized their verbal utterances as well as their gestures into four categories: middle, weight, distance, and other. They found that most answers fell into the other or weight categories, and few children considered the distance. Moreover, Weber and Leuchter (2020) found that more than half of the 5- and 6-year-olds in their sample used an undifferentiated pattern when rating photographs of asymmetrical objects, approximately 1/3 applied Center knowledge, and less than 10% of children applied Mass knowledge.

The above studies have examined children’s knowledge about stability from a developmental psychological perspective. However, it is also of interest if children’s Mass knowledge can be supported in regard to their developmental constraints. Playful interventions with building blocks have supported the acquisition of different mathematical and spatial skills in other studies (e.g., Ferrara et al., 2011; Fisher et al., 2013; Borriello and Liben, 2018; Thomson et al., 2018; Verdine et al., 2019).

Regarding children’s rating of stabilities, Pine and Messer (2003) found that 5- to 6-year-olds were able to balance more symmetrical as well as more asymmetrical blocks after playing with the blocks compared to a pretest. In another study, children between 4 and 7 years of age first balanced symmetrical and asymmetrical objects on a beam scale (Bonawitz et al., 2012), and their balancing behavior was categorized into three categories (*No*, *Center*, and *Mass theory*). Furthermore, the results indicated that younger children tend to use an undifferentiated pattern (No theory) and do not consider the center or the mass. Second, after balancing objects on a beam, children either played with a mass-consistent or a center-consistent toy on their own and freely. Afterward, they again balanced an asymmetrical block. Children who had a Center theory before playing observed evidence that did not confirm their theory if they were in the mass condition. Many of these children adopted a Mass theory. Children who had a Mass theory before playing also observed evidence that did not confirm their theory if they were in the center condition. Most of these children did not alter their balancing behavior and instead explained away the evidence and remained Mass theorists. This outcome indicated that even a short presentation of counterevidence can support children’s learning, but that their prior theories need to be considered.

The different effects of free play and guided play with material and material + verbal scaffolds on children’s science learning in the domain of balance with regard to their prior theories have not yet been investigated. Furthermore, it remains unclear whether these adjustments remain stable over a longer period of time or if the children relapse into their prior intuitive theories.

### Possible Relationship With Intelligence

Intelligence is one of the most important prerequisites for learning. The ability to solve or complete puzzles or patterns is considered an indicator of fluid intelligence (Cattell, 1987; Flynn and Blair, 2013). Two components of fluid intelligence are figural perception and figural reasoning as indicators of an individual’s ability to perceive and mentally represent objects and abstract certain characteristics (Cattell, 1987; Weiß and Osterland, 2013). In the context of stability, figural perception and figural reasoning might contribute to children’s Mass theory. To rate stabilities correctly, children must perceive, mentally represent and abstract the spatial features of the objects or constructions (Weber and Leuchter, 2020).

Language capacity is considered an indicator of crystallized intelligence and is one of the key indicators of mental ability in young children (Cattell, 1987; Flynn and Blair, 2013). Language capacity contributes to knowledge acquisition (Thorsen et al., 2014). Moreover, children with a higher language capacity might find it easier to articulate their reasoning and might profit more from verbal scaffolds.

### The Present Research

The present study is concerned with the effects of three different types of construction play on children’s science learning in the statics domain, specifically constructions’ stabilities. We implemented two types of guided play (verbal + material scaffolds, material scaffolds) and free play. Following the literature on theory theory, Bayesian inference, and theory-evidence coordination, young children’s science learning may be fostered by confronting children with evidence in the form of perfect covariation (Koerber et al., 2005; Gopnik and Wellman, 2012). Furthermore, children’s prior theories, which they have acquired through their everyday activities, e.g., their play, should be considered so that the presented evidence relates to these theories, which can then help children interpret the evidence (Gopnik and Meltzoff, 1997; Gopnik and Wellman, 2012). For example, at the age of 5 to 6, children might explain and predict the stability of an object with a Center theory or have other theories (Bonawitz et al., 2012; Weber and Leuchter, 2020). Material scaffolds can be prepared in such a way that they show perfect covariation for Mass theory and contradict Center theory. Through verbal scaffolding, an adult can help the children connect the evidence presented through the material scaffolds with their prior (intuitive) theories. Thus, scaffolds may support children’s theory adjustment from Other^1^ or Center theory to Mass theory. Since theory adjustment often happens gradually (Gopnik and Wellman, 2012), children should receive enough time to explore stabilities. We designed playful interventions that consider these constraints and investigated the effects of the different kinds of play on children’s theory adjustments in the statics domain. Moreover, we explored whether these adjustments remained stable over an extended period of time.

Finally, interindividual prerequisites might be partly responsible for children’s theory adjustment and interact with the type of playful intervention that the children received. Research on theory theory, Bayesian inference and theory-evidence coordination suggests that children with a consistent prior theory might not adjust their theories as easily as children with an inconsistent prior theory (Koerber et al., 2005; Gopnik and Wellman, 2012). Thus, we are interested in the contribution of children’s prior theories on their adjustments after being confronted with perfect evidence for Mass theory. Additionally, intelligence affects learning (Cattell, 1987; Flynn and Blair, 2013; Thorsen et al., 2014) and may thus contribute to theory adjustment as well. With respect to fluid intelligence, we hypothesize that figural perception and figural reasoning facilitate theory adjustment. With respect to crystallized intelligence, we hypothesize that children with higher language capacity might profit more from verbal scaffolds than children with lower language capacity.

Therefore, we specify the following research questions:

(1) Do children explain their reasoning about stability with Mass theory, Center theory or Other?
(2) Can guided play with material scaffolds and with or without verbal scaffolds enhance children’s consistent use of Mass theory compared to free play (a) directly after a playful intervention and (b) over an extended period of time? Does intelligence relate to the consistent use of Mass theory?
(3) Does the consistency of children’s prior theories relate to children’s consistent use of Mass theory in the different play conditions (a) directly after a playful intervention and (b) over an extended period of time? Does intelligence relate to the consistent use of Mass theory when prior theories are considered?
(4) Do children with a consistent Mass theory after the playful intervention perform differently on a transfer test than children who did not use Mass theory consistently? Does intelligence relate to performance on the transfer test?

## Materials and Methods

### Participants

In total, 183 children (88 girls, 95 boys), between the ages of five and six (*M* = 5.55, *SD* = 0.51), took part in the study. The participants visited 23 kindergartens in Germany (2 to 13 children per kindergarten), which all agreed to take part in the study and helped connect with the children and their parents. The kindergartens were located either in villages (700 to 3,000 inhabitants; *N* = 83 children), small cities (less than 20,000 inhabitants; *N* = 10 children) or medium sized cities (approximately 50,000 inhabitants; *N* = 91 children of whom 51 lived in the city center and 40 in the periphery). A total of 171 children were European, 9 were Asian, 2 were African, and 1 was Central American. All children and their parents were informed about the goals of the study, and all children took part voluntarily and with their parents’ consent.

Some children dropped out of the study completely because, e.g., they moved or were ill on the dates agreed with the kindergartens, and some other children had missing values on some of the items. We used pairwise deletion because we decided to include the highest amount of data whenever possible. Therefore, the number of participants varies between different analyses.

### Procedure

The study consisted of a pre-post-follow-up design with two guided play groups and a free play group. The pretest (T1) took place approximately 2 weeks before the play session and the immediate posttest (T2). The follow-up (T3) took place approximately 10 weeks after the posttest. The duration of the play session was approximately 1 h.

For the intervention, the children were parallelized into the three intervention groups according to their language capacity, which was assessed at pretest. Thus, matched samples were produced and each child in the Verbal group had a “language capacity twin” in the Material and in the Free play group. Both the *Material group* (59 children, 32 girls, and 27 boys) and the *Verbal group* (64 children, 27 girls, and 37 boys) received scaffolding materials in the form of building blocks. The Verbal group received additional verbal scaffolds. The *Free play group* (60 children, 29 girls, and 31 boys) played with building blocks freely. The reason for the differences in group size is that each intervention was to be conducted in a group of approximately four to six children to achieve ecological validity. Therefore, five children in the Verbal group were not assigned to language capacity triplets in the other two groups. In total, there were 51 interventions with group sizes varying between 2 to 6 children per group.

The play was led by one of six female experimenters. To prevent experimenter effects, group^∗^experimenter was varied, so that all experimenters had led all intervention groups, i.e., Verbal, Material, and Free play group. In all intervention groups, the children were free to choose what they wanted to build or if they wanted to build with a friend or rather on their own. Furthermore, breaks were always possible, and the children were free to stop playing entirely (cf. Rubin et al., 1983; Weisberg et al., 2016). For manipulation check, the play sessions were video or audio recorded with the permission of parents and children. Based on the recordings, we rated children’s playfulness according to Bundy et al. (2001) as well as children’s on-task behavior. High inferential ratings showed that all children in all recordings showed indications of playfulness, e.g., children sang, laughed, joked around with another or the experimenter, chose to build challenging constructions, and built together. Moreover, children’s on-task behavior did not differ between the groups.

Children in the Material group and the Verbal group received photographs of different block constructions, which differed in the number of blocks used and in their complexity. With each photograph came a box containing the building blocks needed for building the construction. The blocks differed in their shapes (cuboids, triangles, etc.), sizes, and colors (brown, black, yellow, red, and green). The materials were developed prior to this study and tested in play sessions to ensure that children were able to rebuild the structures shown on the photographs and had fun playing with the materials. The material scaffolds were implemented to structure the play and served as suggestions for the children. However, the children in the guided play groups were free to build constructions other than those we presented to them. In order for the activity to be enjoyable and playful for the children, we allowed the children to decide whenever they wanted to move on to the next activity. However, the experimenters could suggest another activity if they felt that the children started to lose interest.

The material scaffolds encompassed five activities and were presented in a standardized order (cf. Supplementary Material 1 for all photographs):

(1) Black block (11 photographs): You can build the building shown on the photograph. Build the building and guess if the blocks remain stable or tumble.
(2) Add-a-block (8 photographs): These blocks on the photos were bewitched so they would remain stable. Can you rebuild the building, so that it is stable? (If a child did not succeed, the experimenter provided a green block): Look, here is a green block. Try to stabilize the building with it.
(3) Sliding (9 photographs): This is the sliding play. You rebuild the building on the photograph. Then, you slide the upper block along the lower block until it falls (experimenter models it). That’s noisy, isn’t it?
(4) Rebuild (11 photographs): You can just rebuild the building on these photographs and see how well you are doing. Some buildings are very easy to rebuild; others are more difficult. However, every single one will remain stable if built correctly.
(5) Stable/Tumble (8 photographs): The buildings on the photographs will sometimes remain stable, but at other times, the blocks are bewitched. Look at the photograph and predict “stable” or “tumble,” and then try them out to see whether you were correct.

Additionally, the Verbal group received verbal scaffolds in the form of the activation of prior knowledge, asking for reasoning, the provision of explanations, the encouragement of comparisons, and modeling (cf. Table 1; Hogan and Pressley, 1997; van de Pol et al., 2010; for the script cf. Supplementary Material 2). The experimenters used this limited set of scaffolds presented in the script but applied them flexibly when playing with the children. All experimenters had received a training on how to apply scaffolding during play prior to leading the interventions.

**TABLE 1:** Scaffolding techniques used in the Verbal group (Hogan and Pressley, 1997; van de Pol et al., 2010).

**Technique**	**Example**
Activating prior knowledge	Have you ever seen something like this?
Asking for reasons	Can you explain this in more detail, so I can really understand what you think?
Providing explanations	Well done! If the heavy side of a block hovers in midair, the block will tumble
Encouraging comparisons	Your building looks different than [another child’s building], doesn’t it? What is different? Is something similar?
Modeling	Look! (Experimenter also looks very closely/experimenter shows how to build a certain building)

The Free play group received a large box with the same building blocks as the guided play groups, but the blocks were unstructured. The experimenters did not suggest any buildings that the children could build but only told the children to play with the blocks freely.

During the play time, the experimenter praised the children’s efforts in all three groups and motivated them to try again if they encountered problems with building. Sometimes children would ask the experimenter for help with building, which she would provide in the Verbal group. However, in the Material group or the Free play group, she would friendly decline and suggest that children ask another child for help (cf. Supplementary Material 3 for excerpts from the playful intervention).

### Measures

#### Children’s Theories

Children’s theories about stability were assessed with a standardized single interview consisting of photographs of four symmetrical and four asymmetrical block constructions, which were always supported by a black block (cf. Supplementary Material 4). The children were asked to estimate whether the block construction presented in the first photograph would remain stable or not if the black block was removed. Afterward, the children received a total of five blocks, namely, four cuboid blocks consisting of two brown blocks, one black block and one yellow block (9 cm^∗^3 cm^∗^1.5 cm), as well as one smaller black cuboid block (3 cm^∗^3 cm^∗^1.5 cm). All the blocks were made of non-lacquered wood and colored by the researchers using acrylic colors to avoid slipperiness. The brown blocks had a narrow line drawn onto them with a pencil to facilitate finding the blocks’ middle for the children. The children were asked to rebuild the construction presented in the photograph. Then, the interviewer repeated the children’s former answer regarding the construction’s stability (stable or unstable) and asked them to explain the prediction. They answered, and the interviewer invited them to remove the black block and ascertain whether they had rated the stability correctly. Then, they proceeded to the second block structure and so on. The interviews were videotaped or recorded if both the parents and the child had consented to it; if not, the interviewer made notes on the child’s theory. The same interview was administered at each point of measurement, i.e., the same test items were presented at T1, T2, and T3. The testing time was approximately 10 min.

Only the asymmetrical block constructions were used in the data analyses. The test started with two symmetrical items to familiarize the children with the test logic. The fourth and sixth items showed symmetrical constructions and were applied to ensure that children had positive mastery experiences during the testing because studies have found that symmetrical items’ stabilities are easier for children to estimate than asymmetrical items’ stabilities (Krist, 2010). The three asymmetrical items showed perfect covariance for Mass theory because the weight distribution always determined the stability. However, the second asymmetrical item as well as all the symmetrical items could also be rated correctly with Center theory, while the first and third asymmetrical items could not. Therefore, the evidence for Center theory was imperfect.

The children’s answers were coded following the speech coding scheme from Pine et al. (2007), as shown in Table 2. If a child was unable to verbalize their answer, but, e.g., pointed at the vertical block, their answer was also rated as Mass theory. If a child indicated the middle with gestures, the answer was rated as Center theory. The children’s explanations were coded by two independent raters, Cohen’s κ > 0.90.

**TABLE 2:** Coding scheme.

**Coding**	**Speech**	**Example**
Mass theory	The child refers to the weight being on one side of the brown blocks, mentions heaviness or talks about the importance of the vertical block	“This side is heavier.” “It’s because of the block that’s standing on the other”
Center theory	The child refers to the middle of the block or a bigger amount of the block resting on either the black or the yellow block	“The brown block is resting more on the yellow block”
Other	Child speaks of something other than the two variables of interest (weight, middle), e.g., refers to the color	“I don’t know.” “It tumbles, because it tumbles”

**FIGURE 1:**
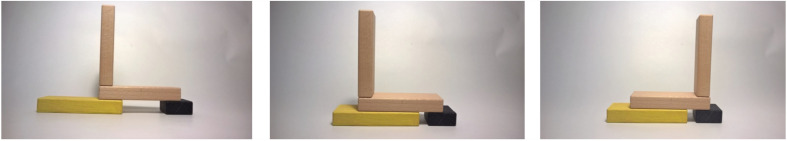
The asymmetrical constructions used to assess children’s reasoning. Item 1 and 2 are stable constructions, item 3 is an unstable construction.

Regarding the items that were not used for further analysis, the distribution of the children’s answers at T1 was as follows. For the first item (familiarization, symmetrical), 18 Mass, 118 Center, and 39 Other. For the second item (familiarization, symmetrical), 14 Mass, 124 Center, and 37 Other. For the fourth item (motivation, symmetrical), 19 Mass, 115 Center, and 42 Other. For the sixth item (motivation, symmetrical), 15 Mass, 96 Center, and 64 Other. The seventh item showed an asymmetrical construction but was removed from further analyses because it was inconclusive. The probability of the item remaining stable was 50% for statics reasons. Thus, there is no definite answer to this item. The third, fifth, and eighth items were asymmetrical items that were included in the data analyses and used for the assessment of children’s stability theories (cf. Figure 1).

#### Transfer Test

**FIGURE 2:**
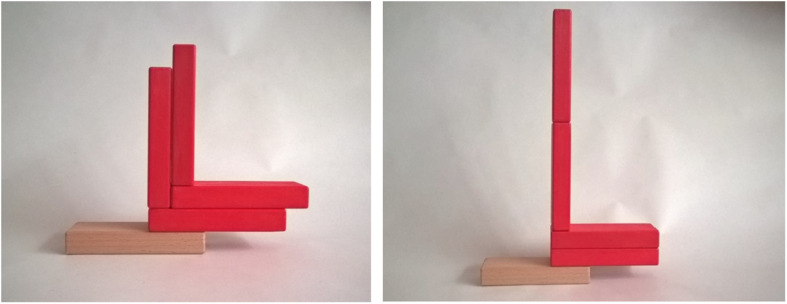
Example items of the transfer test, both items show stable constructions.

At the third point of measurement, a paper-pencil transfer test consisting of photographs of 16 asymmetrical block constructions was administered (cf. Figure 2 and Supplementary Material 5 for all transfer items), i.e., 8 stable constructions and 8 unstable constructions. The test was conducted in a group of up to six children who were seated back-to-back to prevent them from copying from each other. The test took approximately 10 min. The children were asked to rate the constructions’ stabilities by either circling the photograph for a stable construction or crossing out the photograph for an unstable construction. Thus, children were not required to verbalize their knowledge. The constructions could only be rated correctly by considering the weight distribution because if the center of gravity was supported by the brown block but the geometrical center was not, then the constructions would always remain stable. However, if the center of gravity was not supported but the geometrical center was, then the construction would always tumble. For this instrument, the children did not need to explain their reasoning and only had to rate photographs. The children’s content knowledge could be assessed to support the results of the reasoning test.

#### Aspects of Intelligence

Visual perception and figural reasoning as two aspects of fluid intelligence were assessed with the labyrinths and matrices subtests of the Culture Fair Test (CFT 1-R; Weiß and Osterland, 2013) at T1. *T*-values were not available, as only two subtests were conducted (for more information on test parameters, please cf. CFT 1- R handbook).

Language capacity as an indicator for crystallized intelligence was assessed with the German version of the Peabody Picture Vocabulary Test (PPVT 4; Dunn et al., 2015) at T1. The PPVT is a picture-based standardized single interview for which *t*-values are available. The test consists of 19 sets with 12 items each consisting of four pictures per item. Five-year-old children start with set 4, and 6-year-old children start with set 5. For each item, the children receive a word and point at the corresponding picture. This procedure is continued until the child answers 8 out of 12 items in a set incorrectly (cf. PPVT 4 handbook for more information). The PPVT 4 was also administered to ensure that all children understood and spoke the German language sufficiently.

The total testing time was approximately 1 h at T1, 10 min at T2 and 20 min at T3. At T3, the follow-up test was administered before the transfer test. During the testing, breaks were possible whenever the child or the experimenter considered it necessary.

### Data Analyses

The statistics program R, version 3.6.2 (R Core Team, 2019) was used for data analyses. We used the survival (Therneau, 2015) package for the specification of survival analyses and the survminer (Alboukadel et al., 2019) package for forest plots.

In the first step, we investigated the number of children who had a Mass theory, a Center theory or Other on each point of measurement. The children received feedback about the correctness of their stability rating because after rating the stability, they removed the black block and could ascertain if they had answered correctly. Therefore, they had the opportunity to learn during testing. Thus, their answers were not independent and could not be summarized but instead were treated as individual events as the assumption of local stochastic independence was violated. Therefore, we used methods of risk-event analysis to analyze the group differences in the application of Mass theory (cf. Singer and Willett, 2003).

## Results

### Children’s Use of Mass Theory

**FIGURE 3:**
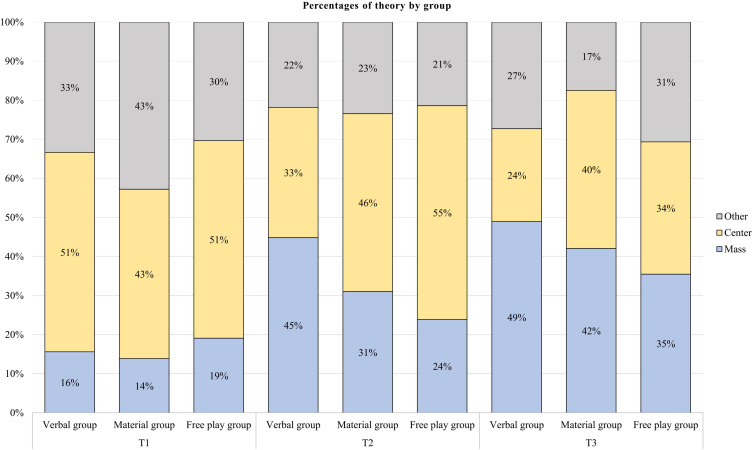
Percentages of theories applied by the children to explain all three reasoning items’ stabilities per measurement point and intervention group.

To address the first research question concerned with children’s use of causal relations, especially Mass theory, when explaining asymmetrical objects’ stabilities, we investigated the percentage of children who applied mass for explaining each of the three asymmetrical constructions on the first point of measurement. The following results were obtained across all groups. For the first item, 11%^2^ of children explained their reasoning with Mass theory, 41% with Center theory, and 48% provided another answer. For the second item, 20% of children explained their reasoning with Mass theory, 56% with Center theory, and 23% provided another answer. For the third item, 16% of children explained their reasoning with Mass theory, 48% with Center theory, and 35% provided another answer, cf. Figure 3 for percentage shares of children’s theories (cf. Supplementary Material 6 for percentages of correct answers). To compare the probability of answering with a specific theory between items, we compared the proportions of children who had answered with a specific theory (either Mass, Center or Other) using z-tests of proportions. They revealed that children were not more likely to explain their reasoning with Mass for any of the three items, *z* = 5.41, *df* = 2, *p* = 0.067; however, the answer probabilities for Center theory, *z* = 8.87, *df* = 2, *p* = 0.012, and Other, *z* = 23.90, *df* = 2, *p* < 0.001, differed across the items.

The percentage of children applying each theory for each group at T1, T2 and T3 is presented in Table 3. The use of Mass theory increased at T2 and T3, especially in the Verbal group.

**TABLE 3:** Number of children applying each theory in each group over all three points of measurement.

		**Verbal group (*N* = 64)**			**Material group (*N* = 59)**			**Free play group (*N* = 60)**		
	**Item**	**Other (%)**	**Center (%)**	**Mass (%)**	**Other (%)**	**Center (%)**	**Mass (%)**	**Other (%)**	**Center (%)**	**Mass (%)**
T1	1	47	40	13	51	40	9	47	42	12
	2	24	60	16	30	50	20	15	59	25
	3	29	53	18	49	40	11	29	51	20
T2	1	38	25	38	32	43	25	34	51	15
	2	14	39	46	13	53	34	9	60	30
	3	13	36	51	25	40	35	21	53	26
T3	1	35	23	42	40	36	24	48	29	23
	2	15	27	58	10	36	55	8	46	46
	3	32	21	47	2	50	48	23	54	23

*Percentages are given in rounded numbers.*

### Effects of Guided Play

To address the second research question concerning whether different kinds of play can enhance children’s consistent use of Mass theory directly after the intervention as well as over a longer period of time, we used methods of survival analyses. Survival analysis is used to analyze the expected duration of time until an event takes place. In our case, the event is the children’s consistent use of Mass theory after the playful intervention.

#### Consistency at T2

First, we used the binomial distribution to find a cut-off that guarantees that the probability of children finding the correct answer through guessing was below 10%. This enabled us to find how many correct answers might be given through guessing with σ = 1.64, i.e., *p* < 0.10, and a binomial probability of 1/3. Thus, we are able to categorize the children into children who explained their reasoning with Mass theory either consistently or inconsistently directly after the intervention at T2. Children with 3 out of 3 correct answers were rated as answering consistently, *p* = 0.037. Seven children who had explained their reasoning with Mass theory consistently at T1 (3 out of 3 Mass answers) were excluded from these analyses.

We defined each item as a point in time; therefore, time = 1 refers to the first item of T2, time = 2 to the second item of T2, and time = 3 to the third item of T2. The event of answering with Mass theory 3 out of 3 times could only take place at time = 3 or not at all. The detailed results of the Kaplan-Meier analysis are presented in Supplementary Material 7. The survival rate implies the percentage of children who remain either Center theorists or Other, and thus are not applying Mass theory consistently at T2. Therefore, the rate of children who applied Mass theory consistently is 100% minus survival rate, e.g., 100%-77% for the Verbal group. The results indicated that 23% of the children in the Verbal group explained their reasoning with Mass consistently at T2 compared to 9% of children in the Material group and 6% of children in the Free play group.

To investigate the results of the Kaplan-Meier analysis with a stricter procedure and to include the contributions of metric predictors, i.e., fluid and crystallized intelligence, a Cox regression (cf. Table 4) was specified, likelihood ratio test = 12.87, *p* = 0.012. The Cox proportional hazard model assumes that the hazard curves for the groups should be proportional. This means that if child 1 is twice as likely to explain their reasoning with Mass theory than child 2 at an initial point in time, then at all later points in time, child 1 remains twice as likely to explain their reasoning with Mass theory consistently compared to child 2. In this particular Cox-regression, the event could only take place at time = 3. Therefore, the proportional hazard assumption was met.

**TABLE 4:** Cox-regressions for children’s acquisition of Mass theory at T2.

					**95% CI**	
	***b***	***SE***	***z***	**HR**	**LL**	**UL**
**Development of consistencies between groups (Cox-regression)**						
ΔFree play–Verbal	1.31*	0.65	2.01	3.70	1.03	13.26
ΔMaterial–Verbal	0.69	0.58	1.18	1.99	0.63	6.26
ΔFree play–Material	0.62	0.76	0.81	1.86	0.42	8.31
Fluid intelligence	0.04	0.06	0.69	1.04	0.93	1.16
CrI	0.08*	0.03	2.41	1.08	1.01	1.15
**Interaction of crystallized intelligence with intervention group**						
ΔFree play–Verbal*CrI	–0.05	0.11	–0.44	0.95	0.76	1.19
ΔMaterial–Verbal*CrI	0.15*	0.07	2.15	1.67	1.01	1.34
ΔFree play–Material*CrI	–0.20	0.12	–1.71	0.82	0.65	1.03
CrI free play	0.16	0.10	1.58	1.18	0.96	1.44
CrI material	–0.04	0.06	–0.68	0.96	0.86	1.08
CrI verbal	0.11**	0.04	2.65	1.12	1.03	1.22
Fluid intelligence	0.05	0.06	0.87	1.05	0.94	1.18

CrI, crystallized intelligence; HR, hazard ratio; CI, 95% confidence-interval; LL, lower level; UL, upper level. * *p* < 0.05. ** *p* < 0.01.

The Cox regression showed group differences in the consistency of answering with mass between the Free play group and the Verbal group. However, there were no differences between the Material group and the Verbal group or the Free play group and the Material group. The Verbal group was almost four times as likely to explain their reasoning with Mass theory consistently compared to the Free play group, as indicated by the regression coefficient, and by a factor of HR = 3.70. Although neither the differences between the Verbal group and the Material group nor those between the Material group and the Free play group were statistically significant, descriptively, the Cox regression implied that the Verbal group had twice the chance of applying Mass theory consistently than the Material group, HR = 1.99 and that the Material group had approximately twice the chance than the Free play group, HR = 1.86. Crystallized intelligence contributed to the consistent application of Mass theory, while fluid intelligence did not.

We tested whether crystallized intelligence interacted with the children’s Mass theory in the three intervention groups. The analysis showed a difference between the Material group and the Verbal group dependent on crystallized intelligence, crystallized intelligence^∗^ΔMaterial–Verbal, *b* = 0.15, *p* = 0.032. This indicates that children with high crystallized intelligence in the Verbal group profited more than children with high crystallized intelligence in the Material group (Table 5).

**TABLE 5:** Cox-regressions for children’s acquisition of Mass theory at T3 considering T2.

					**95% CI**	
	***b***	***SE***	***z***	**HR**	**LL**	**UL**
**Development of consistencies between groups (Cox-regression) over T2 and T3**						
ΔFree play–Verbal	1.22**	0.45	2.74	3.40	1.42	8.16
ΔMaterial–Verbal	0.57	0.41	1.39	1.77	0.79	3.95
ΔFree play–Material	0.66	0.51	1.30	1.93	0.72	5.18
Fluid intelligence	0.10*	0.04	2.48	1.11	1.02	1.20
CrI	0.06*	0.02	2.56	1.06	1.01	1.11
**Interaction of crystallized intelligence with intervention group**						
ΔFree play–Verbal* CrI	–0.02	0.06	–0.37	0.98	0.87	1.10
ΔMaterial–Verbal* CrI	0.14*	0.05	2.57	1.15	1.03	1.28
ΔFree play–Material* CrI	−0.16*	0.07	–2.30	0.85	0.74	0.98
CrI free play	0.11*	0.05	1.99	1.11	1.00	1.23
CrI material	–0.06	0.05	–1.25	0.94	0.86	1.03
CrI verbal	0.08**	0.03	2.61	1.09	1.02	1.16
Fluid intelligence	0.11**	0.04	2.61	1.12	1.03	1.21

CrI, crystallized intelligence; HR, hazard ratio; CI, 95% confidence-interval; LL, lower level; UL, upper level. * *p* < 0.05. ** *p* < 0.01.

#### Consistency Over T2 and T3

**FIGURE 4:**
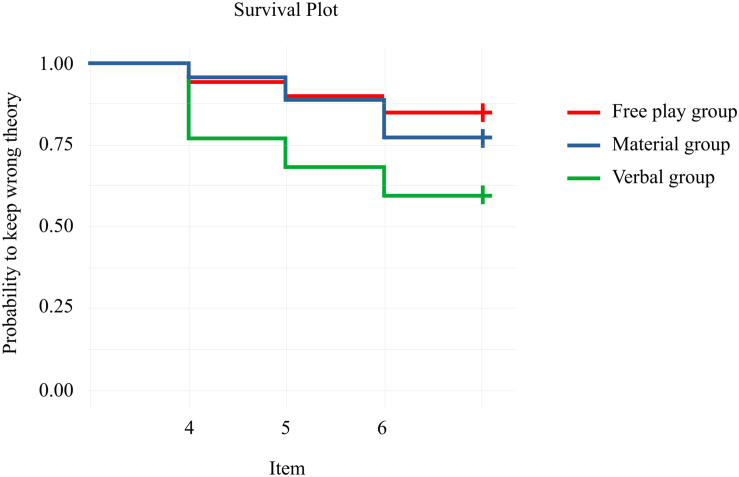
Survival curves of the three play groups.

Next, we were interested in whether children’s answers differed in their consistency over a longer period of time to check if the effect of the guided play was lasting. Therefore, we combined the three items of T2 and T3 into 6 points in time. Again, we used the binomial distribution with σ = 1.64, *p* < 0.10, and binomial probability = 1/3 to categorize the children into children who explained their reasoning with Mass theory either consistently or inconsistently. Children with ≥4 Mass explanations out of 6 when combining the items of the posttest and the follow-up were categorized as answering consistently, *p* = 0.097. The first point in time on which the event could take place was time = 4, i.e., the first item of T3, because the children had to answer four items with Mass theory to fulfill the event. The event could also take place at time = 5, i.e., the second item of T3, and at time = 6, i.e., the third item of T3. We specified a Kaplan-Meier analysis to investigate the percentage of children using Mass theory consistently (cf. Figure 4 and Supplementary Material 7). Extending the descriptive results, the children in the Verbal group had the highest percentage of using Mass theory consistently at each point in time compared to the other two groups, Verbal group = 40%, Material group = 23%, Free play group = 15%.

**FIGURE 5:**
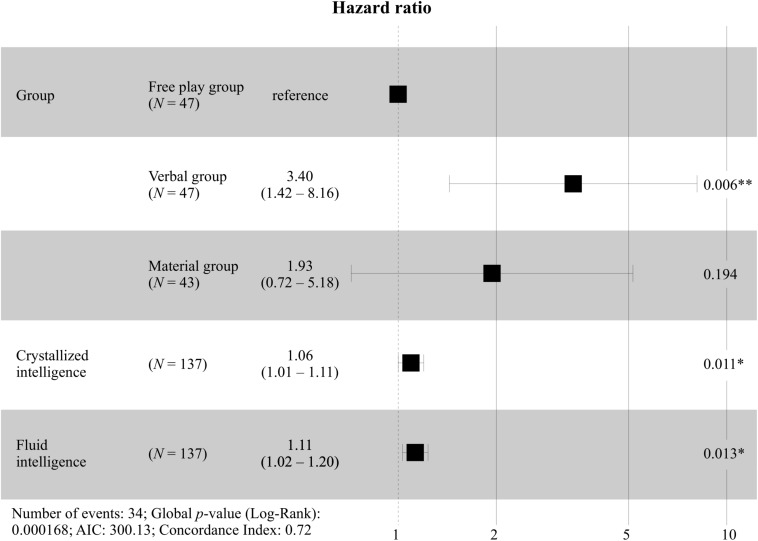
Forest plot with hazard ratios for the intervention groups including T2 and T3. ^∗^*p* < 0.05. ^∗∗^*p* < 0.01.

Next, a Cox regression (cf. Table 5) was specified, likelihood ratio test = 22.38, *p* < 0.001. First, we tested the proportional hazard assumption by correlating the scaled Schoenfeld residuals for group membership with time to ensure that the time and the residuals were independent. The hazard curves for the groups were proportional, as indicated by the global test, χ^2^ = 5.56, *p* = 0.234, as well as the group comparisons, all *p* > 0.05. The Cox regression showed group differences between the Verbal group and the Free play group, with the Verbal group having a higher chance of explaining their reasoning with Mass theory consistently by factor HR = 3.45. Again, there were no group differences between the Material group and the Free play group or between the Material group and the Verbal group. However, descriptively, the Verbal group had the highest chance of explaining their reasoning with Mass theory. Fluid and crystallized intelligence contributed to the consistent explanation with Mass theory. For the hazard ratios, cf. Figure 5.

Again, the interaction of crystallized intelligence and the intervention group was included in the Cox regression (cf. Table 5). Crystallized intelligence contributed to the consistent use of Mass theory in the Verbal group and in the Free play group but not in the Material group. These differences were statistically significant. For low crystallized intelligence, the children in the Material group profited most from the intervention compared to the Verbal group and the Free play group.

### Relationship of Children’s Theory at T1 and Children’s Consistent Use of Mass Theory

To address the third research question concerned with the relationship of the children’s prior theories and their consistent use of Mass theory after the playful intervention, we categorized the children into those answering consistently or inconsistently at T1. For this method, we used the same criterion used for the prior analyses, i.e., children explaining their reasoning with either Center theory or Other 3 out of 3 times, σ = 1.64, *p* < 0.10, binomial probability of 1/3, were categorized as answering consistently at T1, and the other children were categorized as answering inconsistently. Hence, for the following analyses, the sample was divided into six groups, i.e., a consistent and inconsistent group for each of the three intervention groups. For categorizing children consistently answering with Other, we considered those children who had provided a theory neither concerned with the center nor the mass of constructions for all three items of the pretest.

For the survival analysis, we applied the criterion of children explaining their reasoning with ≥4 Mass out of 6 to investigate whether children’s prior theories relate to their acquisition of Mass theory. Kaplan-Meier analysis indicated that the children in the Verbal group who had answered inconsistently at T1 had the highest chance of explaining their reasoning with Mass consistently after the guided play, with 62% (cf. Supplementary Material 7 for the comprehensive results).

**FIGURE 6:**
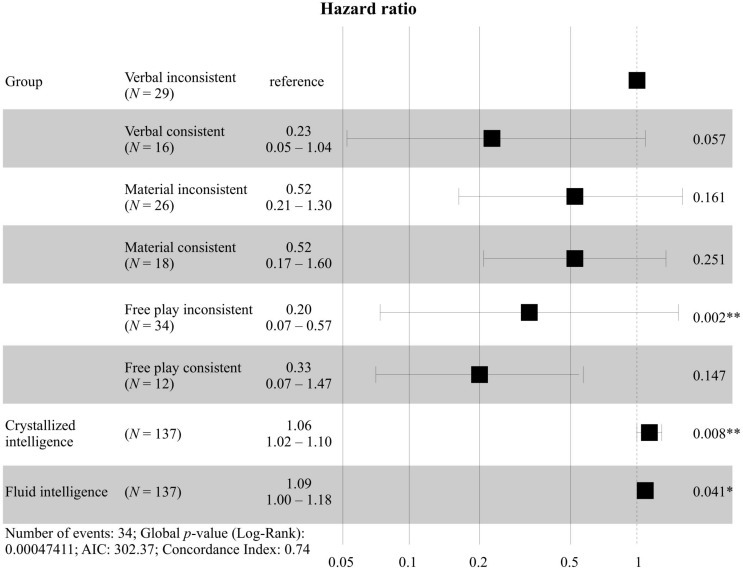
Forest plot with hazard ratios for the intervention groups in consideration of children’s prior theories and including T2 and T3. ^∗^*p* < 0.05. ^∗∗^*p* < 0.01.

Next, a Cox regression (cf. Table 6) was specified, likelihood ratio test = 26.15, *p* < 0.001. The hazard curves for the groups were proportional, as indicated by the global test, χ^2^ = 7.13, *p* = 0.416, as well as the group comparisons, all *p* > 0.05. We decided to use the Verbal group children who had answered inconsistently at T1 as the reference group for the Cox regression because theory suggests that this group should have the highest probability of explaining their reasoning with Mass theory. We found that this group had a significantly higher probability of explaining their reasoning with Mass theory than the Free play group children who had answered inconsistently at T1. Furthermore, descriptively, the Verbal group children who had answered inconsistently at T1 had the highest probability of all groups for explaining their reasoning with Mass theory (cf. Figure 6). We found no differences between the Free play group children who had answered consistently at T1 and any of the other groups. Fluid and crystallized intelligence contributed to the consistent use of Mass theory for all groups.

**TABLE 6:** Development of Mass theory consistencies between groups (Cox-regression) at T3 considering T2.

					**95% CI**	
	***b***	***SE***	***z***	**HR**	**LL**	**UL**
**Verbal inconsistent as the reference group**						
ΔVerbal inconsistent– Verbal consistent	–1.46	0.77	–1.90	0.23	0.05	1.04
ΔVerbal inconsistent– Material inconsistent	–0.66	0.47	–1.40	0.52	0.21	1.30
ΔVerbal inconsistent– Material consistent	–0.66	0.58	–1.15	0.52	0.17	1.60
ΔVerbal inconsistent– Free play inconsistent	−1.59**	0.53	–3.03	0.20	0.07	0.57
ΔVerbal inconsistent–Free play consistent	–1.11	0.76	–1.45	0.33	0.07	1.47
FI	0.08*	0.04	2.05	1.09	1.00	1.18
CrI	0.06**	0.02	2.67	1.06	1.02	1.10
**Free play consistent as the reference group**						
ΔFree play consistent–Verbal inconsistent	1.11	0.76	1.45	3.02	0.68	13.46
ΔFree play consistent–Verbal consistent	–0.35	1.01	–0.35	0.70	0.10	5.07
ΔFree play consistent–Material inconsistent	0.45	0.81	0.55	1.56	0.32	7.64
ΔFree play consistent–Material consistent	0.45	0.87	0.51	1.56	0.28	8.65
ΔFree play consistent–Free play inconsistent	–0.49	0.85	–0.58	0.61	0.12	3.22
FI	0.08*	0.04	2.05	1.09	1.00	1.18
CrI	0.06**	0.02	2.67	1.06	1.02	1.10

FI, fluid intelligence; CrI, crystallized intelligence; Δβ, difference in regression coefficient between two groups. HR, hazard ratio; CI, 95% confidence-interval; LL, lower level; UL, upper level. * *p* < 0.05. ** *p* < 0.01.

### Transfer Test

To address the fourth research question concerned with children’s performance on the transfer test, we compared children who had explained their reasoning with Mass theory consistently at T2 and T3, *M* = 11.44, *SD* = 3.50, to children who had not explained their reasoning with Mass theory consistently at T2 and T3, *M* = 7.41, *SD* = 4.16. Regardless of the intervention group, those children who had explained their reasoning with Mass theory consistently at T2 and T3 performed better on the transfer test than the other children, *t*(65.83) = −5.26, *p* < 0.001. A multiple regression analysis showed that neither fluid, *b* = 0.05, *p* = 0.571, nor crystallized intelligence, *b* = 0.04, *p* = 0.319, contributed to children’s performance on the transfer test beyond the consistent use of Mass theory. In addition, crystallized intelligence did not moderate the consistent use of Mass theory on children’s performance on the transfer test, *b* = 0.17, *p* = 0.082.

Furthermore, we compared the three intervention groups on the transfer test: Verbal group, *M* = 8.17, *SD* = 4.64; Material group, *M* = 8.89, *SD* = 4.19; Free play group, *M* = 8.57, *SD* = 4.25. Crystallized intelligence did not moderate the effect of the intervention group on the children’s performance on the transfer test. An ANOVA showed no differences between the groups on performance in the transfer test, *F*(2) = 0.27, *p* = 0.762. Furthermore, there were no differences between the groups if the consistency at T1 was included, *F*(5) = 1.37, *p* = 0.242. Crystallized intelligence did not moderate the effect of consistency at T1^∗^intervention group on performance in the transfer test.

## Discussion

Science learning in early childhood can and should be promoted (Eshach and Fried, 2005; Trundle and Saçkes, 2012). However, studies on early science learning are quite sparse, and it remains unclear how to best support young children with different individual prerequisites.

Therefore, we conducted a study on 5- to 6-year-olds’ science learning in the specific domain of statics with regard to their prior intuitive theories and their individual cognitive prerequisites to investigate the effects of different types of play on theory adjustments. First, we were interested whether children explained their reasoning with Mass theory. In accordance with Pine et al. (2007), the children in our study faced problems estimating the stability of asymmetrical constructions and explaining the reasons for these stabilities. Most children provided another explanation and referred to characteristics of building blocks that have nothing to do with the mass or the center. Some children considered the center, and few considered the mass. This is in line with findings from other studies concerning the development of children’s knowledge of mass (e.g., Siegler, 1976; Siegler and Chen, 1998; Krist, 2010; Bonawitz et al., 2012; Weber and Leuchter, 2020).

The result that young children do not have a Mass theory, but rather a Center theory or provide answers unconcerned with mass and center can serve as a starting point for designing learning environments that foster children’s scientific reasoning, i.e., their theory adjustment, by providing them with perfect evidence for Mass theory (cf. Koerber et al., 2005; Klahr et al., 2011; Gopnik and Wellman, 2012). These learning environments should consider developmentally appropriate practice, i.e., play with scaffolds (Copple and Bredenkamp, 2009; Weisberg et al., 2016). Thus, we investigated whether a playful intervention could support children’s theory adjustment from an intuitive prior theory to a Mass theory. Play was implemented in the form of construction play with building blocks with differing amounts of adult guidance. The Free play group played with blocks on their own. The Material group received static material scaffolds prepared by an adult. In both of these groups, the adult only motivated and praised the children’s efforts but did not intervene in the play process. The Verbal group received the same material scaffolds as the Material group, and additionally, an adult used verbal scaffolds during the play. Thus, the Verbal group received the highest amount of adult guidance. After the playful intervention as well as after the follow-up test, the Kaplan-Meier analysis showed that the children in the Verbal group were most likely to use Mass theory consistently to explain their reasoning. The Material group was more likely to use Mass theory than the Free play group. Group comparisons with a Cox regression showed that the Verbal group outperformed the Free play group but not the Material group.

The acquisition of Mass theory however, might be dependent on interindividual variables such as intelligence and consistency of prior theory that interact with the degree of scaffolding. Thus, we investigated whether children’s prior theories are related to the adjustments of their theories to Mass theory. The link of children’s prior theories with theory adjustment to Mass theory seems to be partly dependent on intervention type. The children in the Verbal group who had answered inconsistently during the pretest were most likely to adopt a Mass theory after the playful intervention compared to all other groups.

Last, we investigated whether the use of Mass theory to explain stabilities was related to children’s results on a paper-pencil transfer test. Mass theorists performed better on the transfer test than Center theorists and children in the Other category. Our findings contribute to the literature on science education in the kindergarten years and will be discussed following the order of the research questions.

### Children’s Use of Mass Theory

The first research question was concerned with children’s explanations of asymmetrical constructions’ stabilities before the playful intervention. We investigated whether 5- to 6-year-old children explained asymmetrical constructions’ stabilities with Mass theory, Center theory or Other.

Considering the assumptions of theory theory (Gopnik and Wellman, 2012), we found that children’s theories about stability encompassed causal relations, as the theories provided explanations for the stability of the constructions and allowed for predictions of whether a construction would be stable or unstable depending on how it is supported. Thus, a child with a Center theory believes that the middle of a construction needs to be supported and that the middle is the cause for a construction’s stability. A child with a Mass theory believes that a construction’s weight needs to be supported and that the weight distribution is the cause for a construction’s stability. A child providing another explanation might have other ideas concerning the causal relationship between support and stability, e.g., the color. Since most children could not explain asymmetrical constructions’ stabilities correctly, we can assume that children do not have a Mass theory (e.g., Siegler, 1976; Siegler and Chen, 1998; Krist, 2010; Bonawitz et al., 2012; Weber and Leuchter, 2020).

Nevertheless, the children were more likely to use Center theory and less likely to provide another explanation in the course of the pretest. The reason might be that they received visual confirmation of the Center theory for the symmetrical items. Hence, the children might have inferred and generalized Center theory as an explanation for constructions’ stabilities (Bonawitz et al., 2012). Therefore, the children might have acquired a Center theory instead of remaining in the Other category or kept their Center theory instead of adopting Mass theory.

Even though all the children had the opportunity to learn about the mass even during the pretest because they received feedback concerning the constructions’ stabilities, the probability that the children would explain their reasoning with Mass theory remained the same across all three items at pretest. This outcome indicates that the presented evidence at pretest might not have been sufficient to acquire Mass theory. Even though the children observed perfect evidence for Mass theory (cf. Koerber et al., 2005), a short presentation and asking for explanations about the stability in a one-to-one setting was not enough to introduce the children to Mass theory. Since the children seemed to be unable to acquire an understanding of the mass that easily, construction play with varying degrees of structuring seems to be an appropriate approach to investigate whether the children could acquire an understanding of the mass during a play.

### Effects of Guided Play

The second research question was concerned with the playful interventions’ effects on children’s consistent use of Mass theory and the possible relationship with intelligence.

From the results it can be concluded that the more support the children received when confronted with evidence, the more likely they were to adjust their Other or Center theory and to explain their reasoning with Mass theory. This result indicates that children need support when learning about stabilities. Guided play with material and verbal scaffolds has been shown to support children’s acquisition of Mass theory more than free play (cf. Zosh et al., 2018). In the Material group, the children might have overlooked the evidence, and in the Free play group, the children could only have observed it randomly.

Consistent with the literature on theory theory and theory-evidence coordination (e.g., Koerber et al., 2005; Gopnik and Wellman, 2012), our results indicate that three factors should be fulfilled when supporting young children’s learning about science: (1) perfect covariation of the evidence, (2) considering the children’s prior theories, and (3) leaving the children with enough time to explore the evidence. We approached these factors by (1) taking care to present children with perfect covariation. We only used constructions that included asymmetrical features that always confirmed Mass theory but always disconfirmed Center theory. (2) We assessed children’s prior theories at pretest and tried to confront them with evidence supporting Mass theory and contradicting their Center theory. (3) The children were free to play with the provided materials for an hour so that they had a sufficient amount of time to explore and play with the materials. Furthermore, we considered the children’s developmental constraints and related science to their everyday activities by using different playful activities with building blocks as a learning setting (Copple and Bredenkamp, 2009).

The activities the children engaged in fulfilled the characteristics of play (Rubin et al., 1983; Pellegrini, 2013; Weisberg et al., 2013; Trawick-Smith, 2015; Daubert et al., 2018). The children played voluntarily, and the play was child-directed and contained elements of choice. We did not measure the children’s motivation during the play. Therefore, we cannot make a statement about their intrinsic motivation. An indication of their motivation might be that the children could stop playing at any time, but approximately 95% of the children continued to play for the provided time in all groups, as the video recordings for the manipulation check and the experimenters’ written records showed. Furthermore, highly inferential analyses of the recordings demonstrated that all of the children in all of the groups showed playful behavior (cf. Bundy et al., 2001). The play was not free of goals because we had a specific learning goal, namely, the acquisition of Mass theory, in mind. However, the play was still process-oriented, as we did not push this goal on the children.

Our playful intervention was based on the continual view postulated by, e.g., Zosh et al. (2018). The free play was free of an adult’s guidance, the guided play with material scaffolds was structured and offered children suggestions for playing with blocks, and the guided play with material and verbal scaffolds offered additional verbal guidance. Specifically, when implementing the verbal scaffolds, we *asked for the children’s prior knowledge* to allow them to express their presumptions to facilitate the adjustment of their theory (Mayer, 1997; Weinert and Helmke, 1998; Gurlitt and Renkl, 2010). By *asking for reasoning*, the children could be made aware of their theory, and they were supported in structuring their theory (Hsin and Wu, 2011). The *provision of explanations* helped the children organize new knowledge, e.g., knowledge about the mass, and integrate this new knowledge into their theories (Richey and Nokes-Malach, 2013). By *encouraging comparisons*, we tried to support the children in comparing stable and unstable constructions and to generalize the underlying principle, i.e., the mass (Hsin and Wu, 2011; Richey and Nokes-Malach, 2013). Last, *modeling* might have offered the children the possibility for imitation (Mayer, 2004; Hmelo-Silver et al., 2007).

Our study showed that crystallized intelligence had a positive relationship with children’s consistent application of Mass theory directly after the intervention. The interaction of crystallized intelligence with the intervention group showed that children with high crystallized intelligence profited more in the Verbal group than did children with high crystallized intelligence in the Material group. Fluid intelligence did not relate to the consistent explanation with Mass theory directly after the intervention. This outcome indicates that children acquired Mass theory regardless of their ability to mentally represent and abstract the spatial features of constructions’ stabilities. After including the follow-up, both fluid intelligence and crystallized intelligence related to the consistent explanation with Mass theory over an extended period of time. Crystallized intelligence interacted with the intervention group and related to the consistent application of Mass theory in the Verbal group and the Free play group but not in the Material group. Children with low crystallized intelligence were more likely to adjust their theories in the Material group, while children with high crystallized intelligence were more likely to adjust their theories in the Verbal group.

Language capacity is understood as an indicator of crystallized intelligence (Cattell, 1987; Flynn and Blair, 2013). Thus, our results suggest that the children with a low language capacity profited most from the Material group. Seemingly, the material scaffolds were sufficiently self-explaining so that the children with a low language capacity could observe evidence for Mass theory and adjust their theories. Moreover, the children with a low language capacity in the Verbal group did not profit from the intervention because they may have suffered from a high cognitive load (Kirschner, 2002). They not only needed to process the new information about the learning content provided through the verbal scaffolds but also the language itself. In contrast, the children with a high language capacity profited from the verbal scaffolds that were provided in the Verbal group. Our findings imply that when providing verbal instructions and support, it may be important to consider children’s language capacity.

Children with a higher fluid intelligence, i.e., a capacity to represent constructions mentally and abstract important spatial features (Cattell, 1987; Weiß and Osterland, 2013; Weber and Leuchter, 2020), were more likely to acquire Mass theory over the course of 10 weeks. This outcome is in line with studies from developmental psychology showing that children’s Mass theory develops between ages five and seven (Bonawitz et al., 2012). In their everyday lives, children have many possibilities to explore stabilities and develop an understanding of the underlying principles. A possible explanation might be that children with a higher fluid intelligence learn these principles faster than children with a lower fluid intelligence (cf. Weber and Leuchter, 2020).

In addition to intelligence, other individual competencies, such as children’s prior theories about stabilities, could relate to children’s acquisition of Mass theory.

### Relationship of Children’s Prior Theories and Their Consistent Use of Mass Theory

The third research question was concerned with the role of children’s prior theories on their consistent use of Mass theory after the interventions.

The children with inconsistent prior theories who received the highest amount of support (Verbal group) acquired a Mass theory, while those children who received less support (Material group and Free play group) did not acquire a Mass theory. This result indicates that prior theories play a role in theory adjustment, which is in line with findings concerned with Bayesian inference in the context of theory-theory by, e.g., Bonawitz et al. (2012) and Sobel et al. (2004). The children who answered inconsistently at T1 did not have a consistent prior theory and therefore had the highest chance of acquiring a Mass theory. Their theoretical assumptions were inconsistent compared to the theories of children who had explained their reasoning consistently with Center theory or Other (cf. Koerber et al., 2005; Griffiths et al., 2011; Gopnik and Wellman, 2012; Gopnik, 2013). Since the children in the Verbal group were most likely to acquire a Mass theory, children seem to profit from high amounts of guidance and support; moreover, in our study, only observation seems to be insufficient for the understanding of counterevidence. In sum, children with inconsistent prior theoretical assumptions profit from supported play but do not adjust their theories by playing with blocks freely.

### Transfer Test

The fourth research question was concerned with children’s performance on a transfer test at follow-up. Independent of intervention group, we compared children who had used Mass theory consistently after the playful intervention to children who had failed to do so on a transfer test with asymmetrical block constructions. The children who had answered consistently outperformed children who had answered inconsistently. This result indicates that children who explain their reasoning with Mass theory are also more likely to rate asymmetrical constructions’ stabilities correctly, which suggests that the children acquired an understanding of Mass theory.

However, comparing the three intervention groups, children performed equally well on the transfer test, even though we found group differences on the reasoning test. The transfer test, unlike the reasoning test, was a paper-pencil test and according to Karmiloff-Smith (1992) and Pine and Messer (2003) rather tested knowledge that children might not have been able to verbalize. The transfer test indicates that children in all groups had knowledge about stabilities at T3, but only the children in the Verbal group were able to verbalize their reasoning.

The children who had a low language capacity succeeded in the transfer test but not in the reasoning task. They did not have to explain their reasoning in the transfer test; they were only required to decide about the constructions’ stabilities. Although we tried to consider a low language capacity in the reasoning task by counting specific gestures as indicators for Mass theory, e.g., pointing to the Mass, or Center theory, e.g., pointing at the middle, the transfer test was seemingly easier for the children to handle. This is especially meaningful for children with a different native language because these children might face challenges in explaining their reasoning adequately but might be able to show their knowledge about stability with a non-verbal test. Therefore, to offer children the opportunity to show their knowledge about science phenomena such as stability, methods that do not require the children to speak might be helpful.

### Limitations

There are some limitations to this study concerning the implementation and measurement.

#### Implementation of Play

Regarding the implementation of the playful intervention, we compared material scaffolds, material + verbal scaffolds and free play regarding their effects on children’s Mass theory. The effect of verbal scaffolds uncoupled from material scaffolds was not investigated. Future studies could implement a verbal scaffolds group by presenting children with the same unstructured building blocks a free play group receives and adding verbal scaffolds. Moreover, the implementation of a baseline group not receiving any intervention would allow investigating whether free play has an effect on children’s theory adjustment toward Mass theory compared to children’s development.

We videotaped only some of the playful interventions for a manipulation check; as some children or their parents denied permission to videotape, some interventions were only audio-recorded. Moreover, for a few interventions, neither videos nor recordings exist due to technical failures with the equipment. Therefore, children’s behavior during play cannot be analyzed, even though there might be interindividual differences in how children interacted with the experimenter and used the provided materials. For example, some children might have asked for help more often or might have built with the building blocks more actively, while others may have instead watched other children build. Furthermore, the materials provided in the guided play groups served as suggestions, and children in all groups were free to build other buildings. From the existing videos and recordings, we assume that the children in the guided play groups played the suggested activities and used the scaffolding materials. However, some children might have built at a higher pace and thus may have built more of the suggested structures than other children. Last, regarding children’s behavior, the amount of time that the children spent playing on their own or with other children, their manipulation of and their conversations about the building blocks might have contributed to children’s Mass theory after the intervention. These factors should be investigated in a future study.

In this study, we only used a limited set of verbal scaffolds and did not control for the verbal scaffolds’ adaptability. However, the adaptability might have contributed to children’s acquisition of Mass theory. Therefore, children’s and experimenters’ behavior during play should be investigated in the future.

#### Measures

The children received eight items showing different block constructions, and three asymmetrical items were used to assess children’s theories about stability. The other symmetrical items were used to familiarize the children with the test and motivate them during testing because children find it easier to estimate symmetrical constructions’ stabilities (Krist, 2010). These Center theory-compliant items might have led some children to adopt a Center theory instead of remaining in the Other category, even though the evidence for Center theory was imperfect. The results of this study show that although the children received these Center theory items, many still adopted Mass theory after the playful intervention. Future studies might benefit from the use of more items, which would also prolong the testing time, as more asymmetrical as well as symmetrical items would be needed. This addition could impact the children’s attention capacity and their motivation to participate.

Children received feedback about the constructions’ stabilities during testing because they built the construction and then removed the supporting black block to ascertain whether they had rated the stability correctly. Therefore, children had the opportunity to learn during testing, and the items were dependent on each other. As a result, we could not just sum up the items, and every item was considered a point in time. Thus, we used methods of risk-event analysis to analyze the data. Independent measurements would allow for different statistical approaches, e.g., statistical procedures that refer to the mean. Thus, in future studies, to achieve independent measures, children could not build constructions on their own but only rate and explain stabilities on the basis of photographs so that they do not receive feedback about stability.

Nevertheless, our study indicates that guided play can support young children’s science learning. Differing degrees of scaffolding in guided play can be beneficial for helping children with different prerequisites adjust their theories when observing new evidence.

## Data Availability Statement

The datasets generated for this study are available on request to the corresponding author.

## Ethics Statement

Ethical review and approval was not required for the study on human participants in accordance with the local legislation and institutional requirements. Written informed consent to participate in this study was provided by the participants’ legal guardian/next of kin.

## Author Contributions

AW and ML contributed to conception and design of the study. AW organized the database, performed the statistical analysis, and wrote the first draft of the manuscript. AW, TR, and ML wrote sections of the manuscript. All authors contributed to manuscript revision, read, and approved the submitted version.

## Conflict of Interest

The authors declare that the research was conducted in the absence of any commercial or financial relationships that could be construed as a potential conflict of interest.

## Footnotes

1 By Other, we do not imply that children have no theory at all, but rather treat it as a rest category for children’s answers that were neither concerned with the center nor the mass of constructions (cf. Bonawitz et al., 2012, p. 221).

2 Rounded percentages.
