# Supplementary material for: The Impact of a Construction Play on 5- to 6-Year-Old Children’s Reasoning About Stability
Source: Front Psychol. 2020 Jul 14;11:1737. doi: 10.3389/fpsyg.2020.01737 (PMC7372995; doi:10.3389/fpsyg.2020.01737)
Supplement: Supplementary file 1 [file Data_Sheet_1.pdf]

The impact of a construction play on 5- to 6-year-old children's reasoning about stability

Anke M. Weber, Timo Reuter & Miriam Leuchter

Supplementary Materials

Supplementary Material 1. Material scaffolds for all five activities played during the intervention in the Material group and the Verbal group.

Supplementary Material 2. Script for the verbal scaffolds used during the intervention in the Verbal group.

Supplementary Material 3. Example excerpts from the playful activities (translated from German).

Supplementary Material 4. Items of the reasoning test.

Supplementary Material 5. Items of the transfer test.

Supplementary Material 6. Percentages of correct answers on the reasoning test.

Supplementary Material 7. Results of the Kaplan-Meier analyses.

Supplementary Material 1. Material scaffolds for all five activities played during the intervention in the Material group and the Verbal group.

1. Black block:

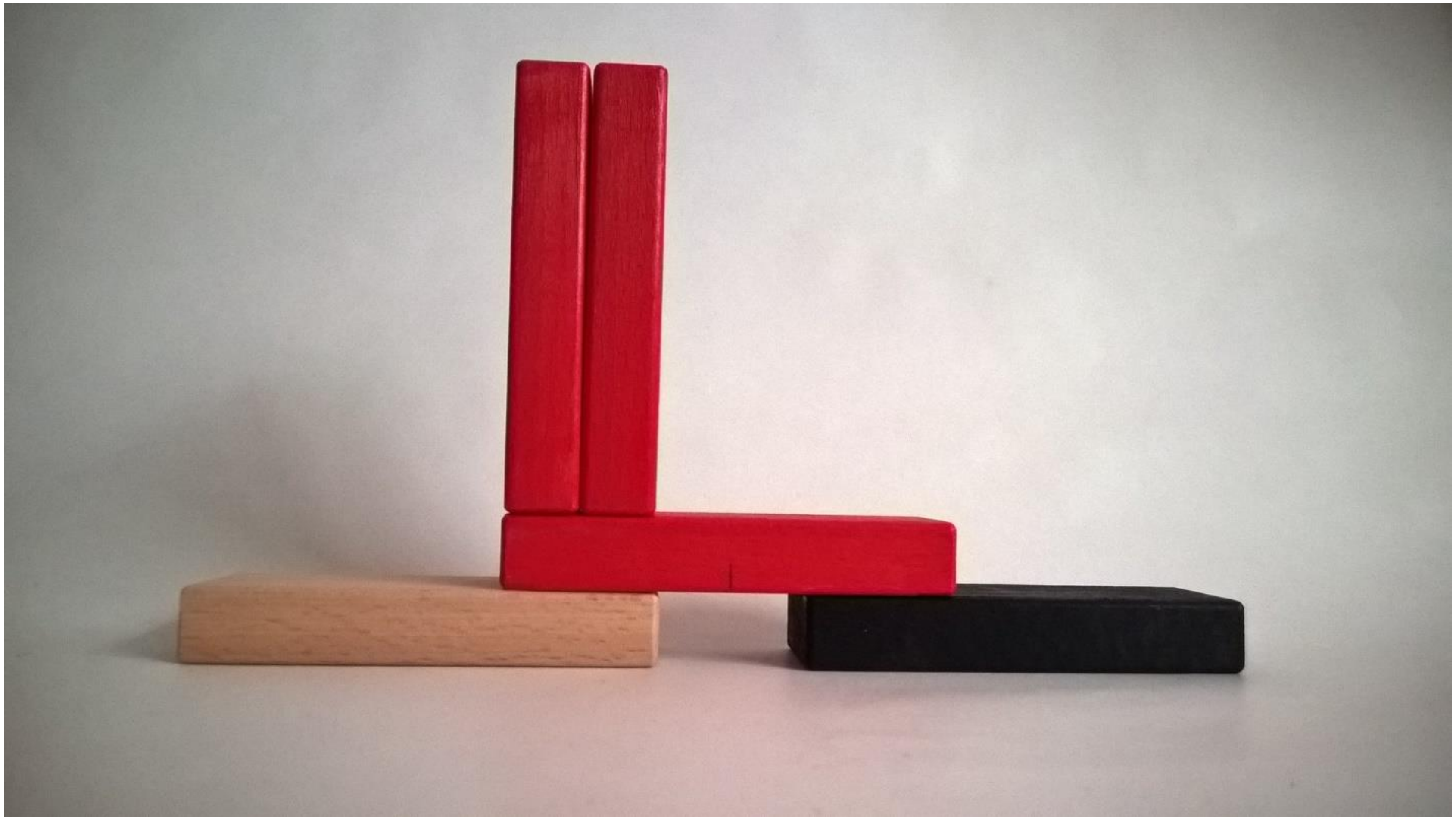

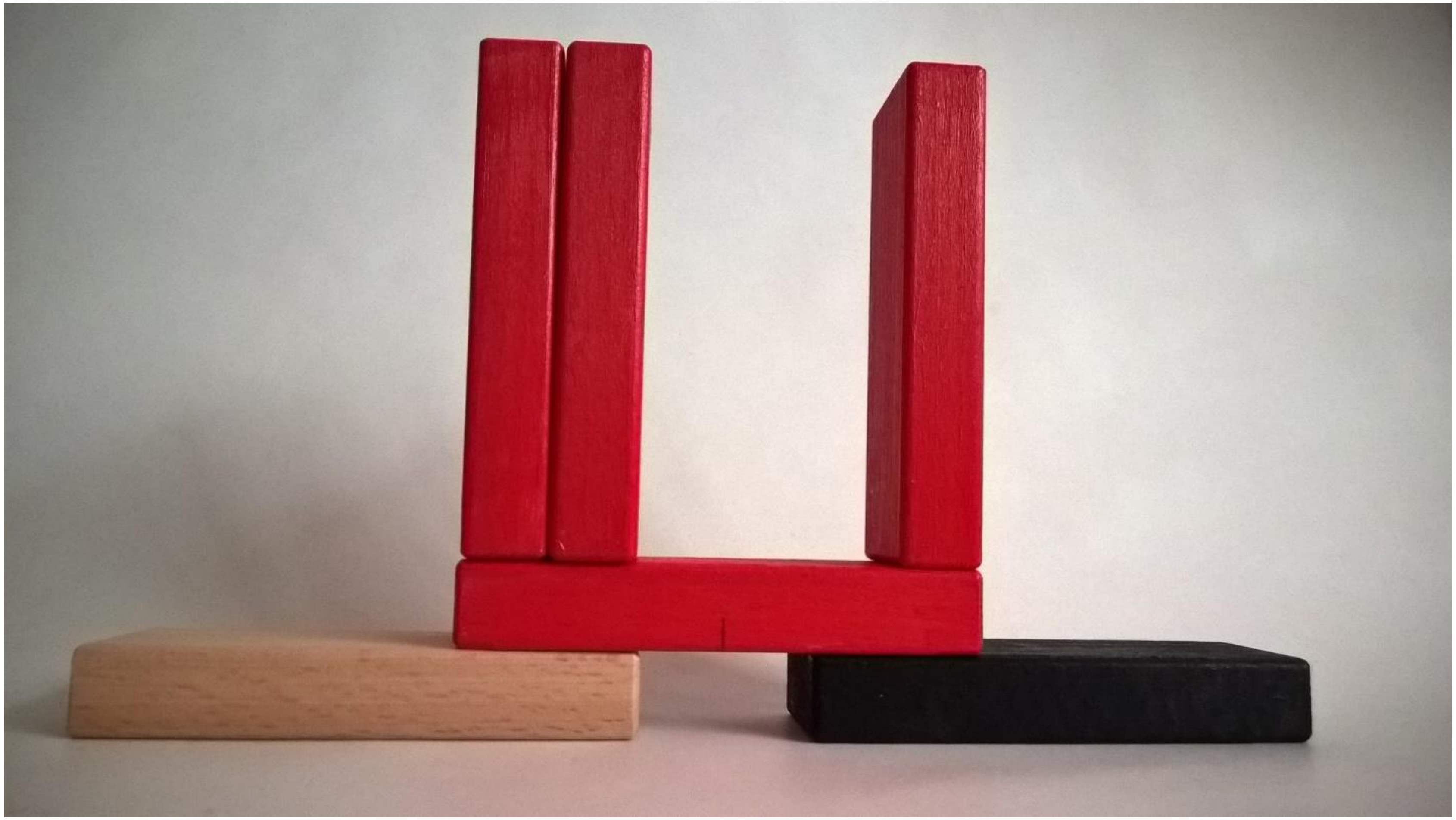

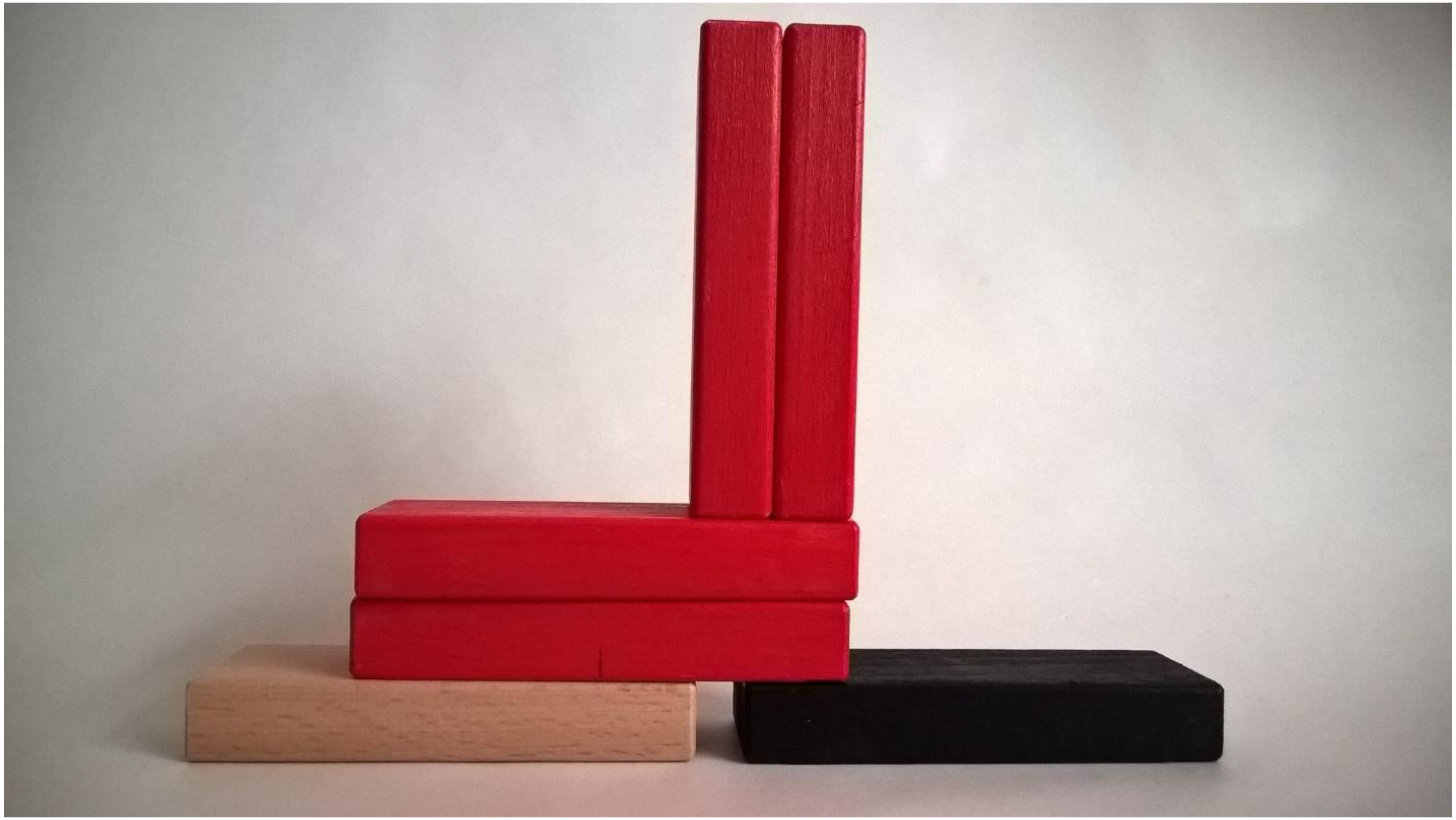

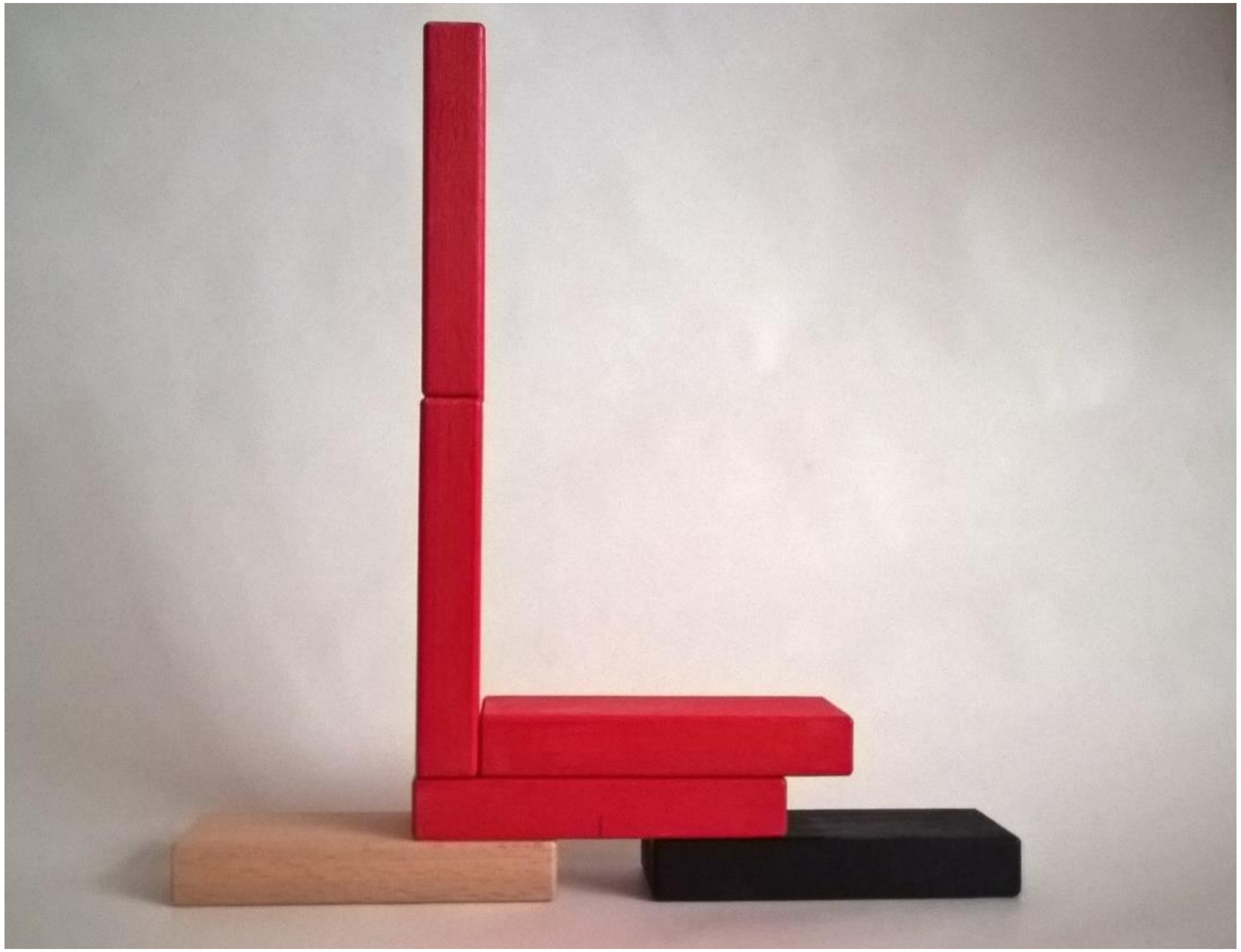

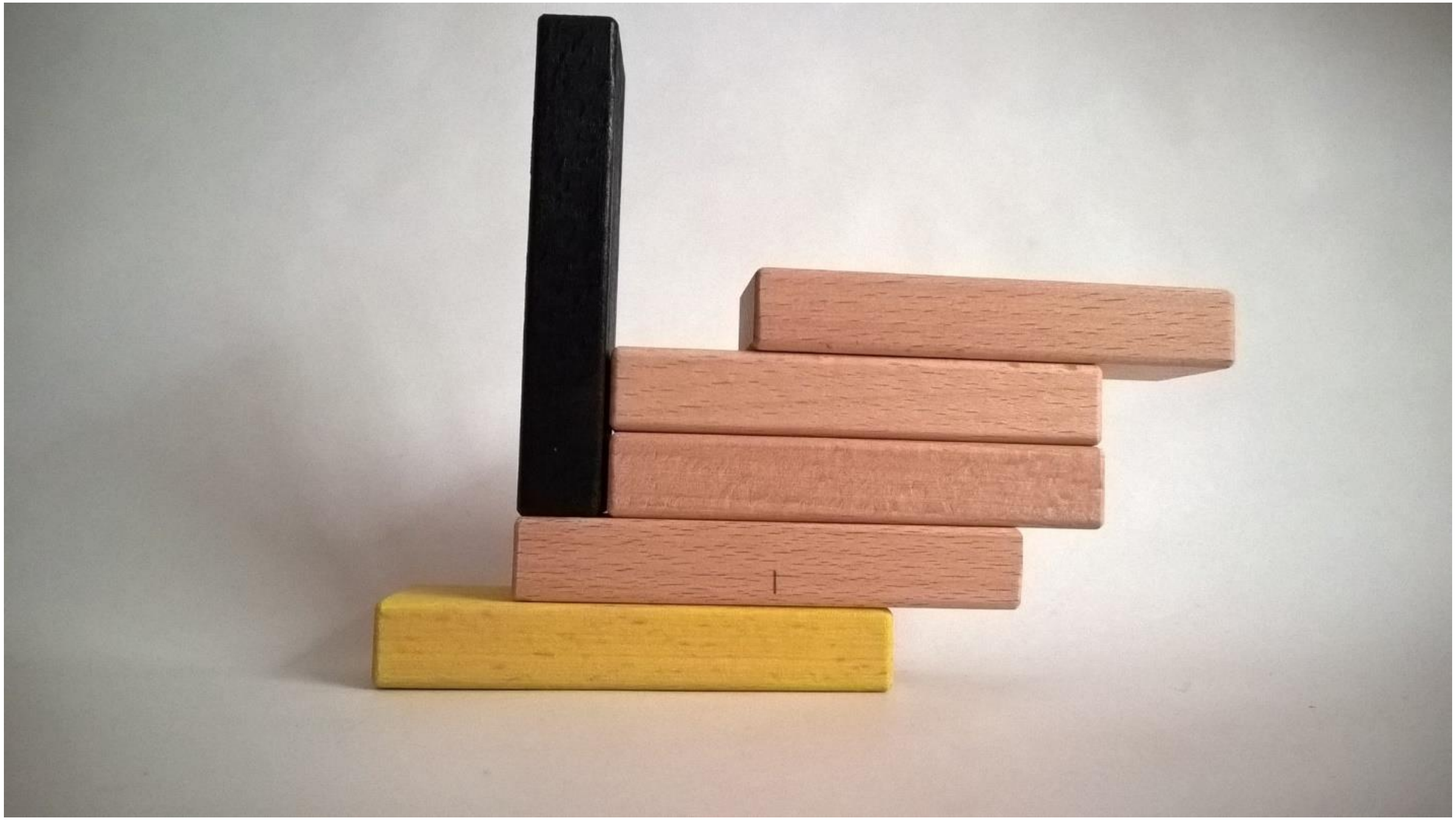

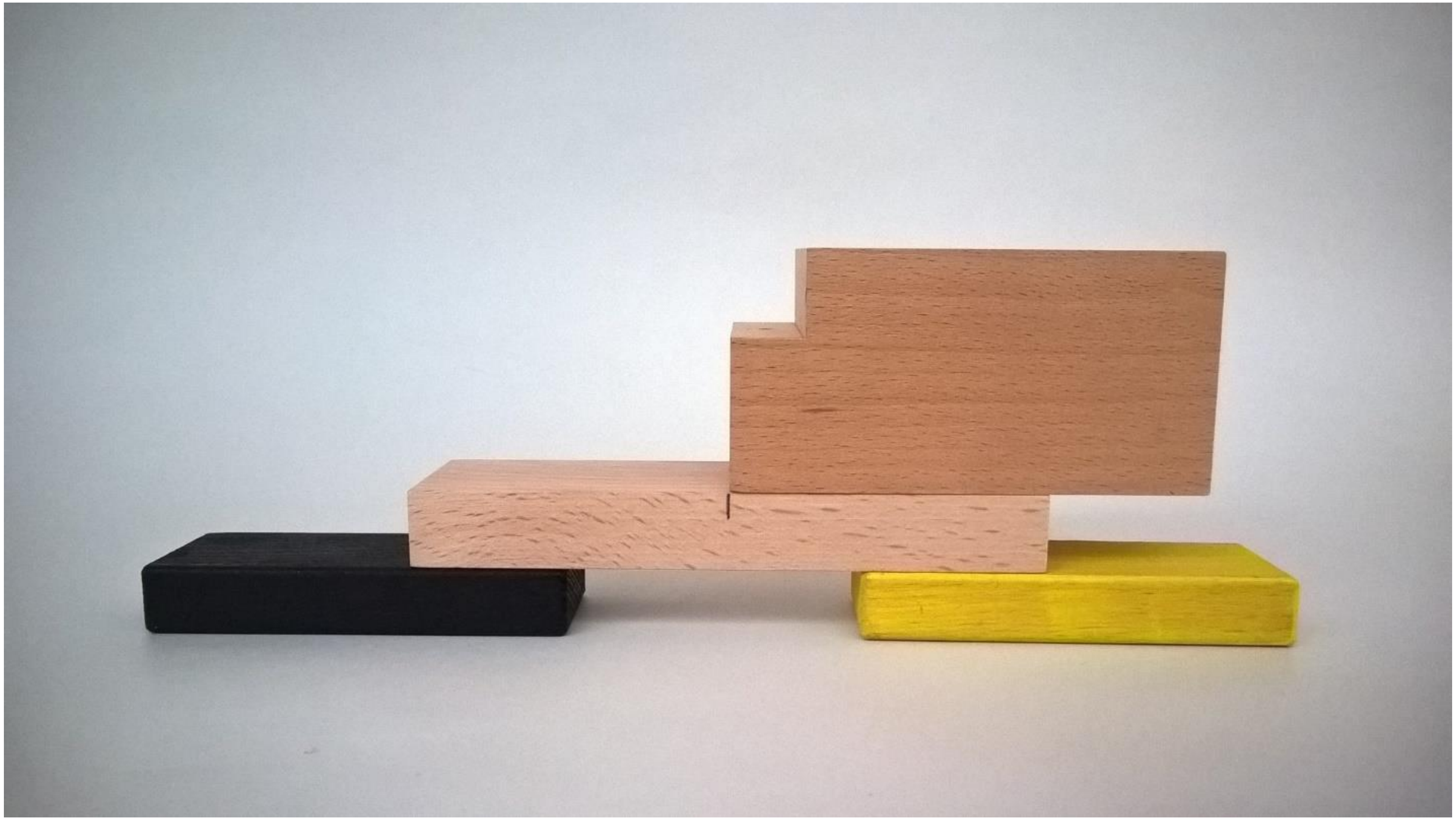

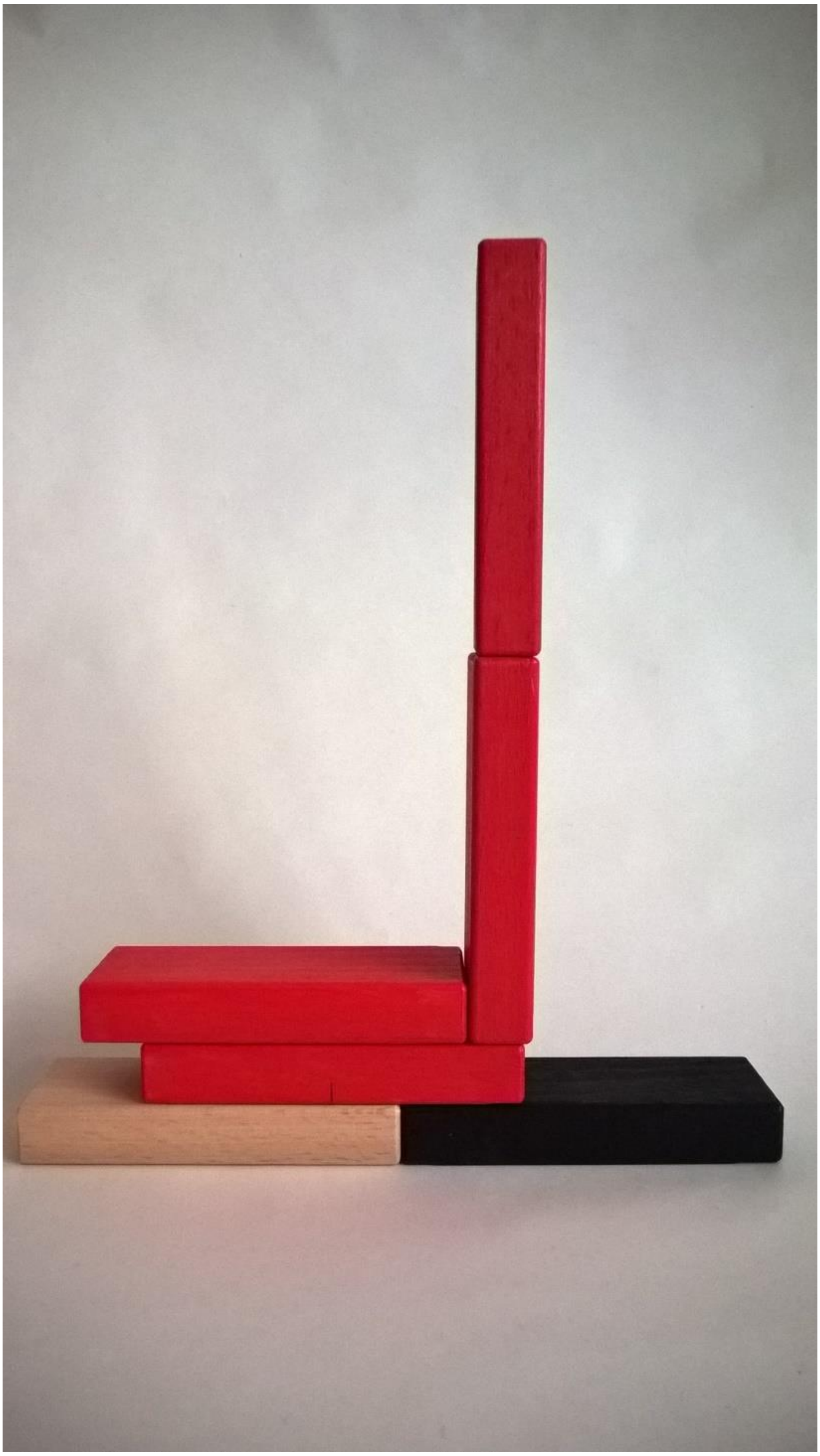

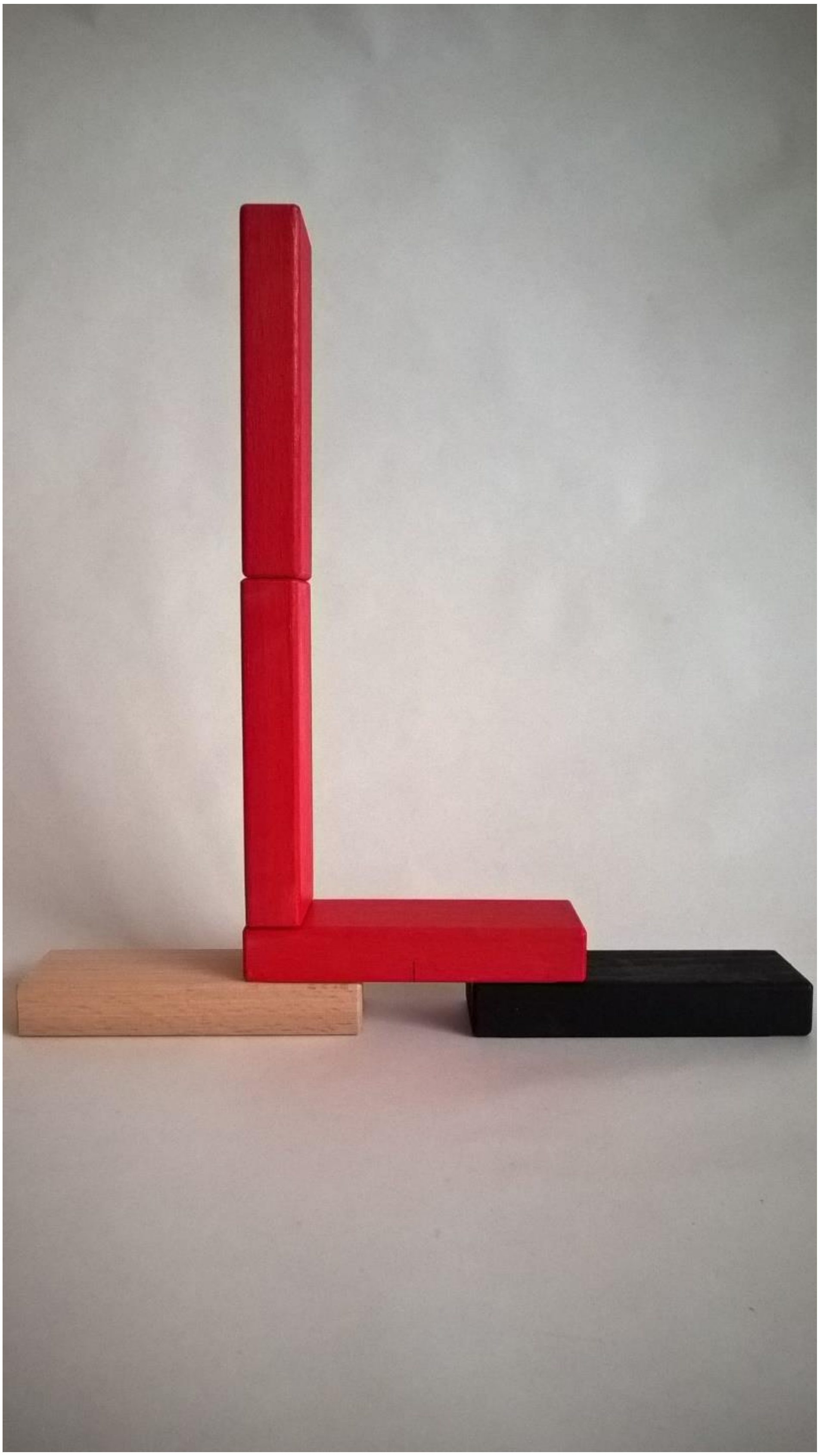

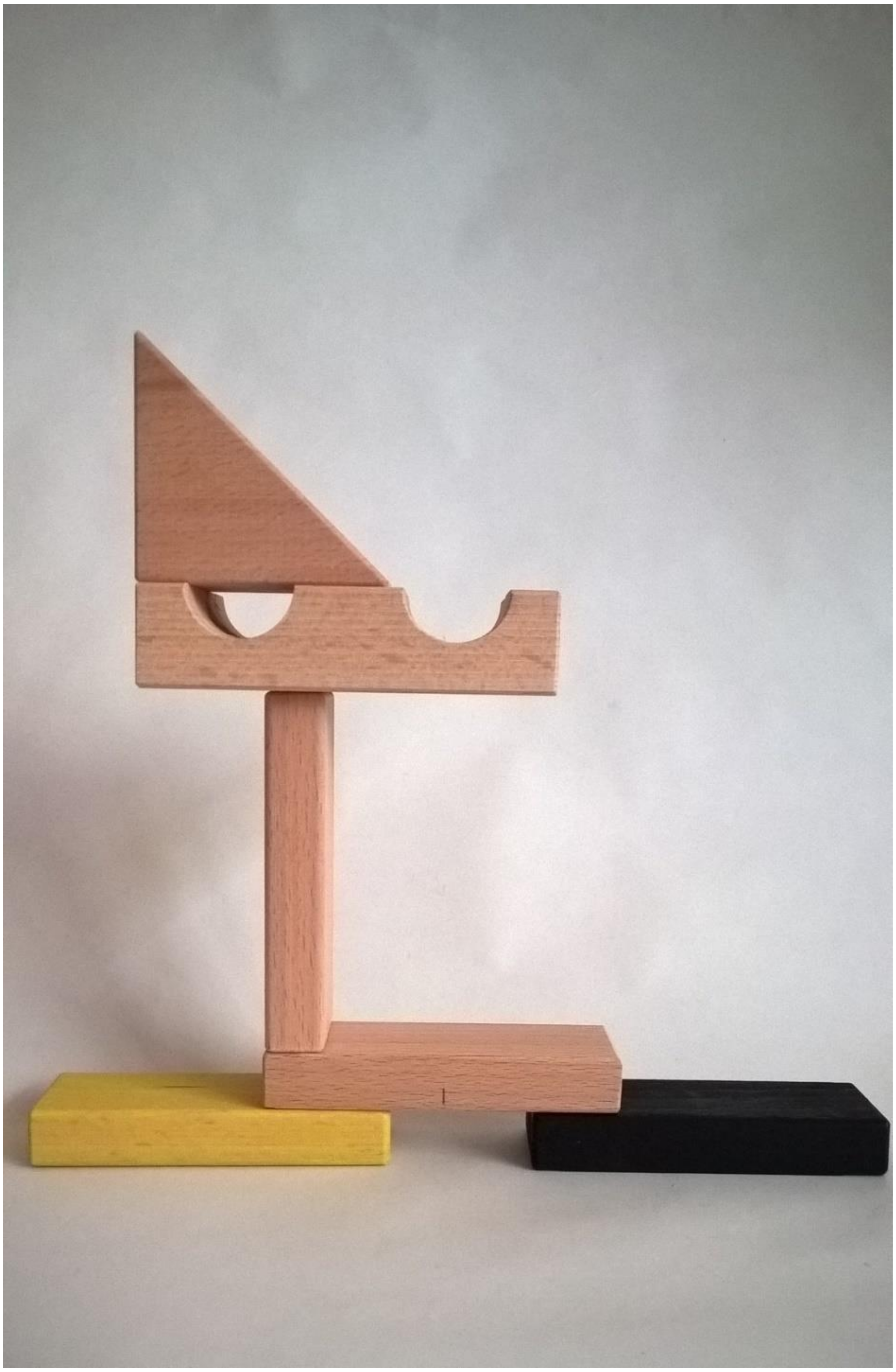

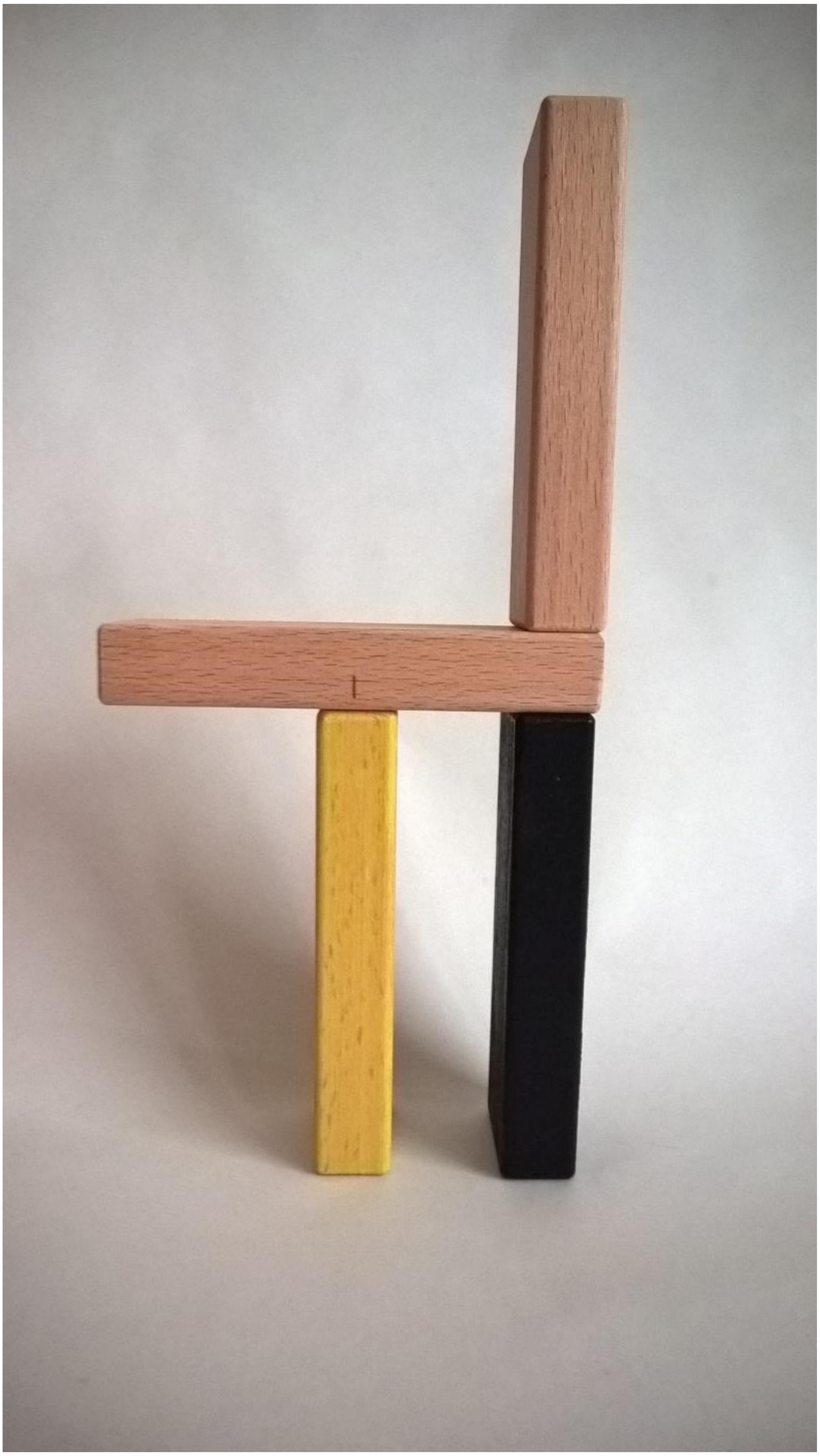

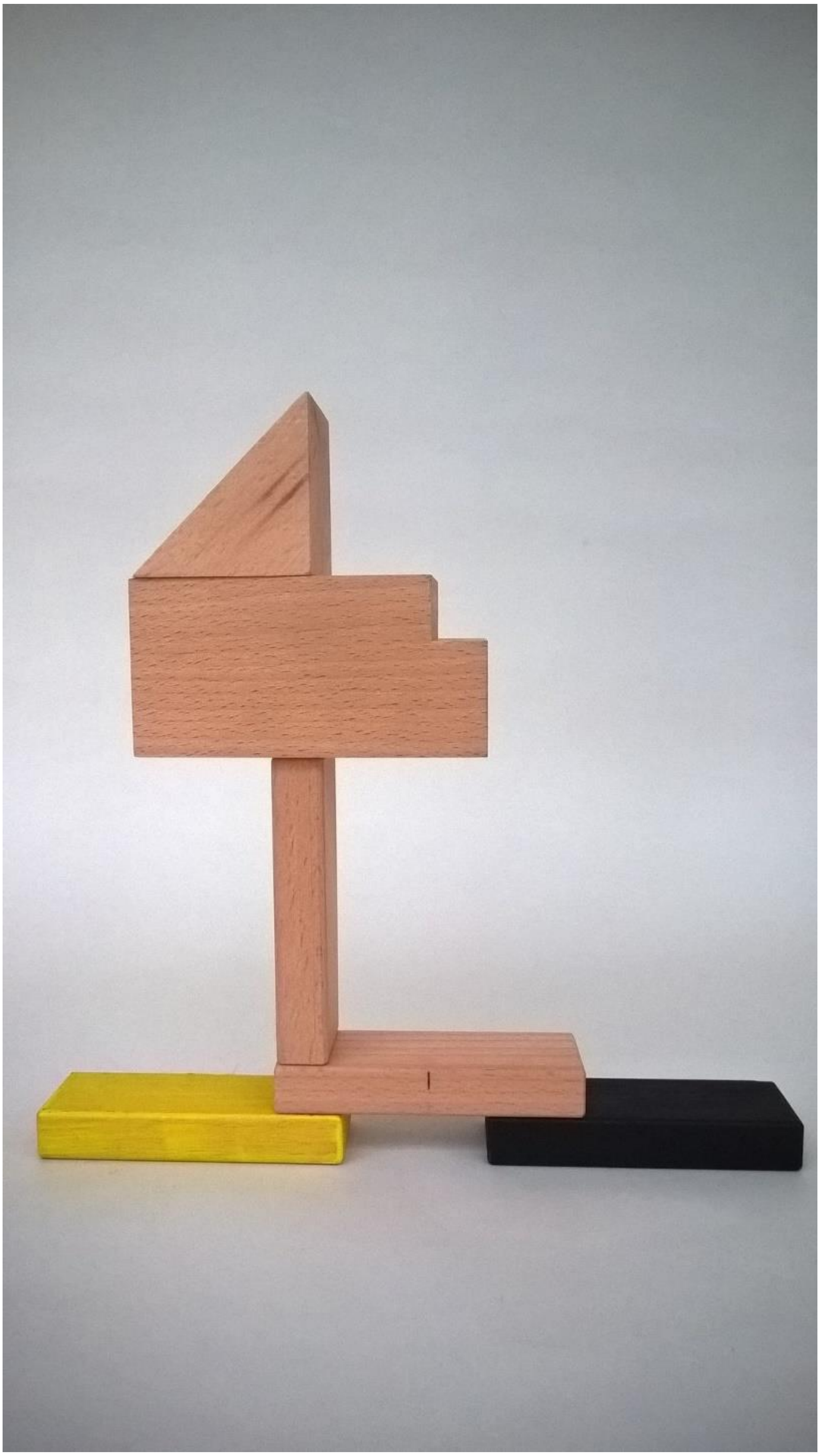

2. Add-a-block:

Picture children received:

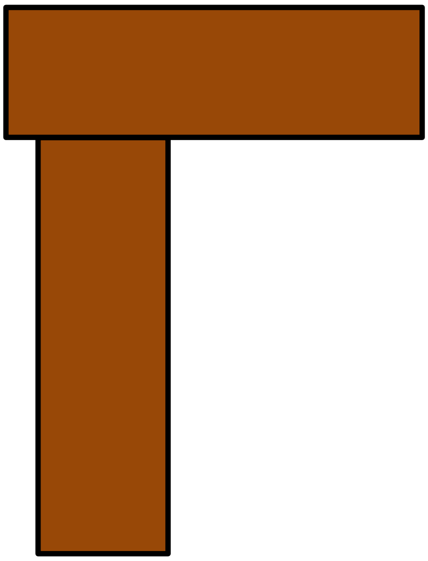

Possible solutions for the experimenters only:

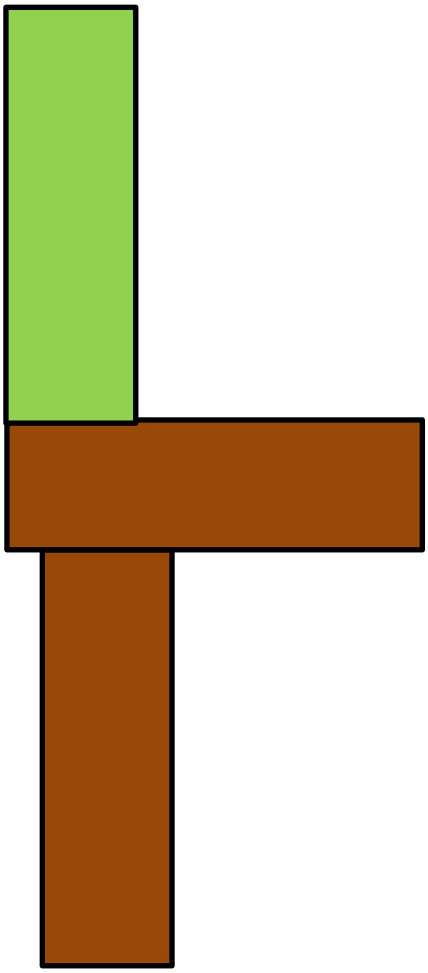

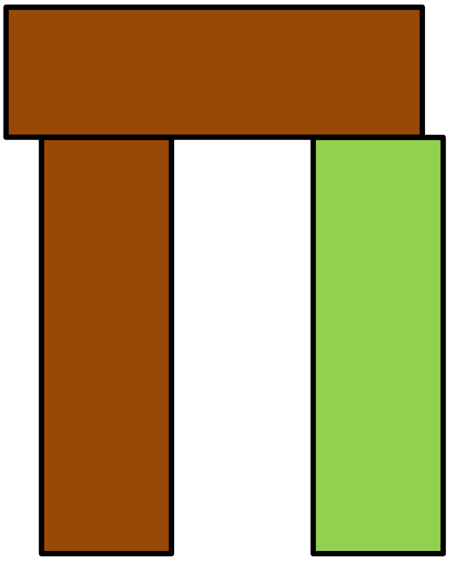

Picture children received:

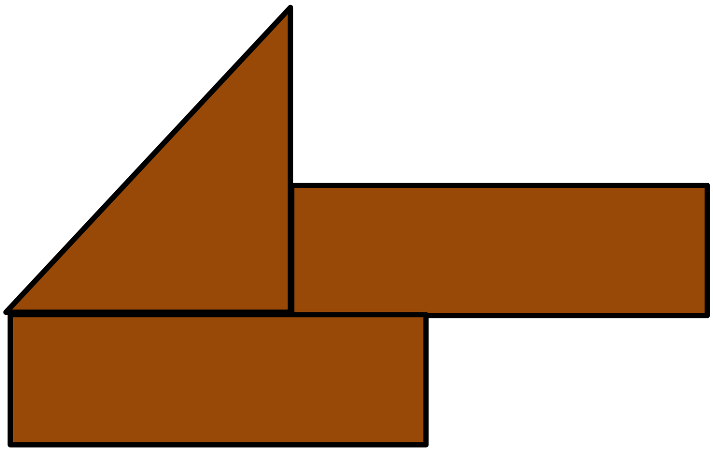

Possible solutions for the experimenters only:

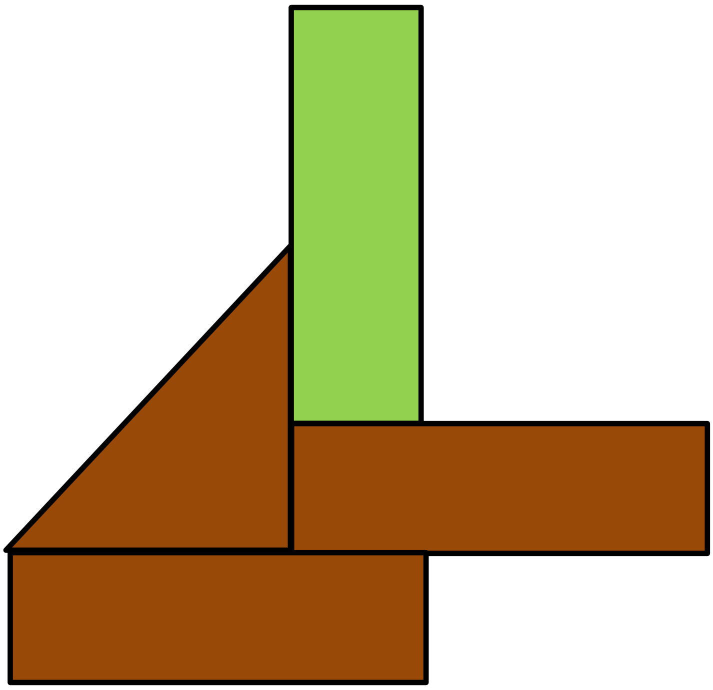

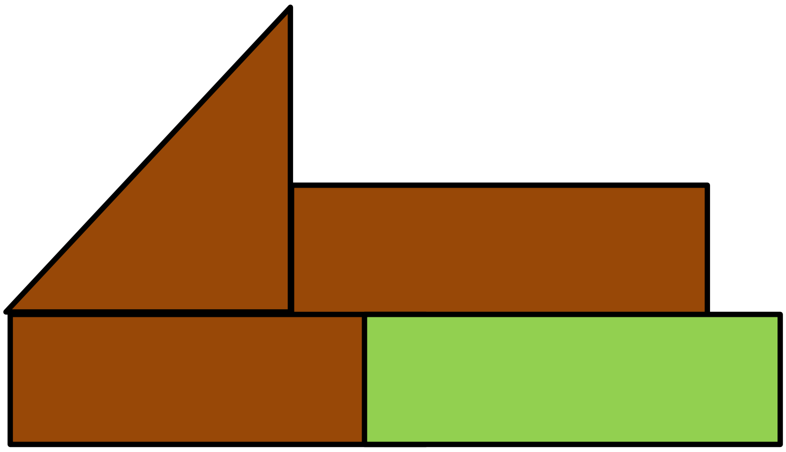

Picture children received:

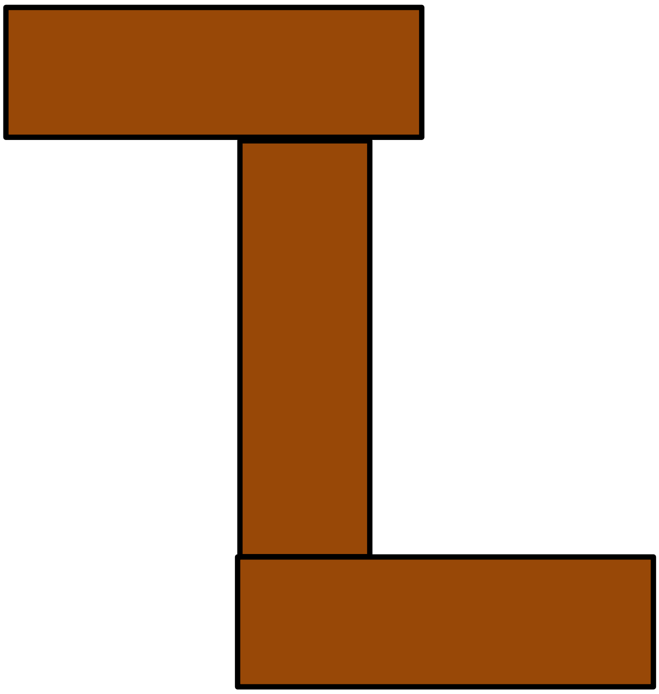

Possible solutions for the experimenters only:

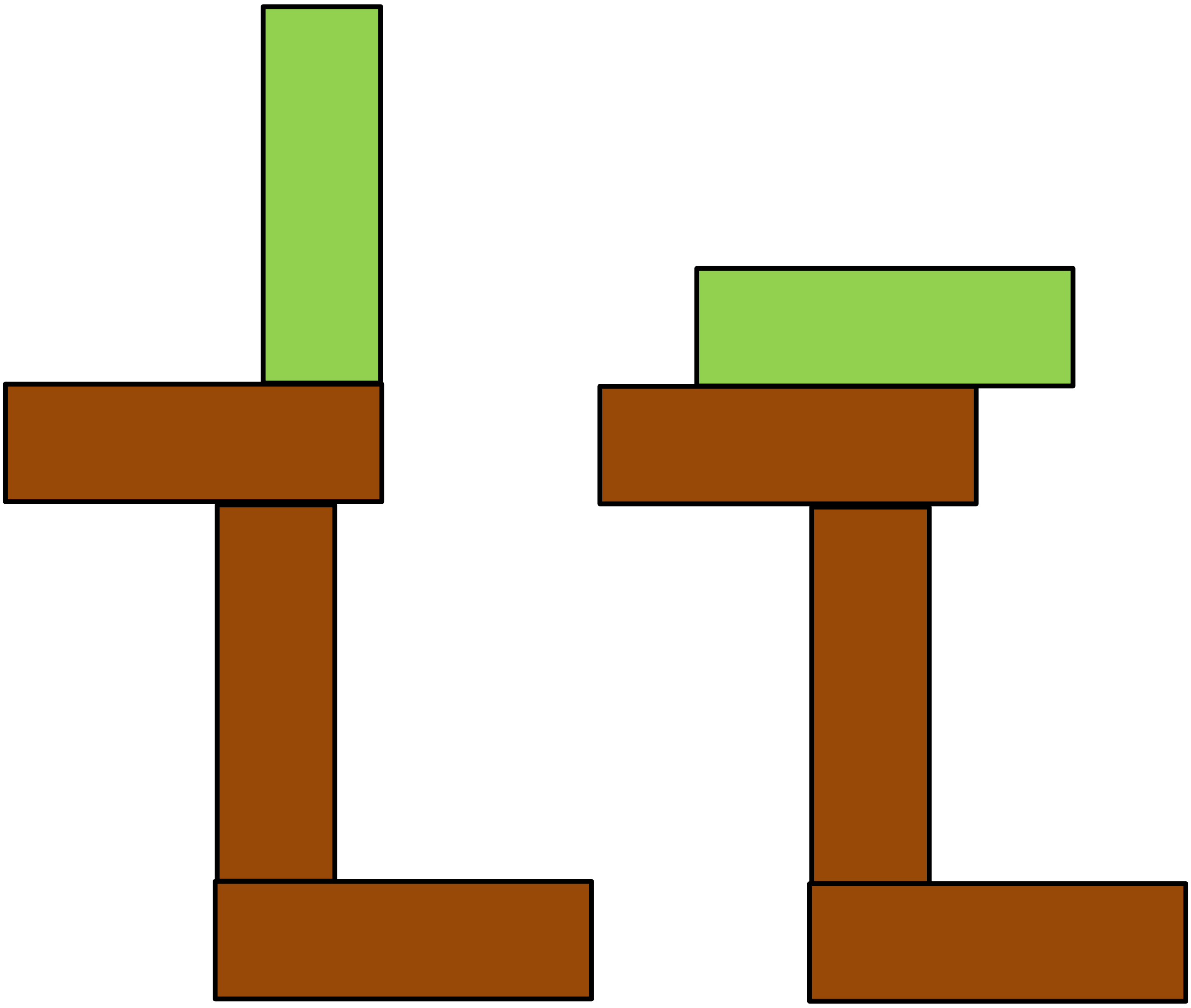

Picture children received:

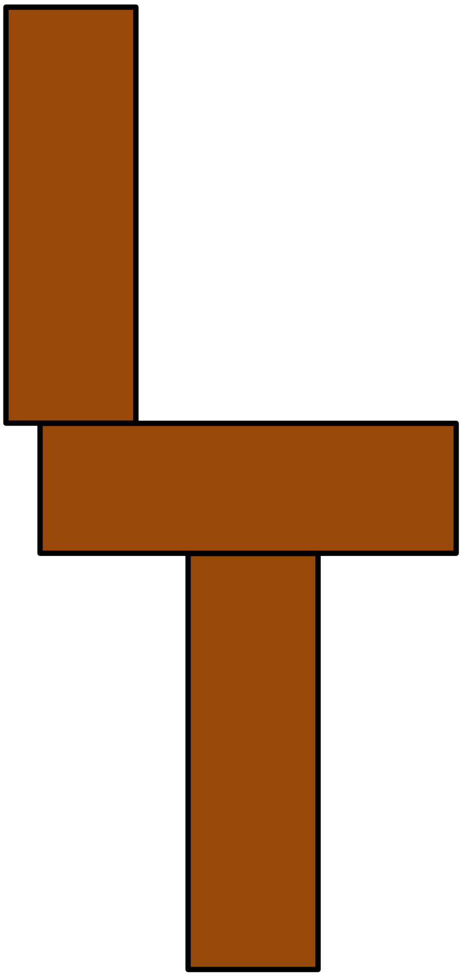

Possible solutions for the experimenters only:

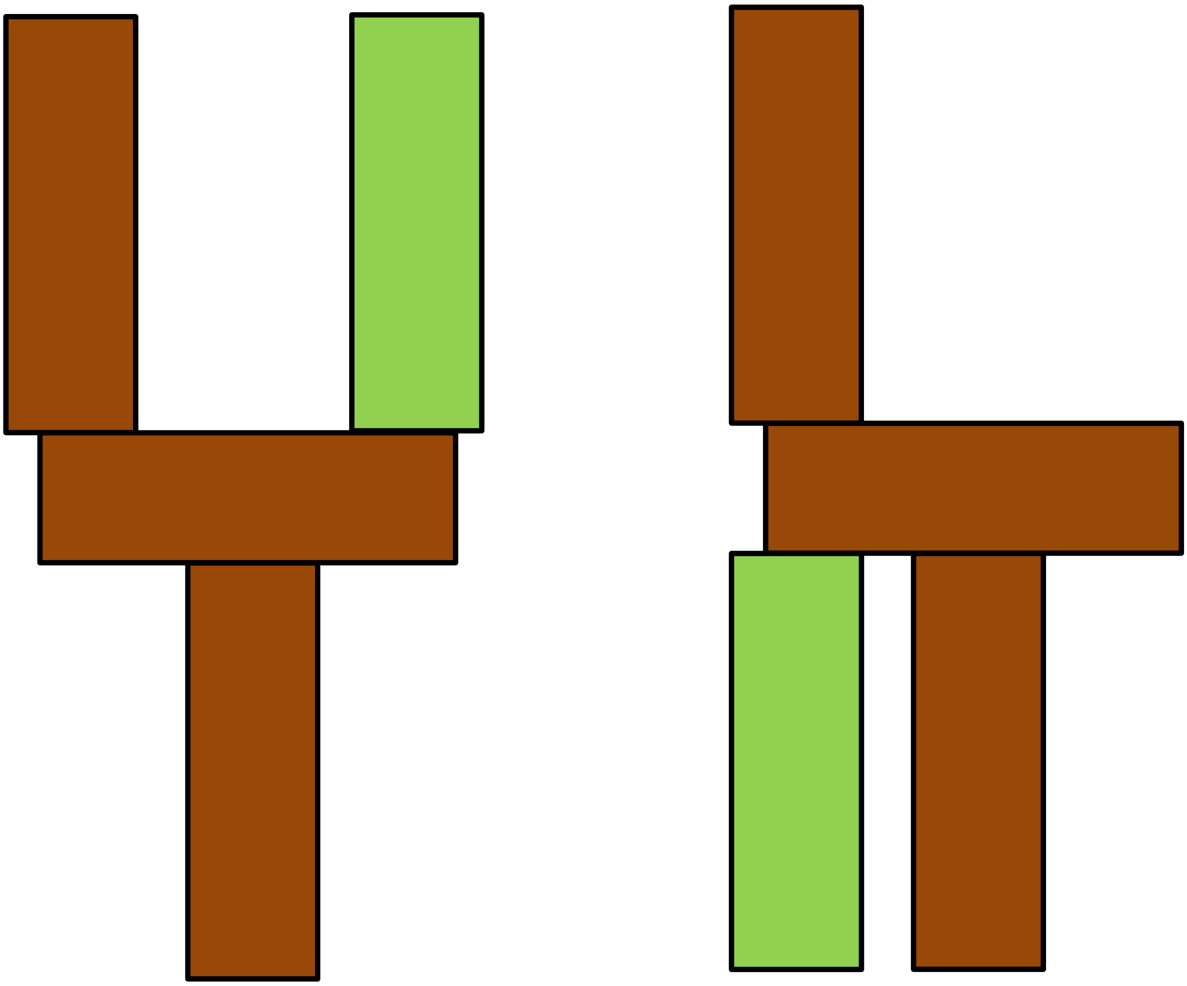

Picture children received:

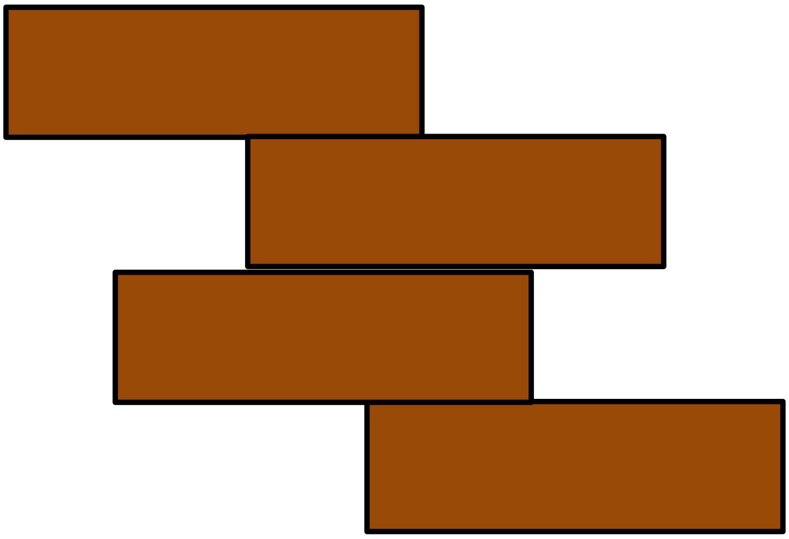

Possible solutions for the experimenters only:

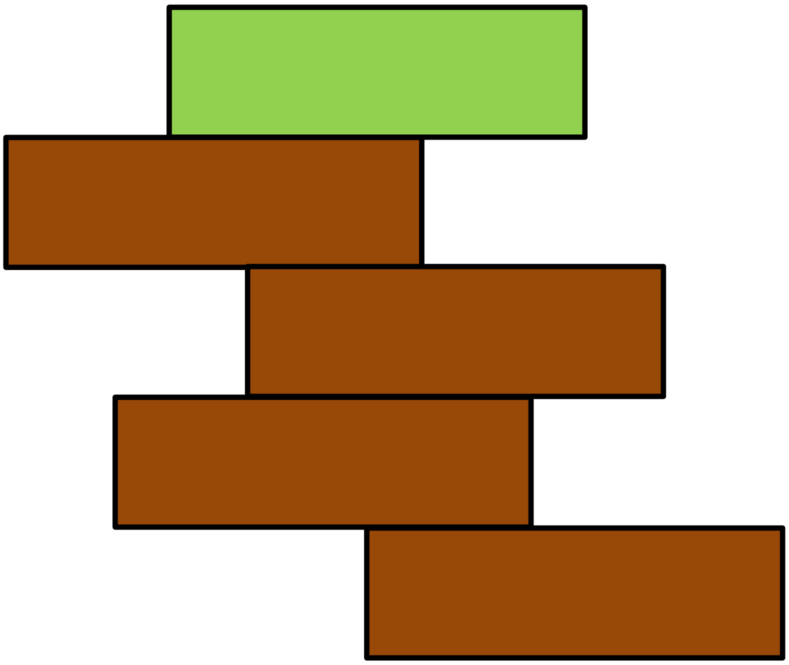

Picture children received:

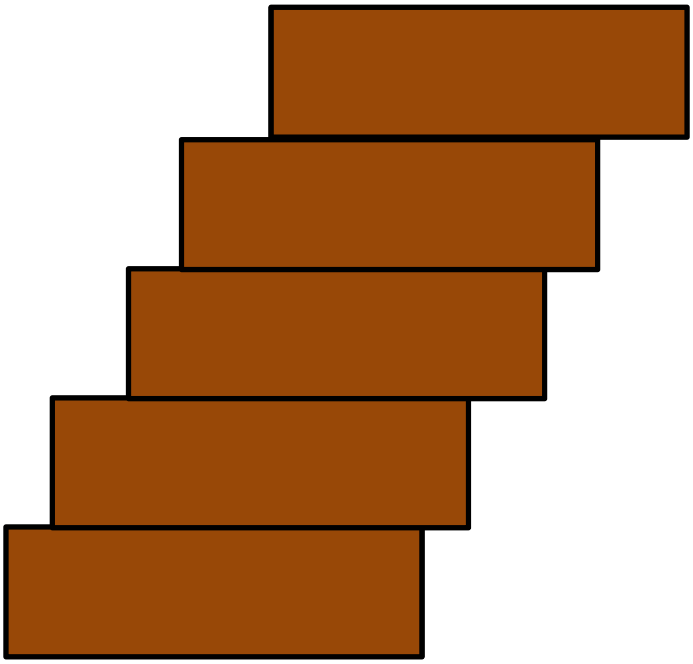

Possible solutions for the experimenters only:

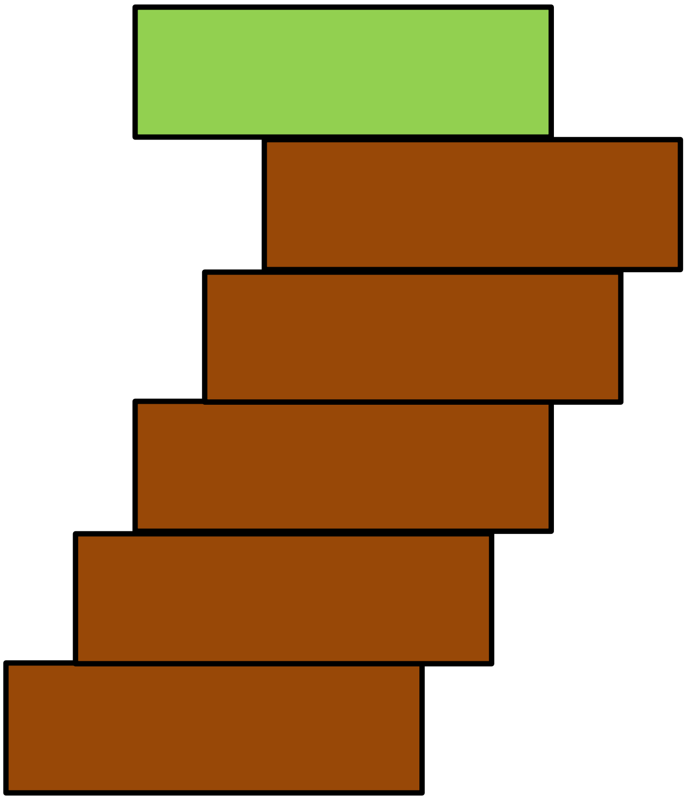

Picture children received:

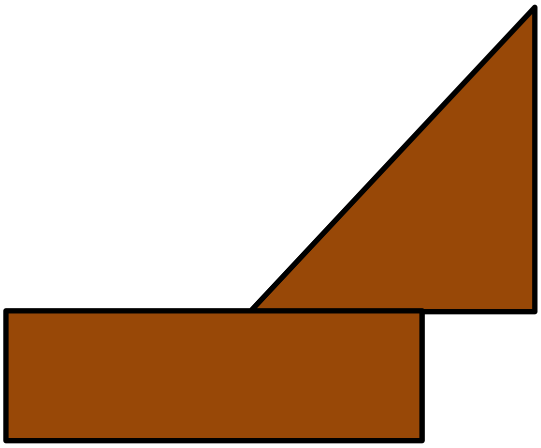

Possible solutions for the experimenters only:

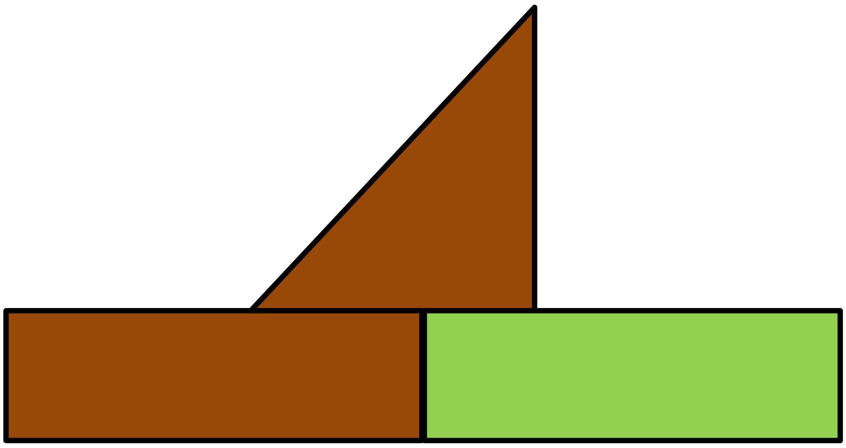

Picture children received:

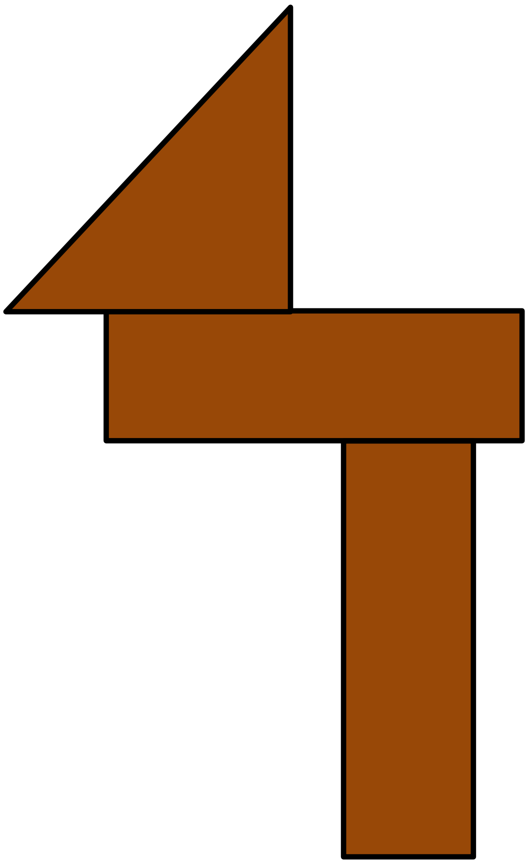

Possible solutions for the experimenters only:

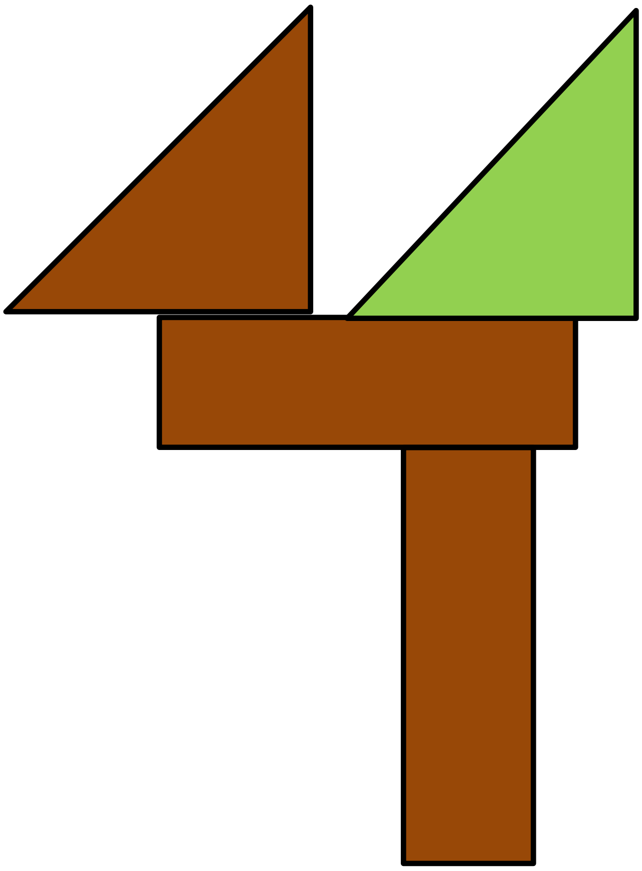

3.Sliding

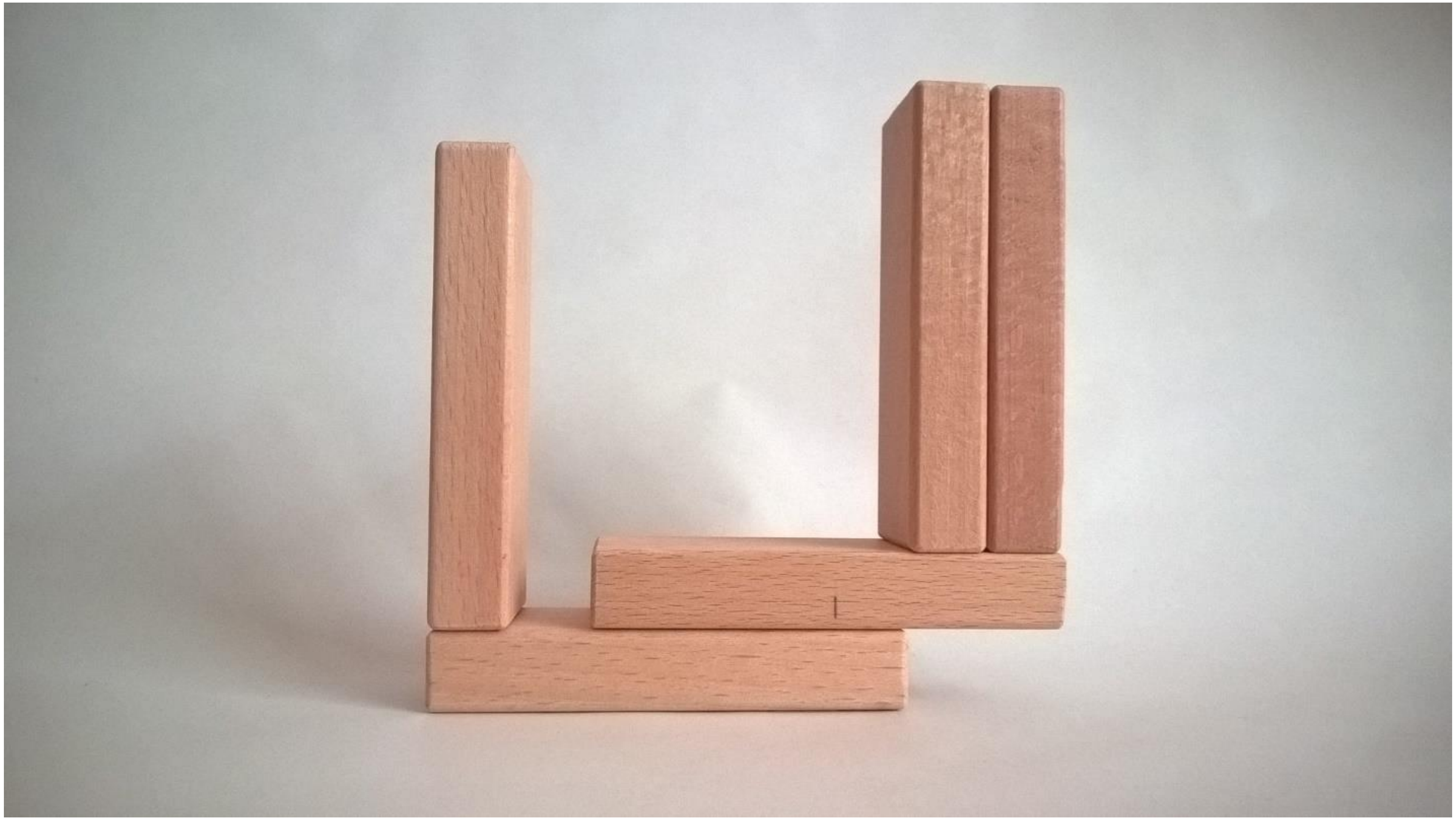

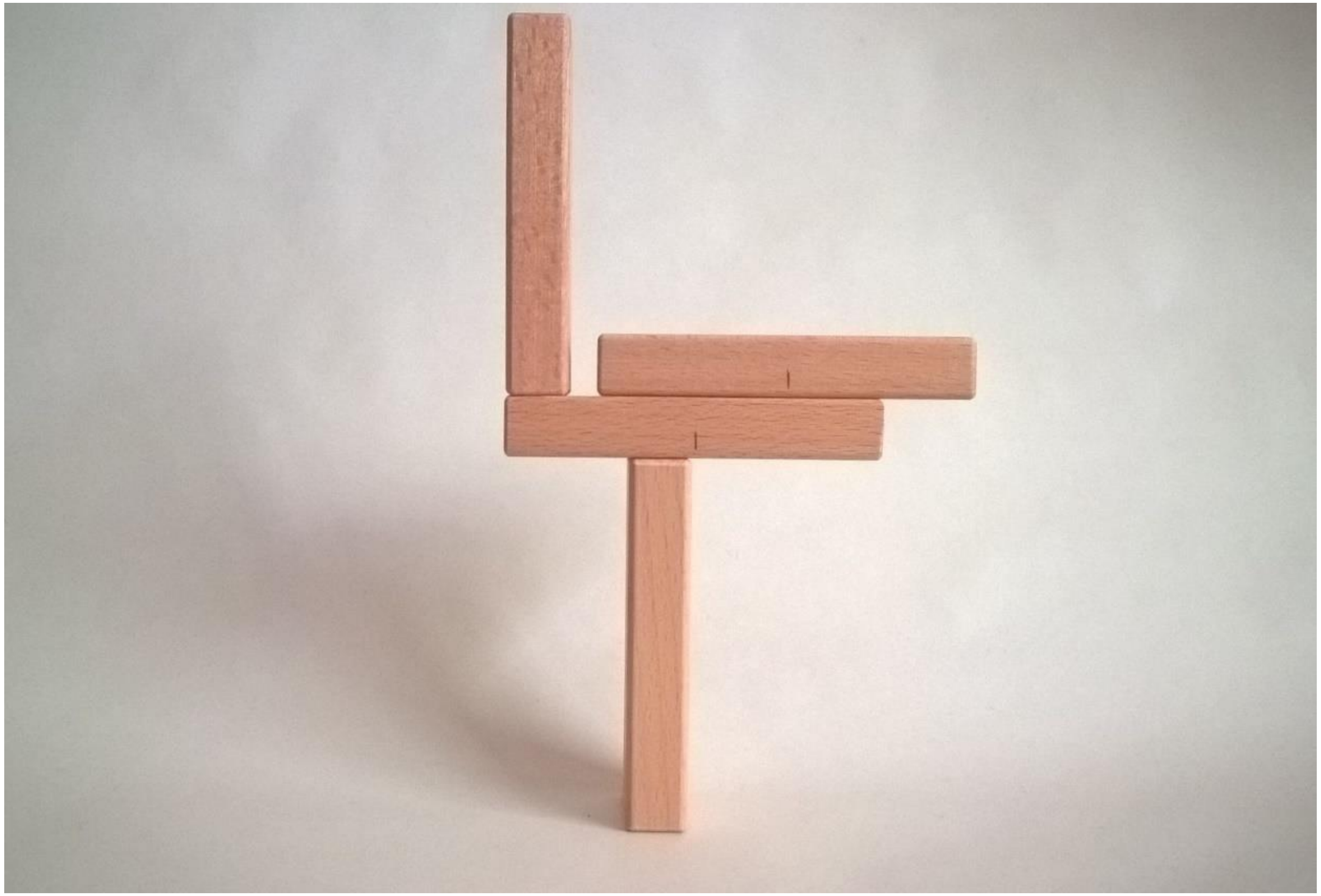

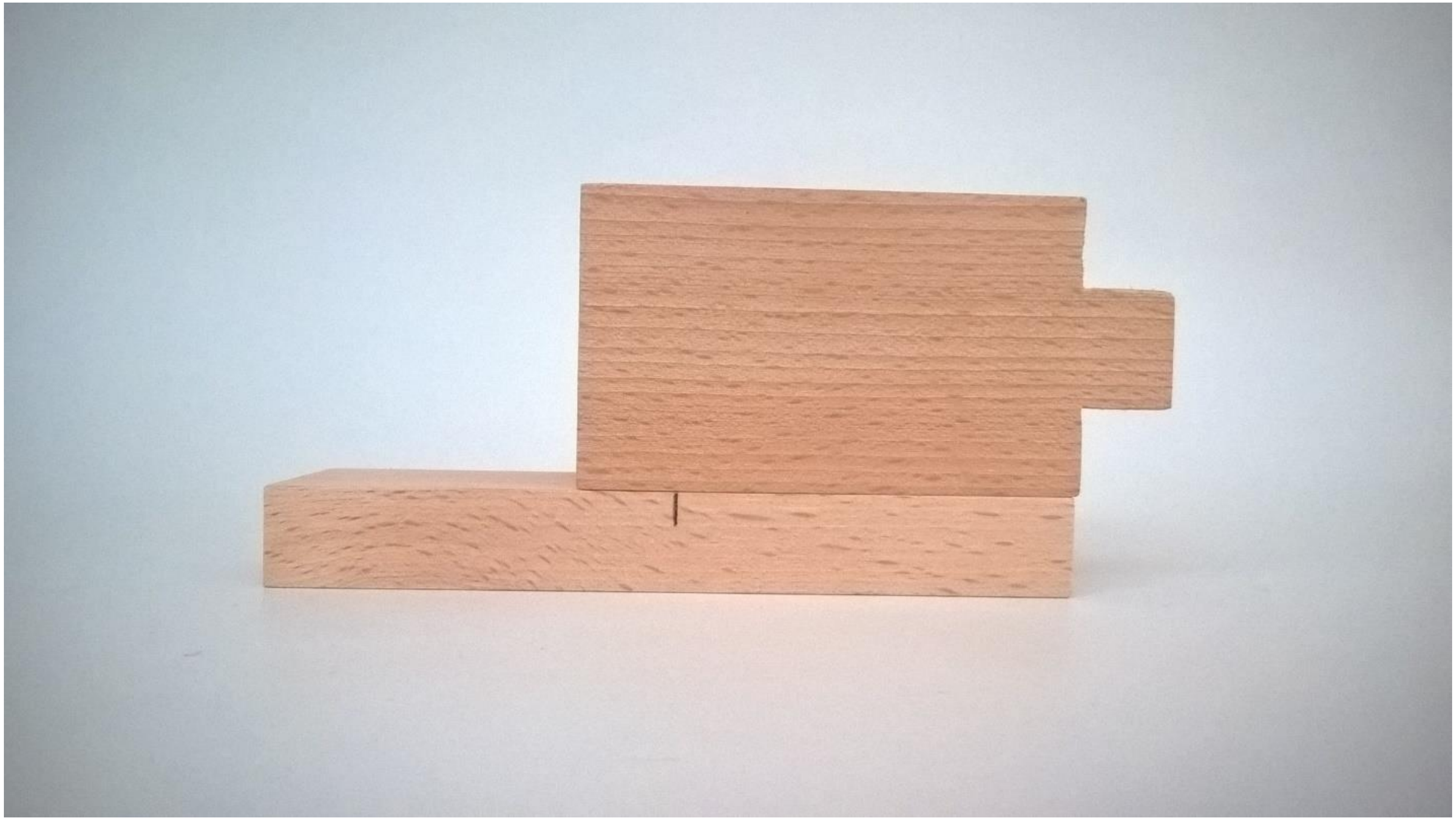

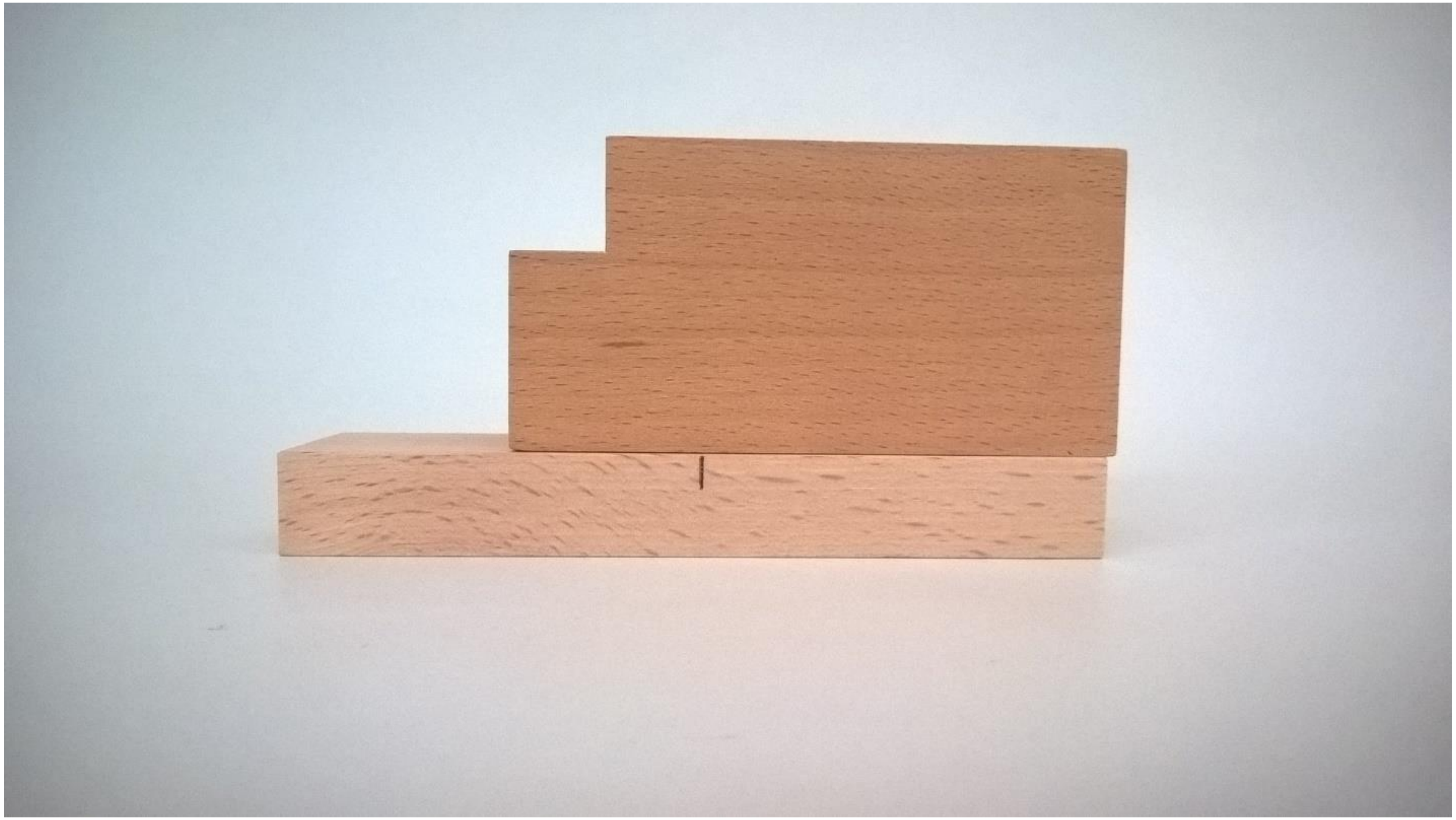

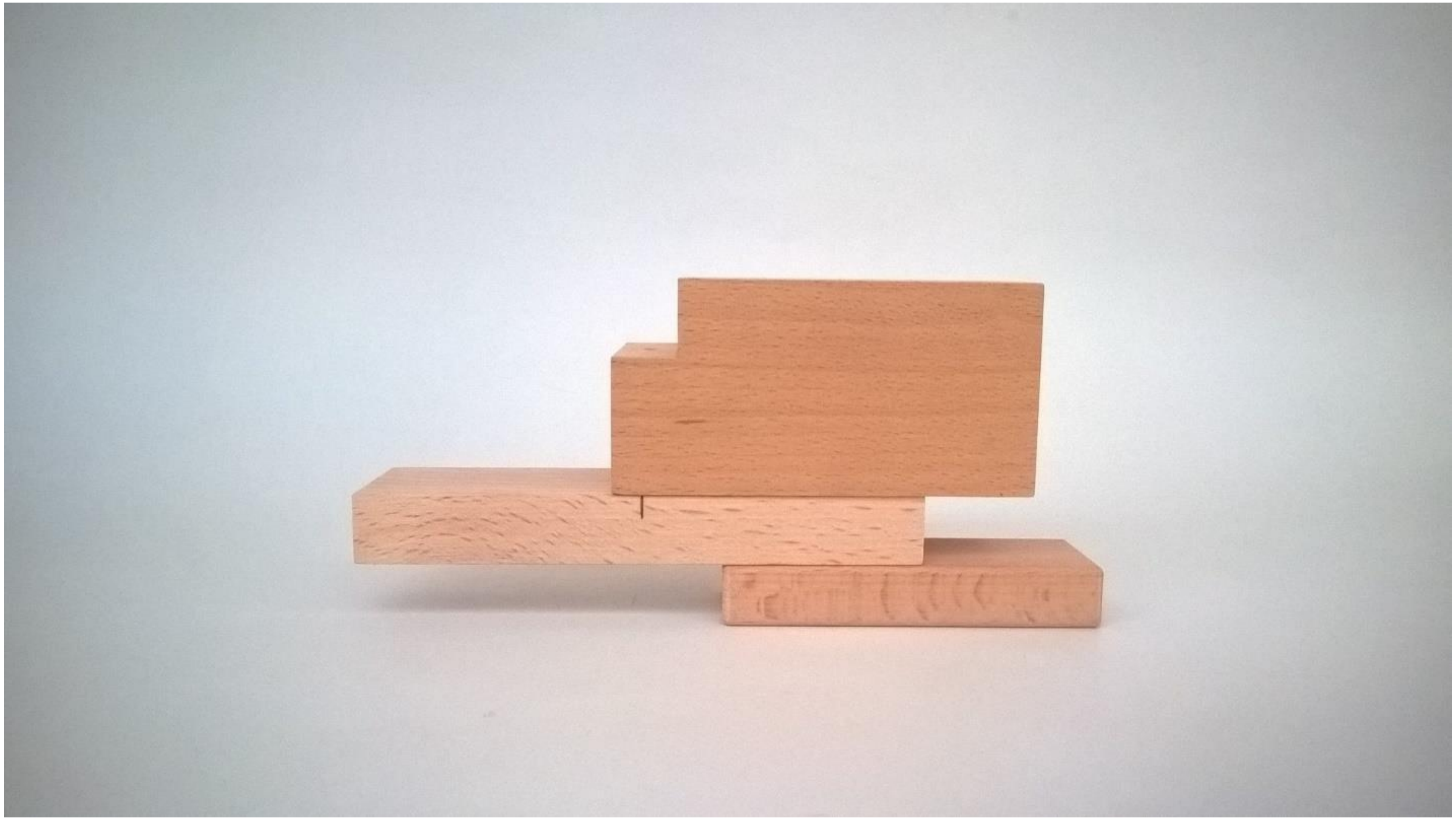

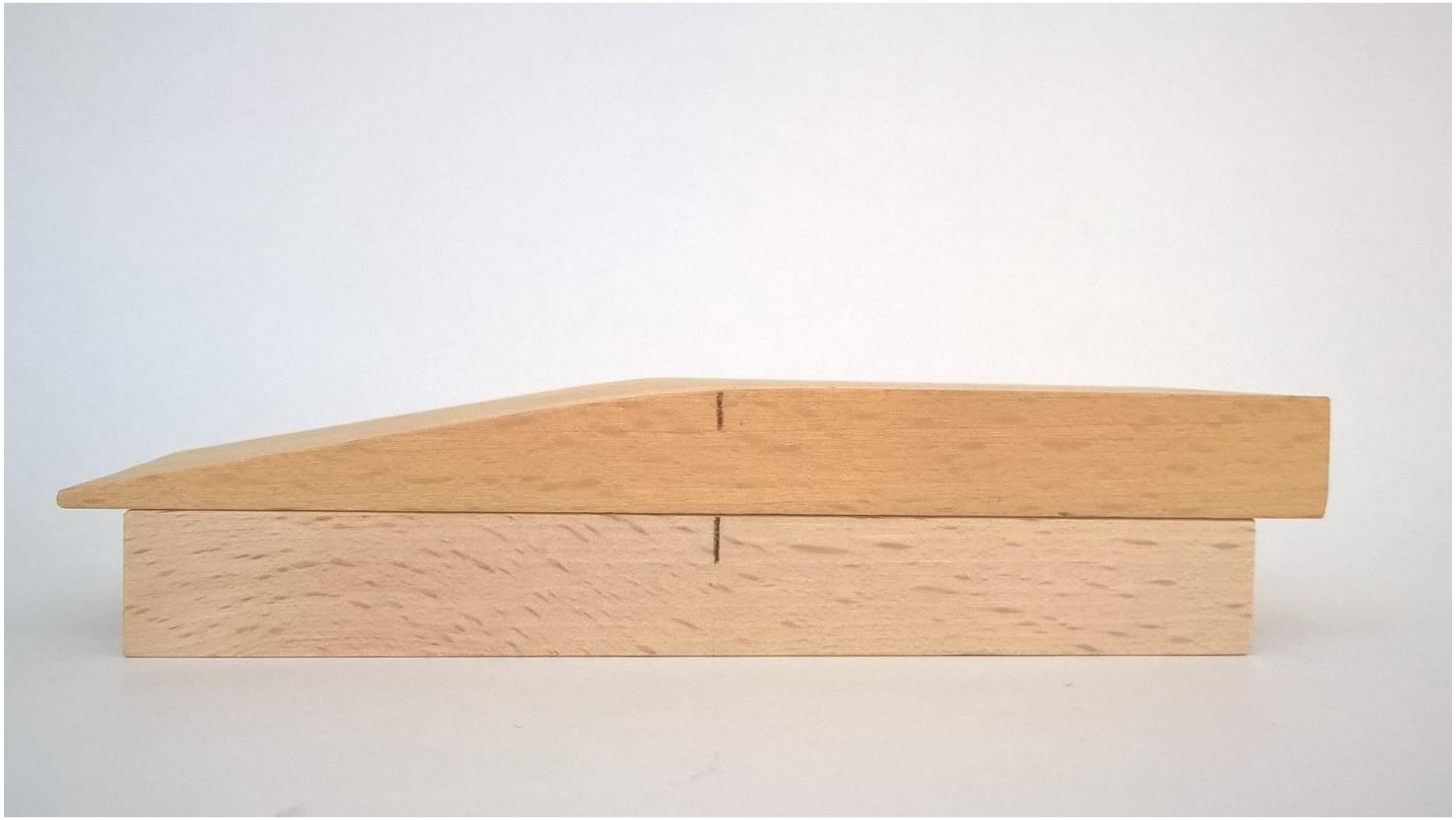

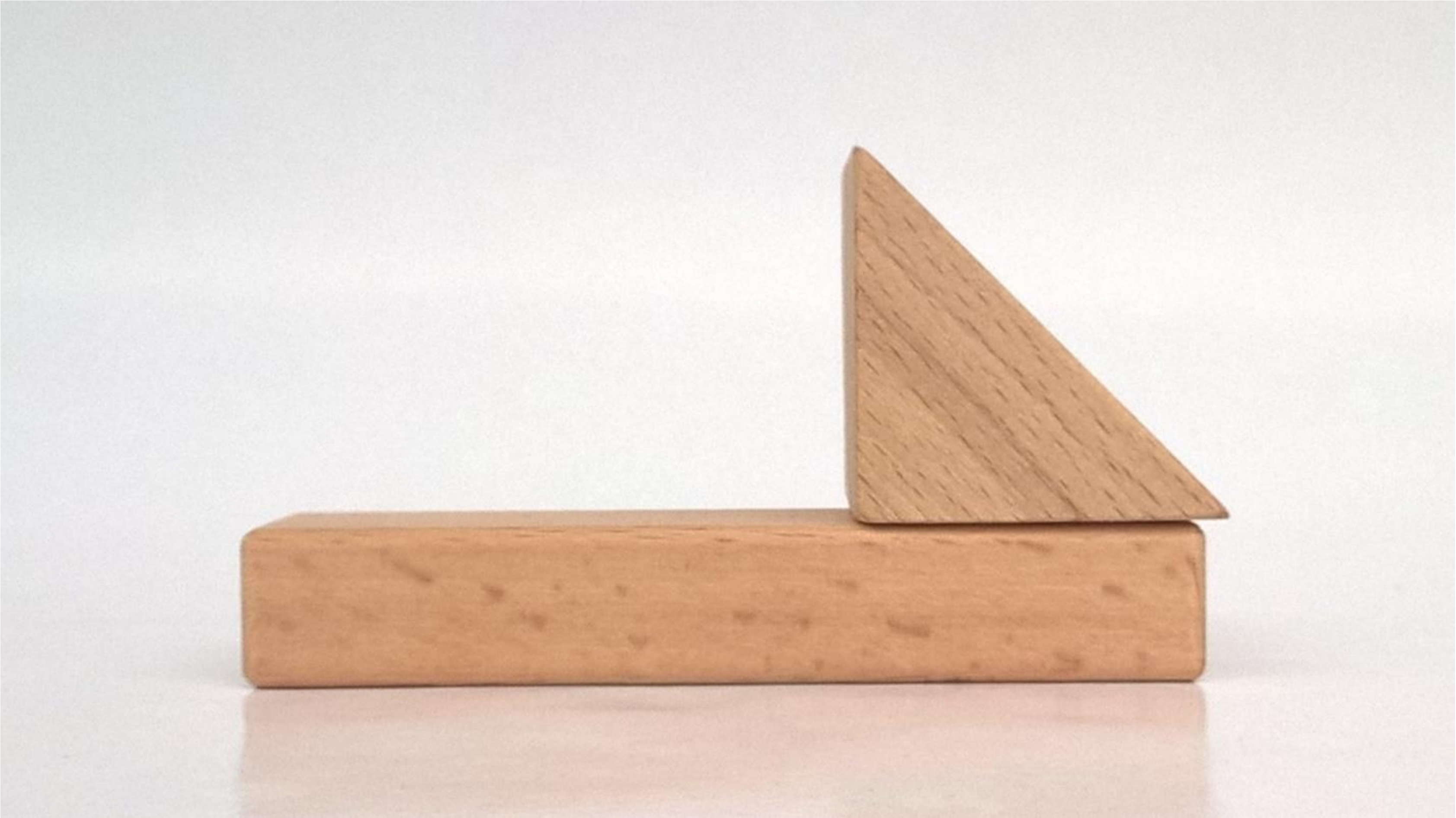

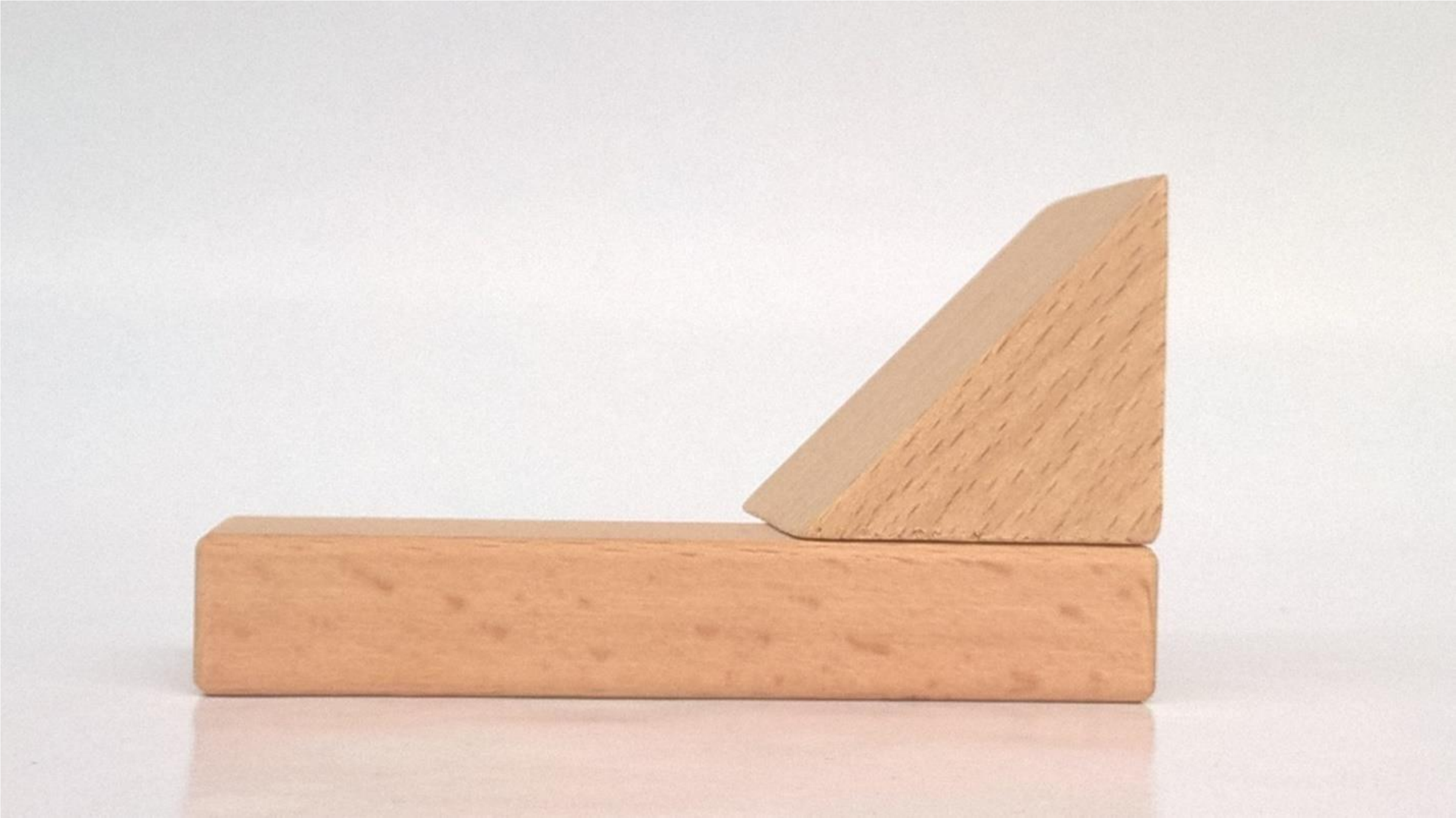

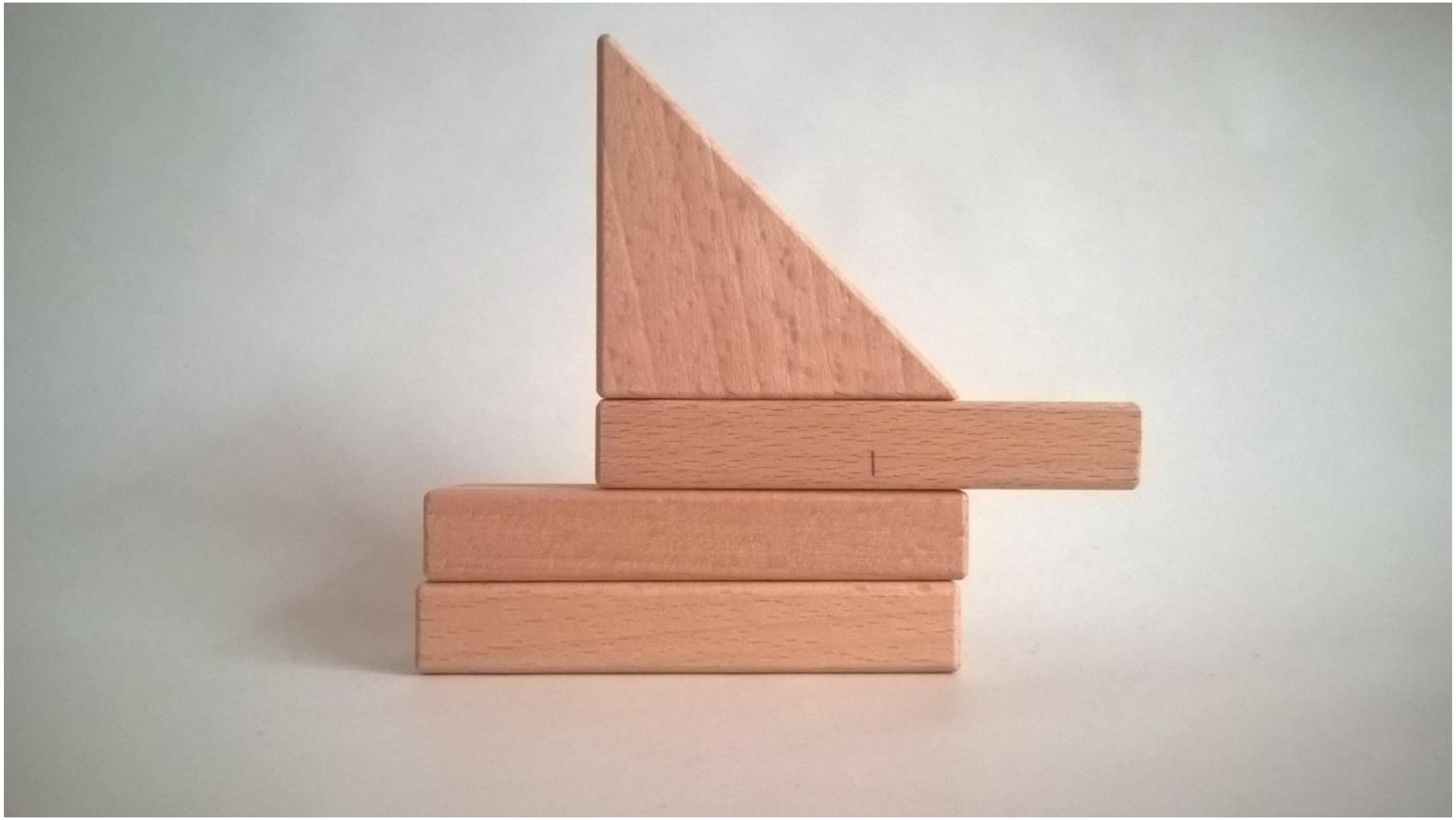

4. Rebuild:

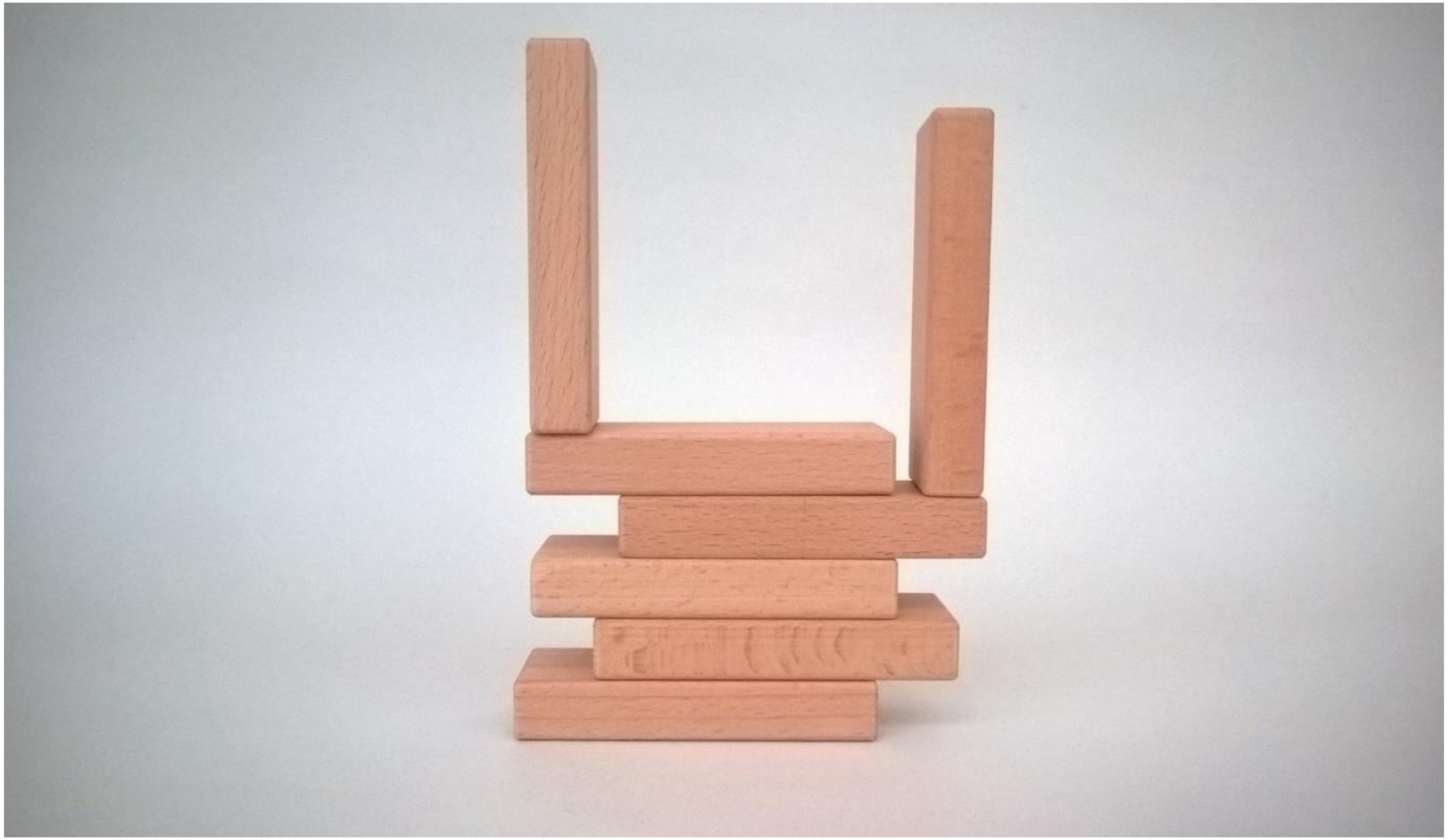

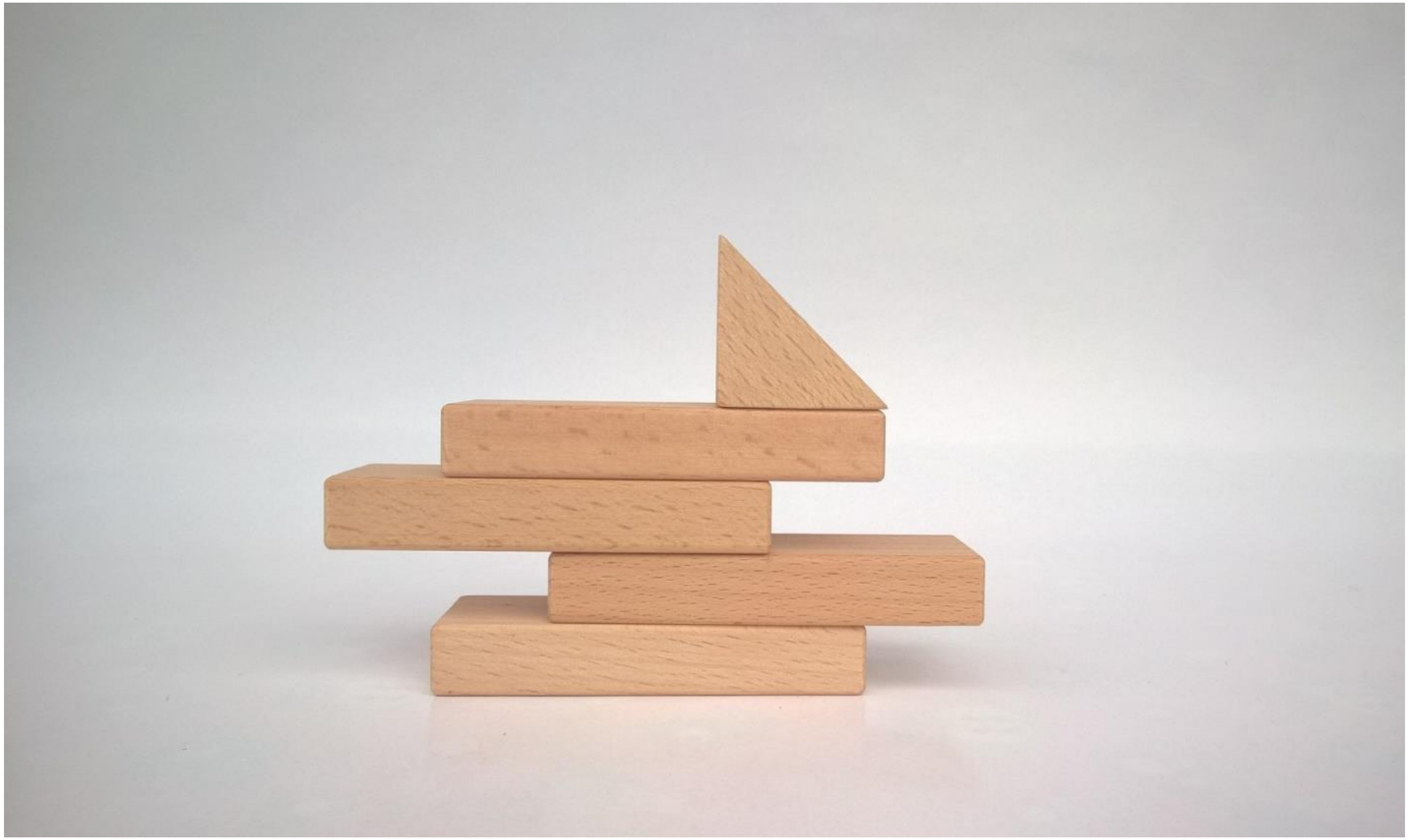

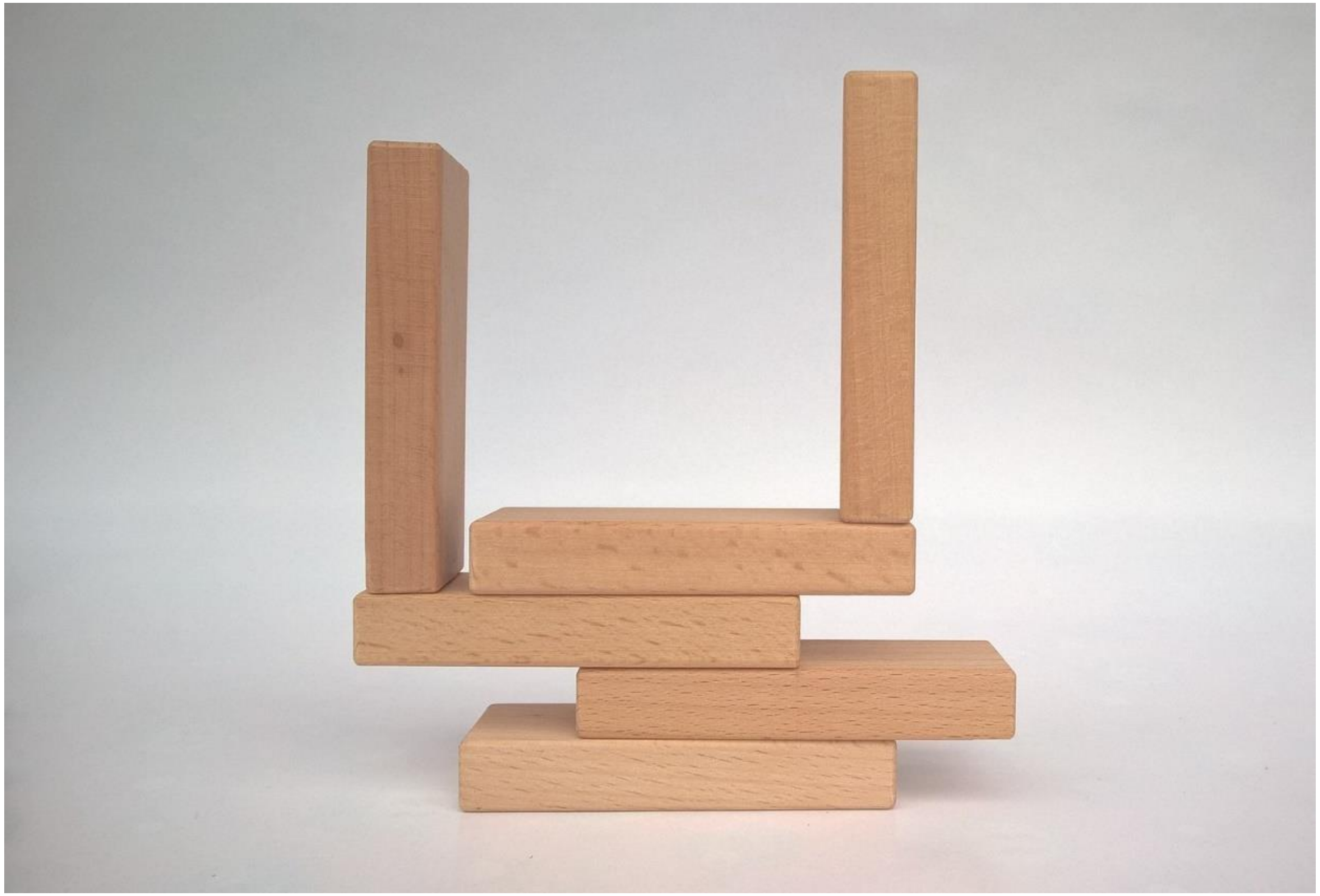

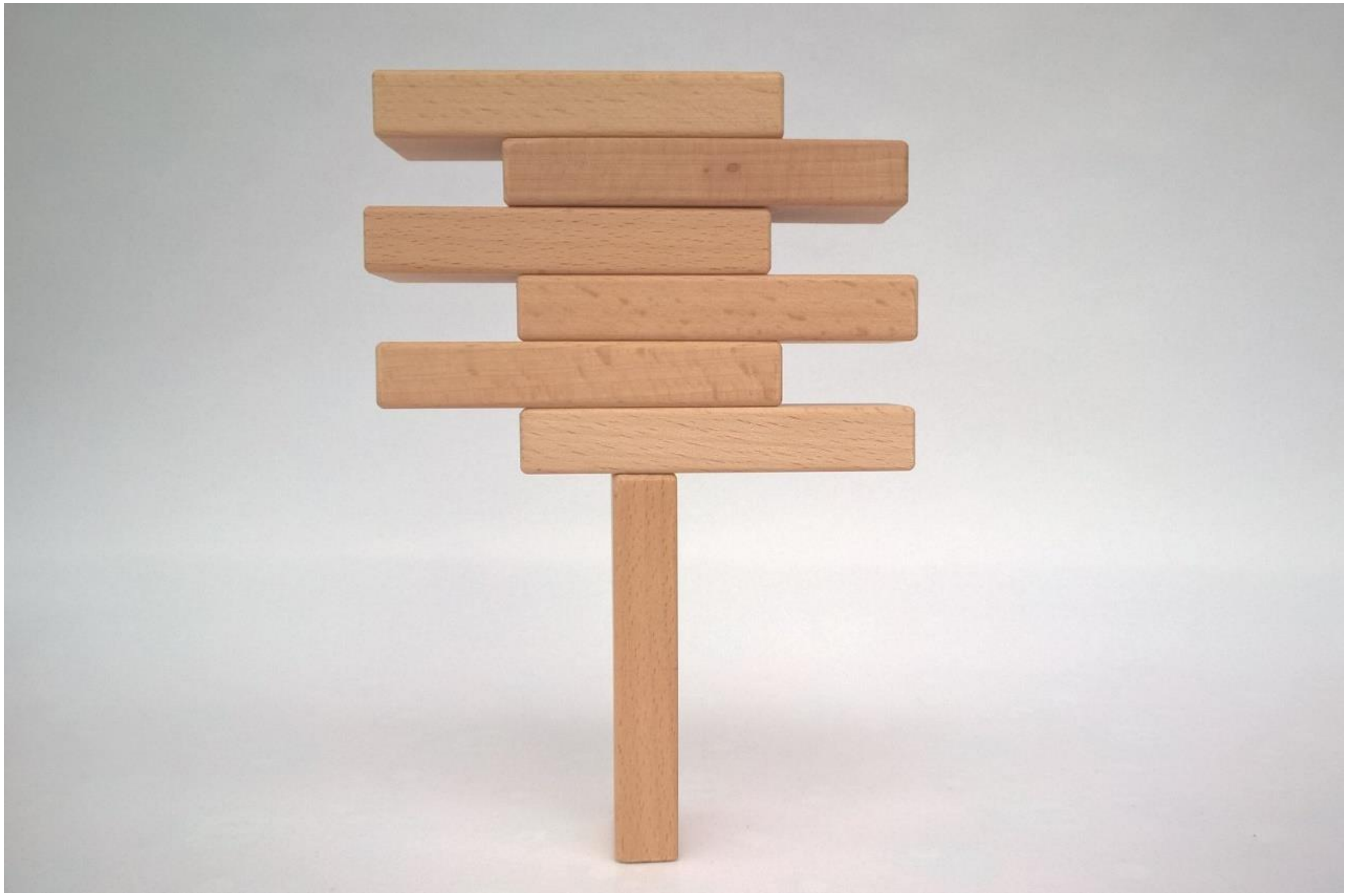

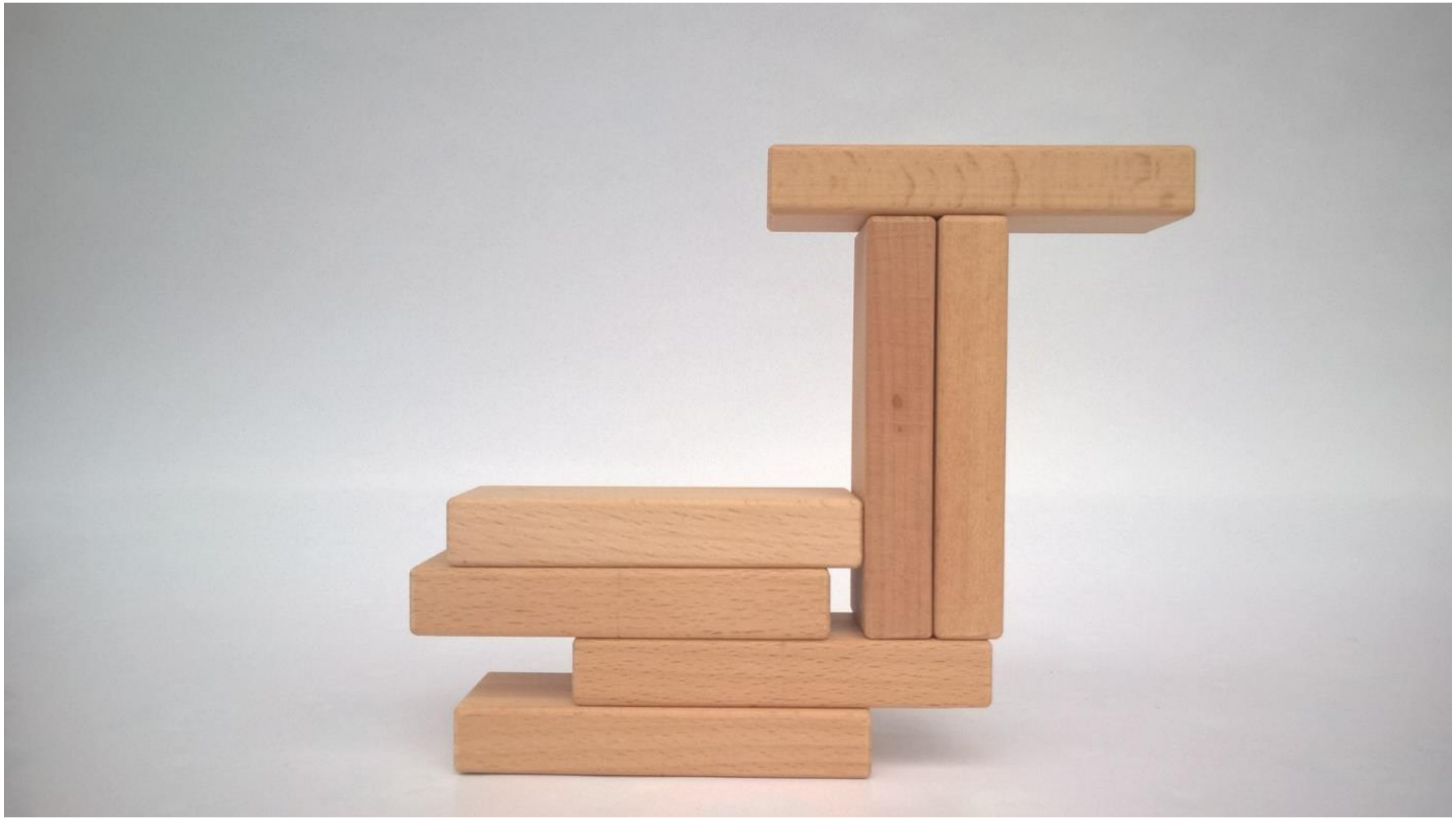

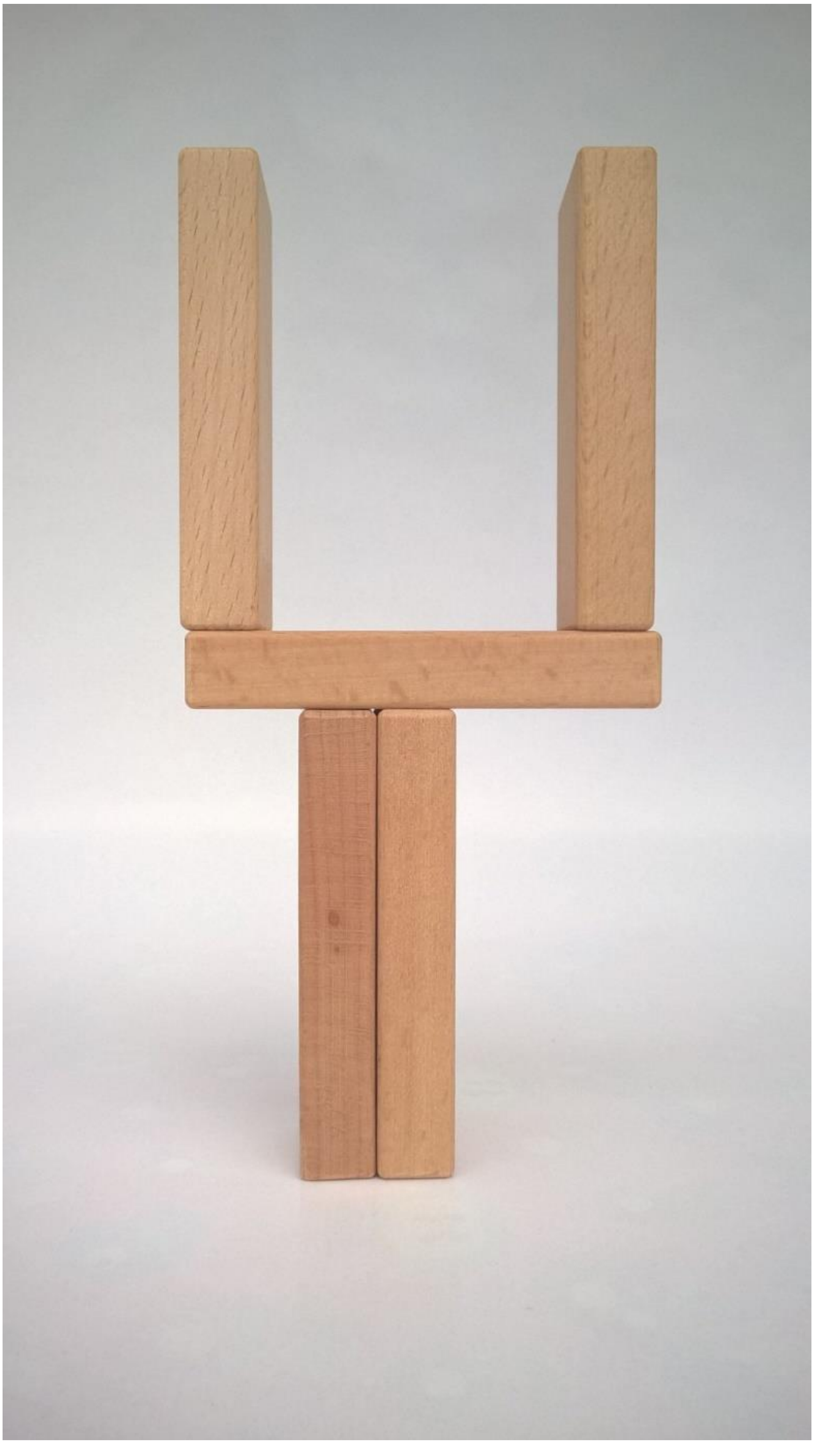

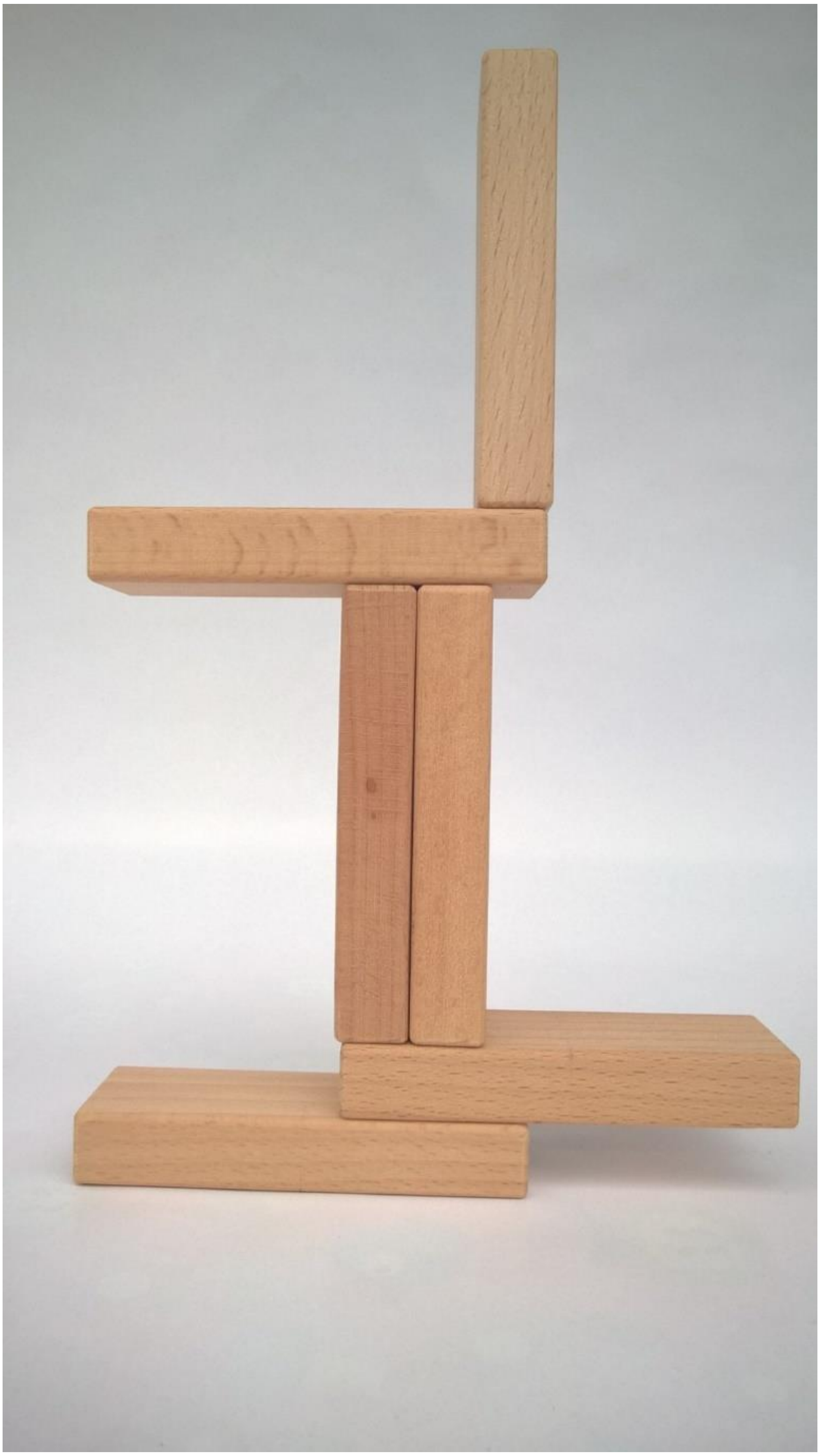

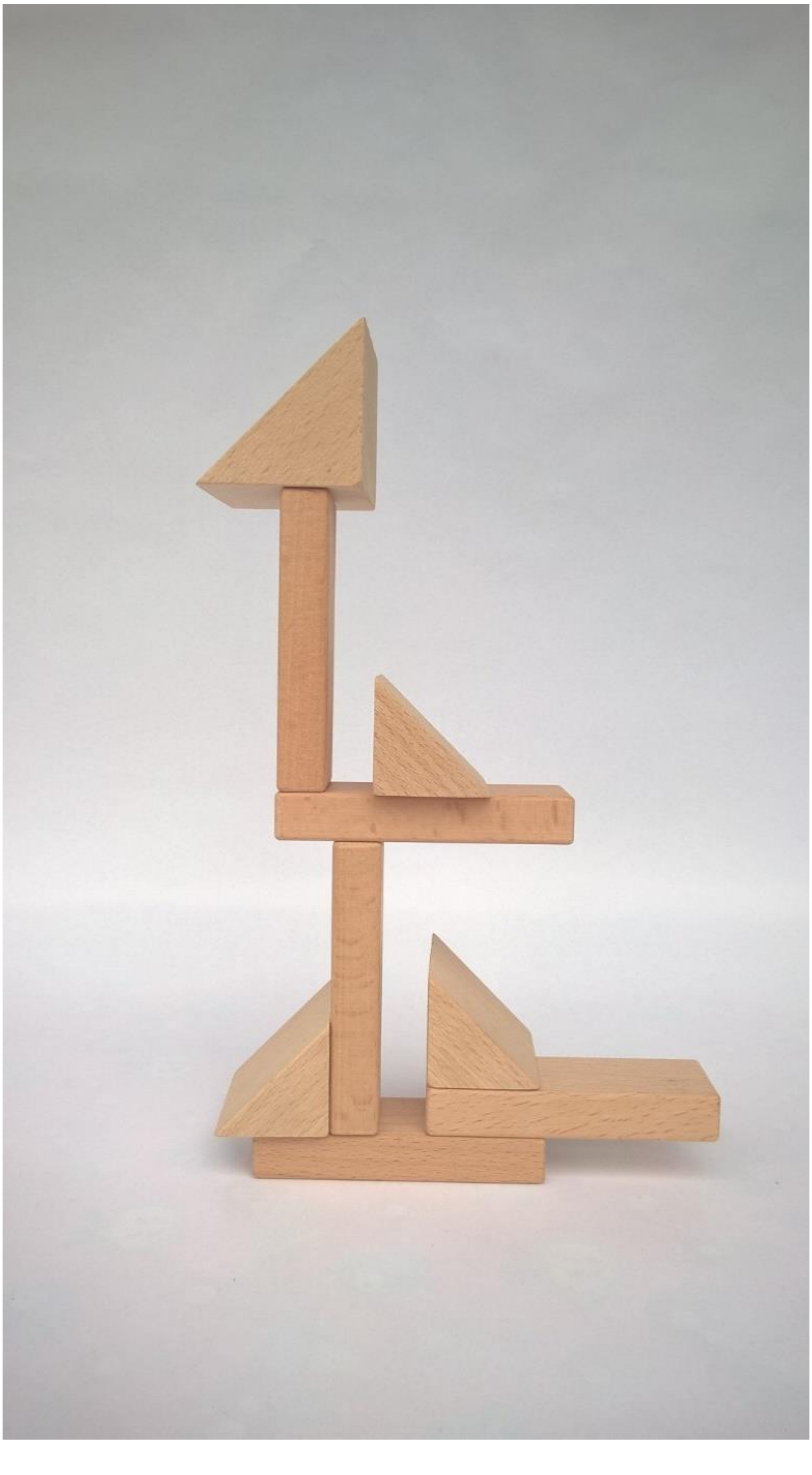

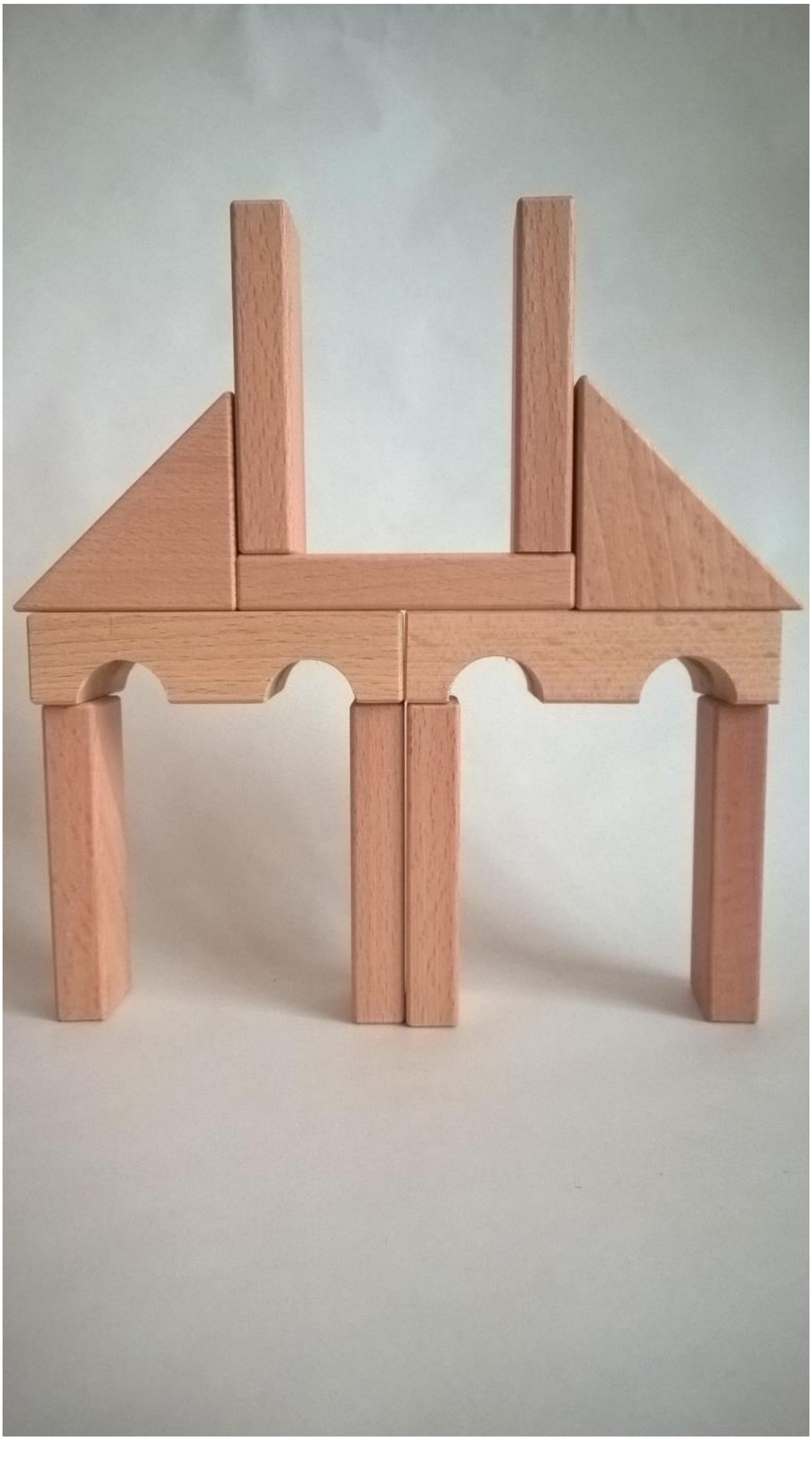

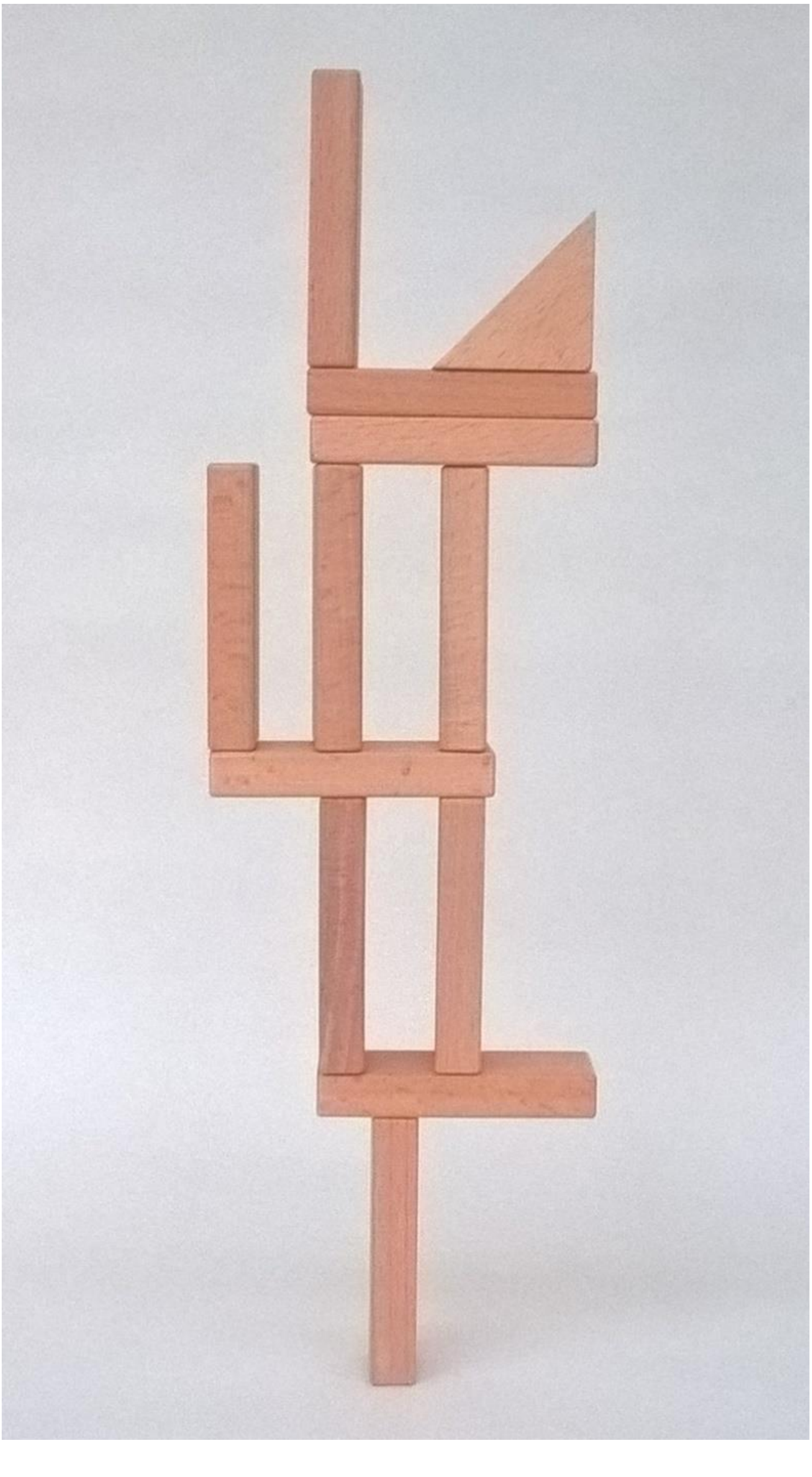

5. Stable/Tumble:

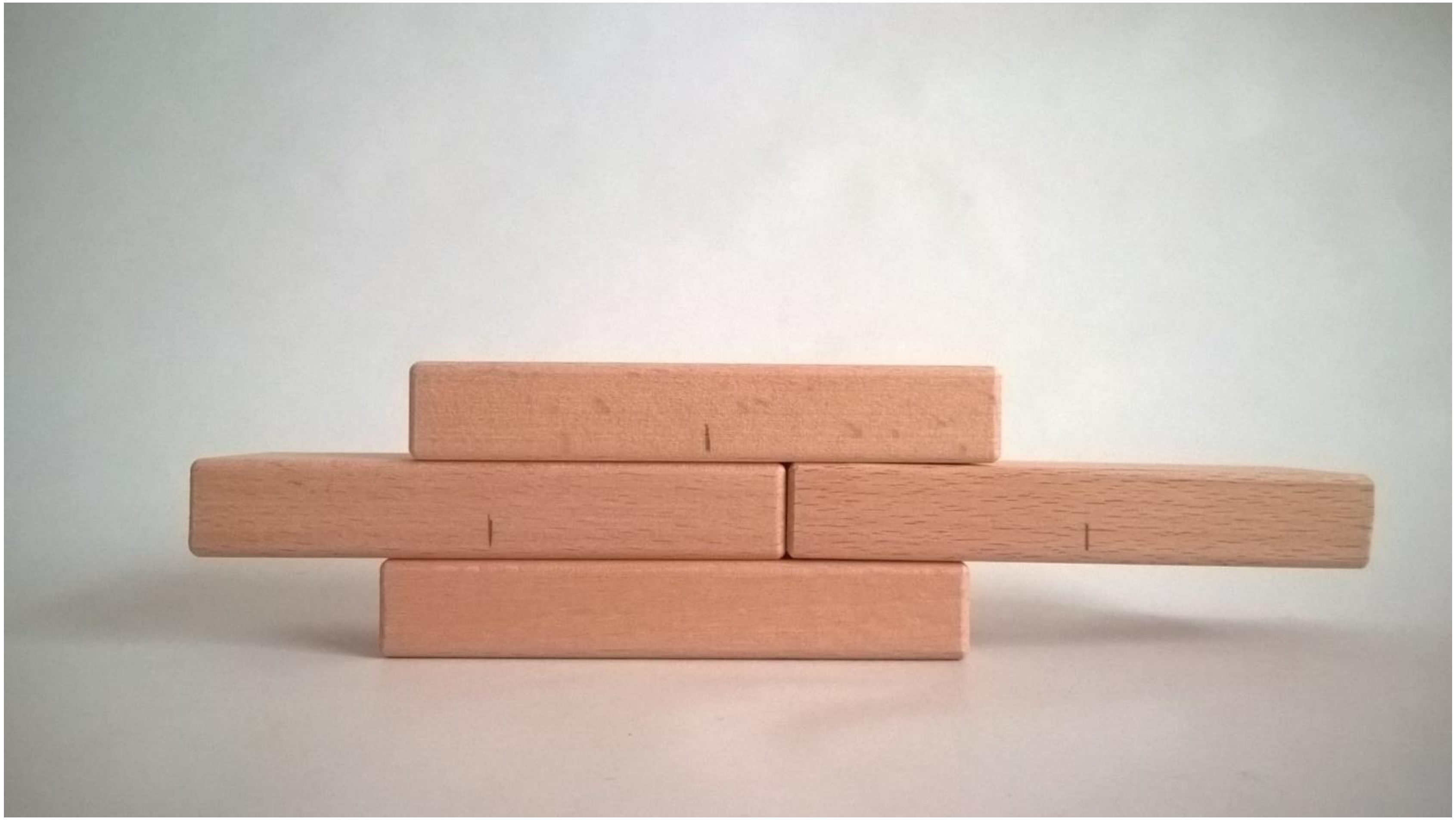

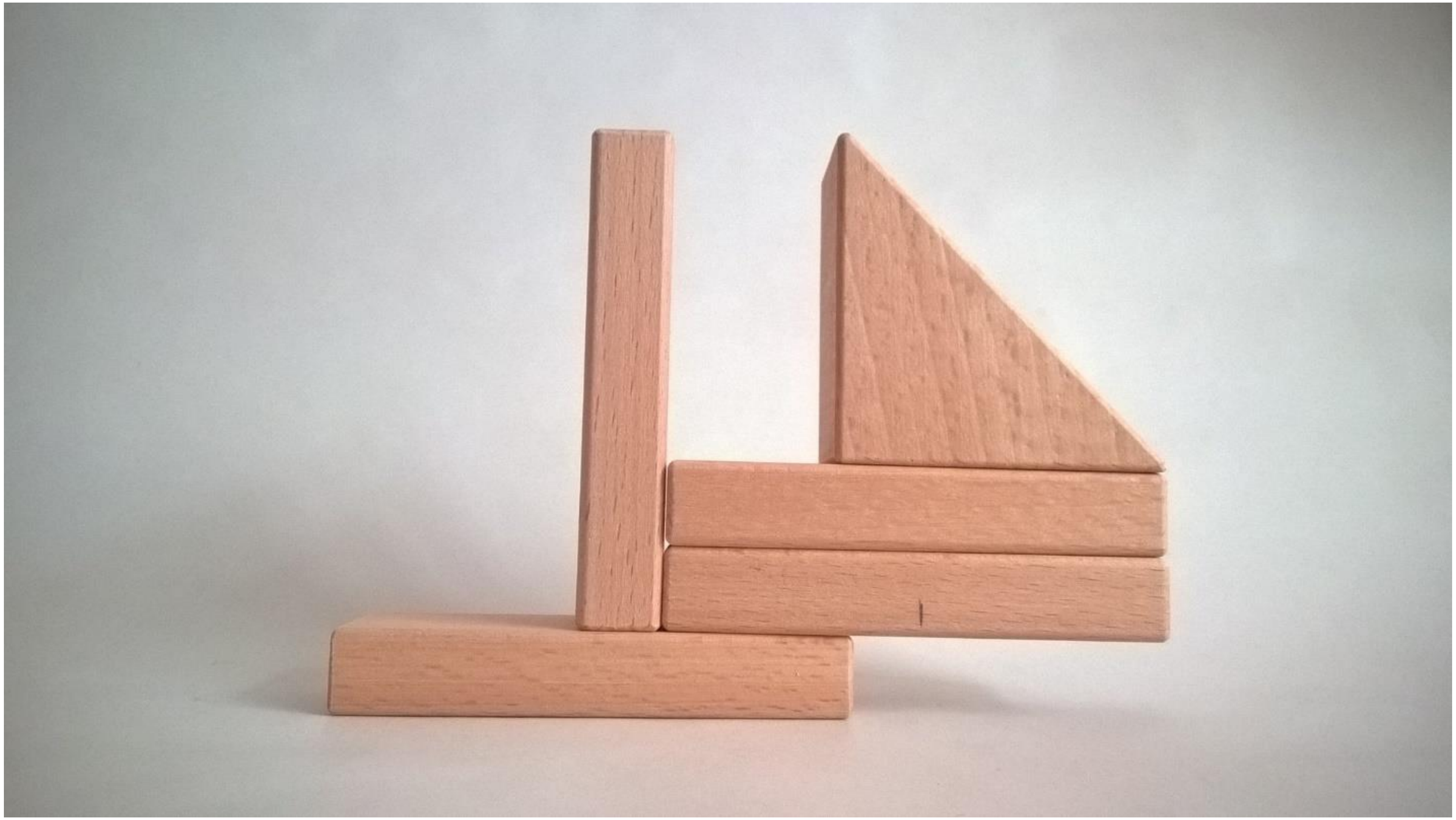

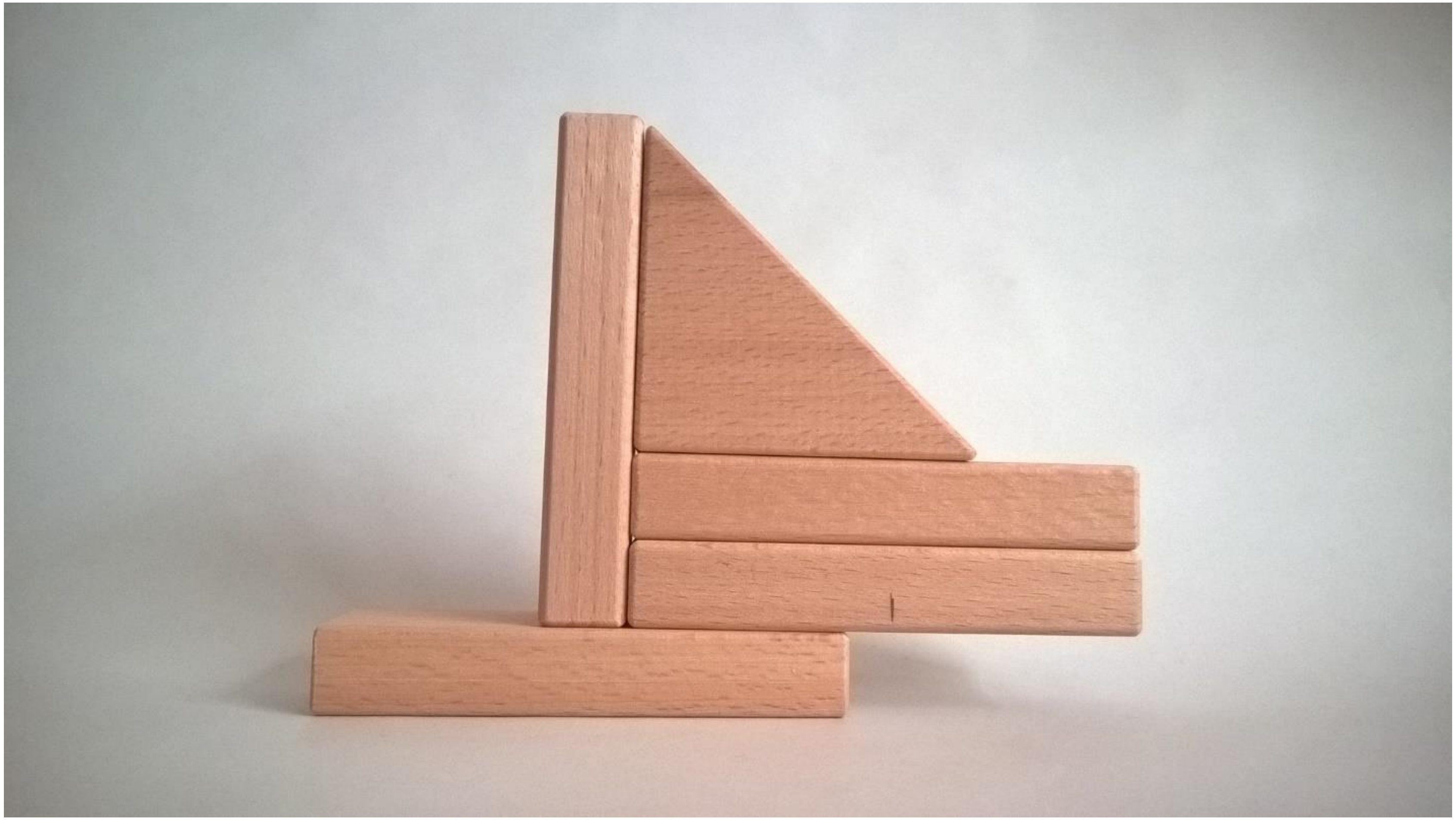

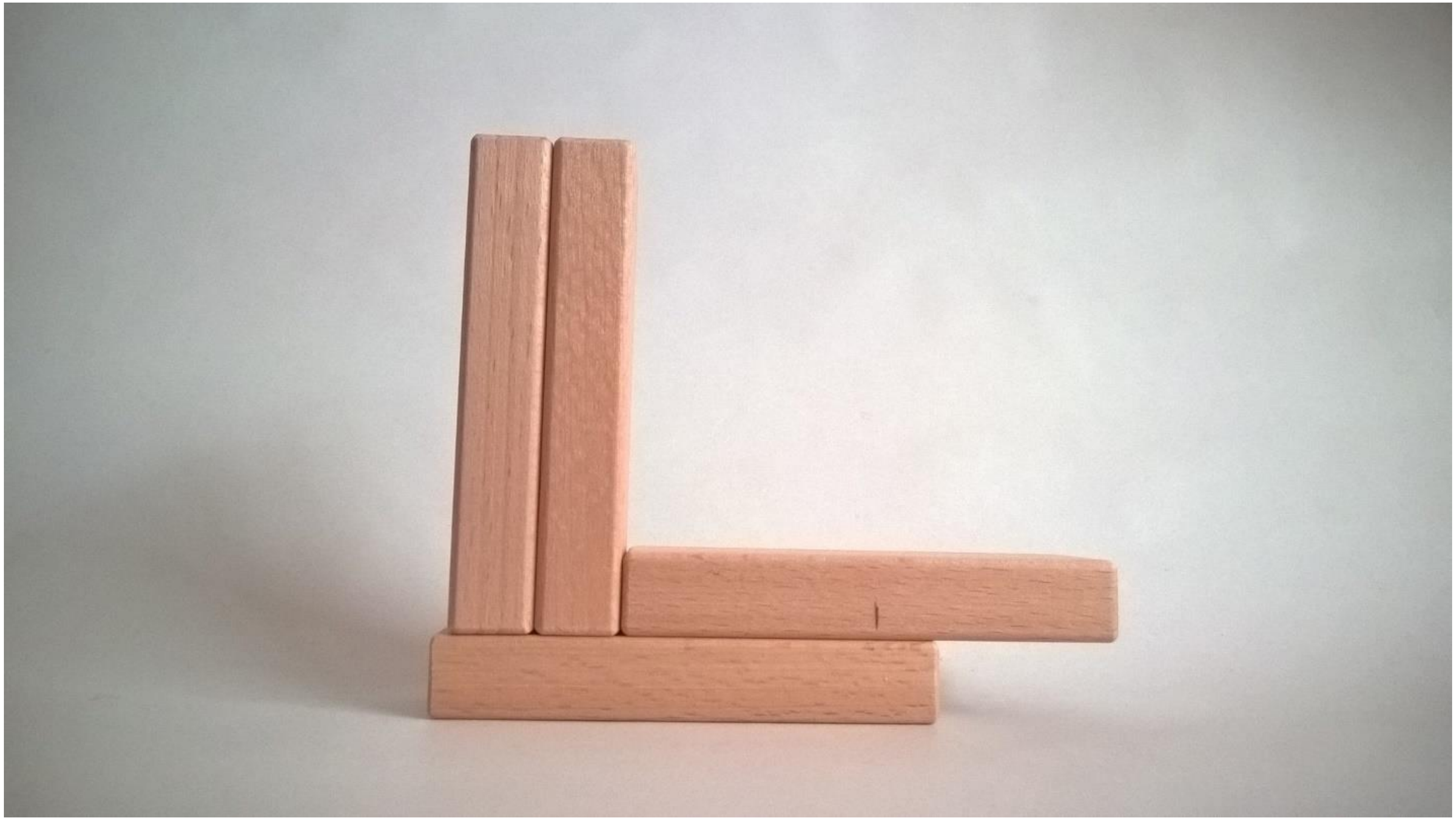

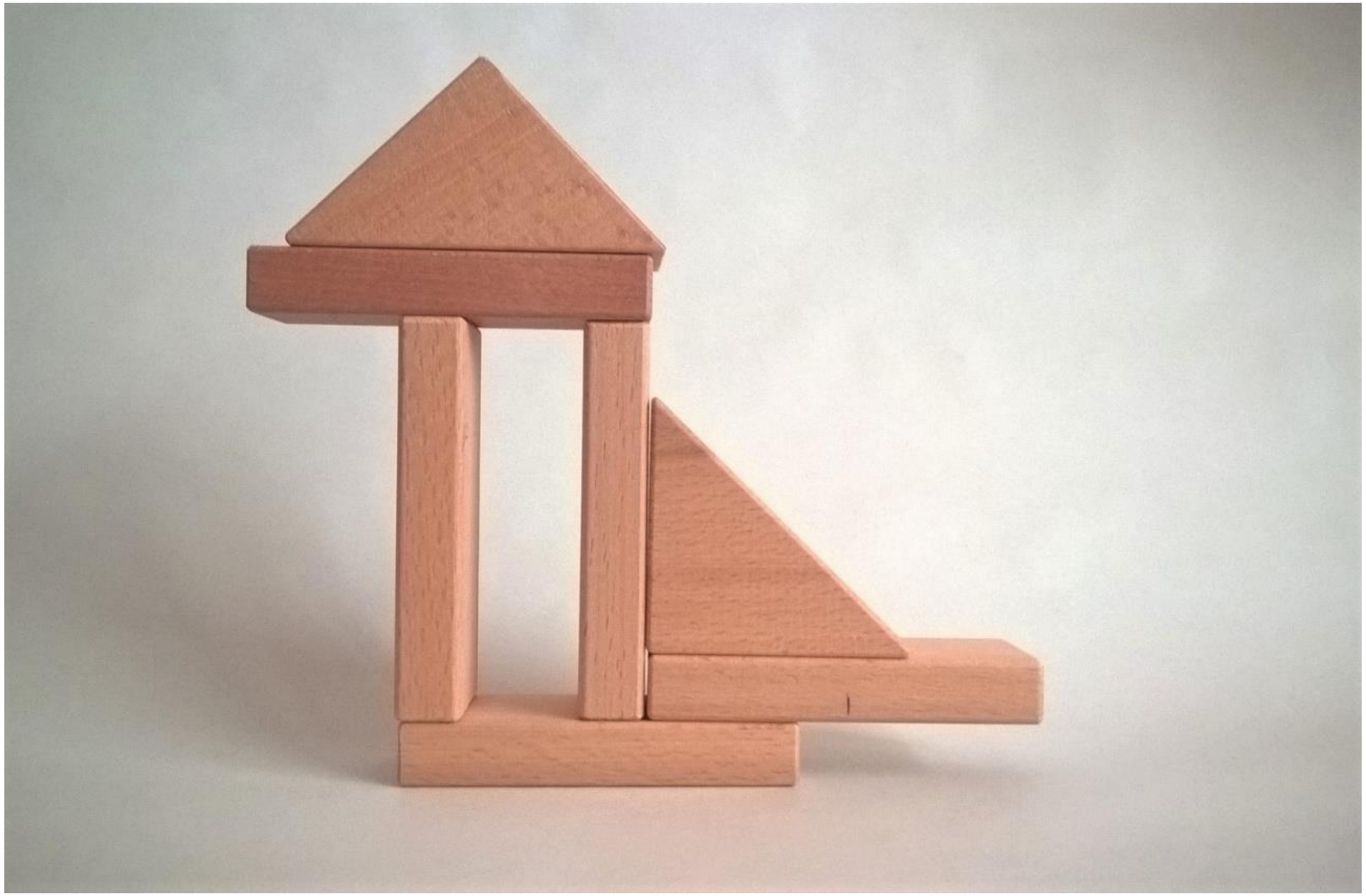

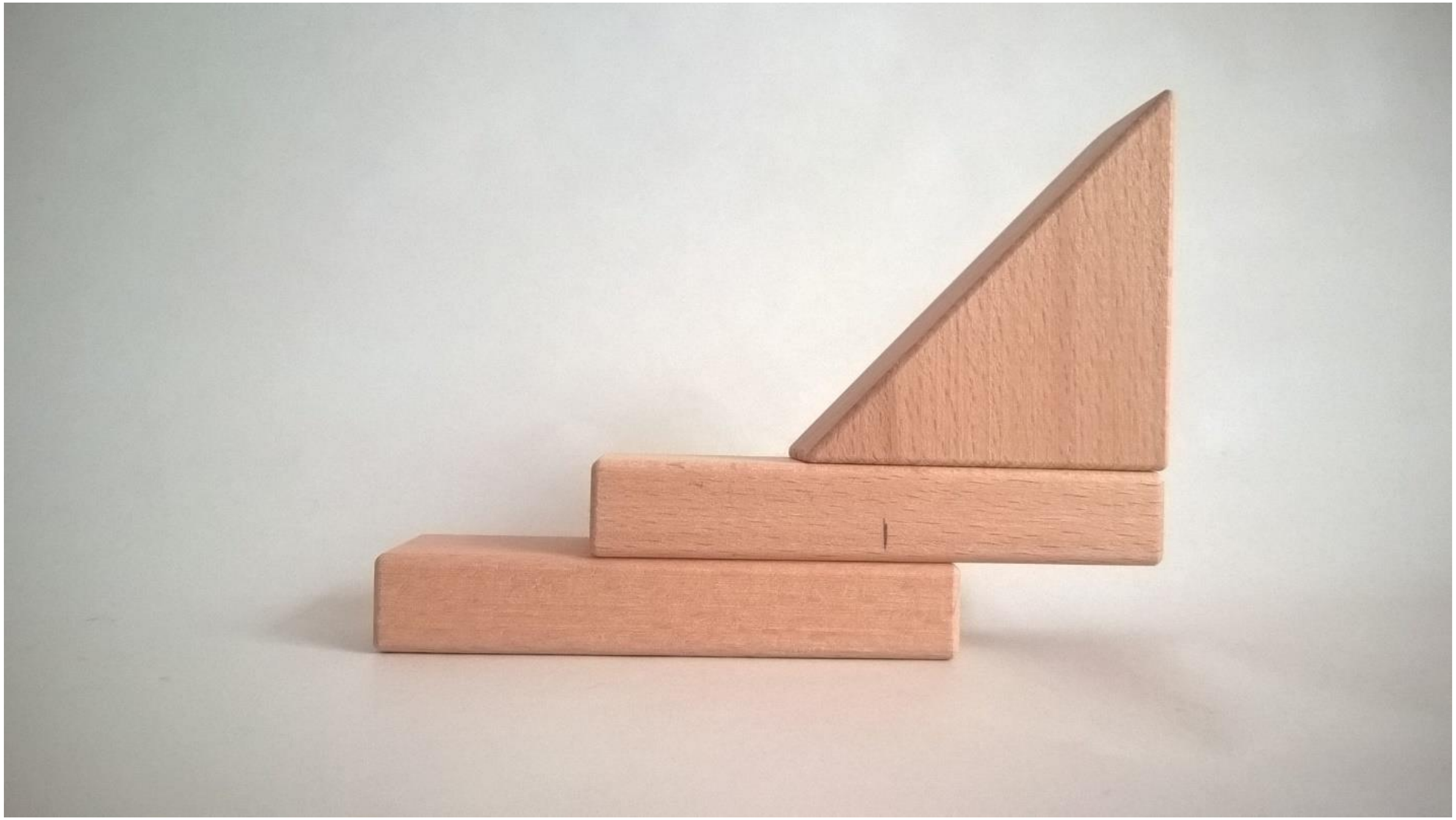

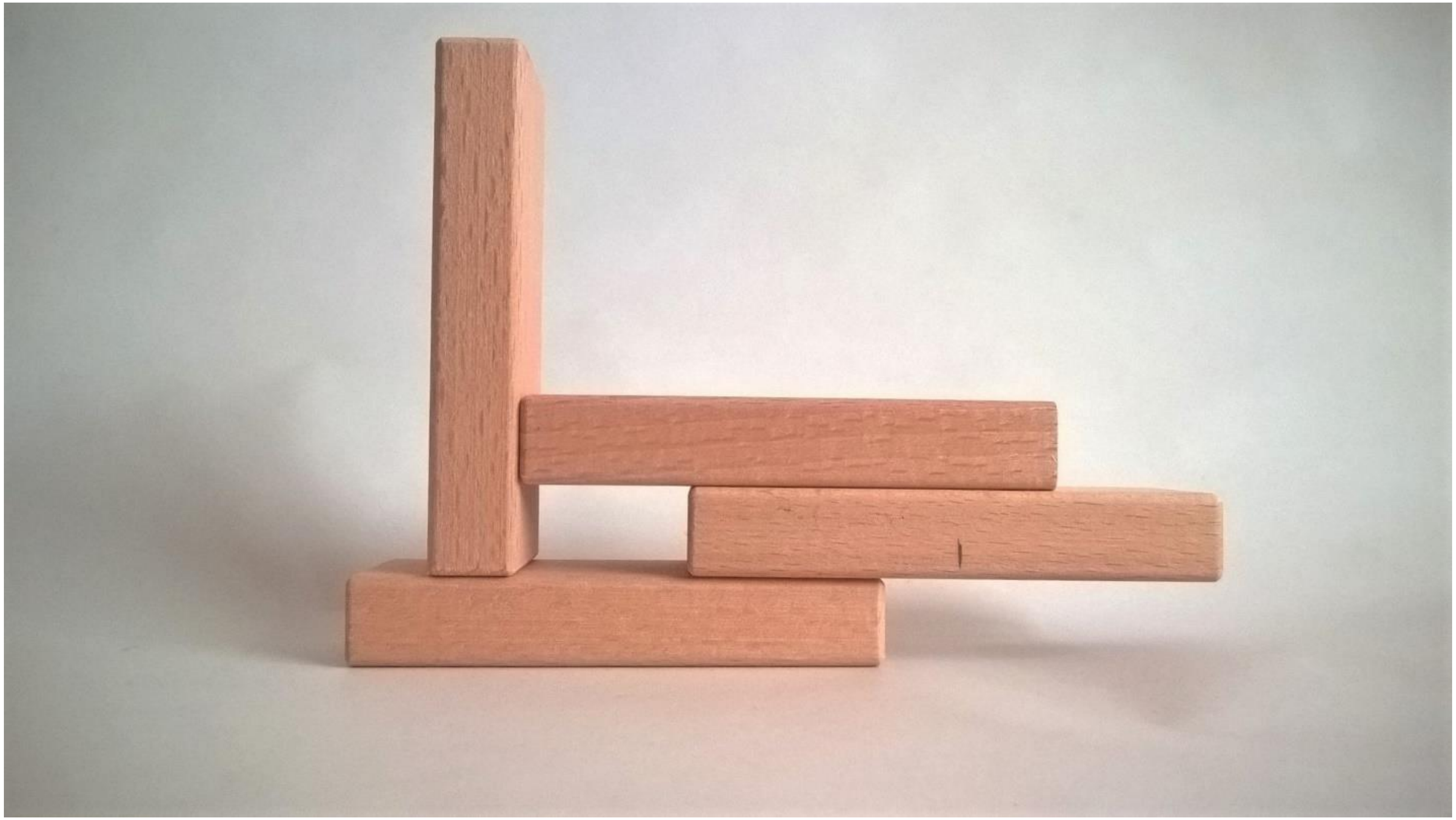

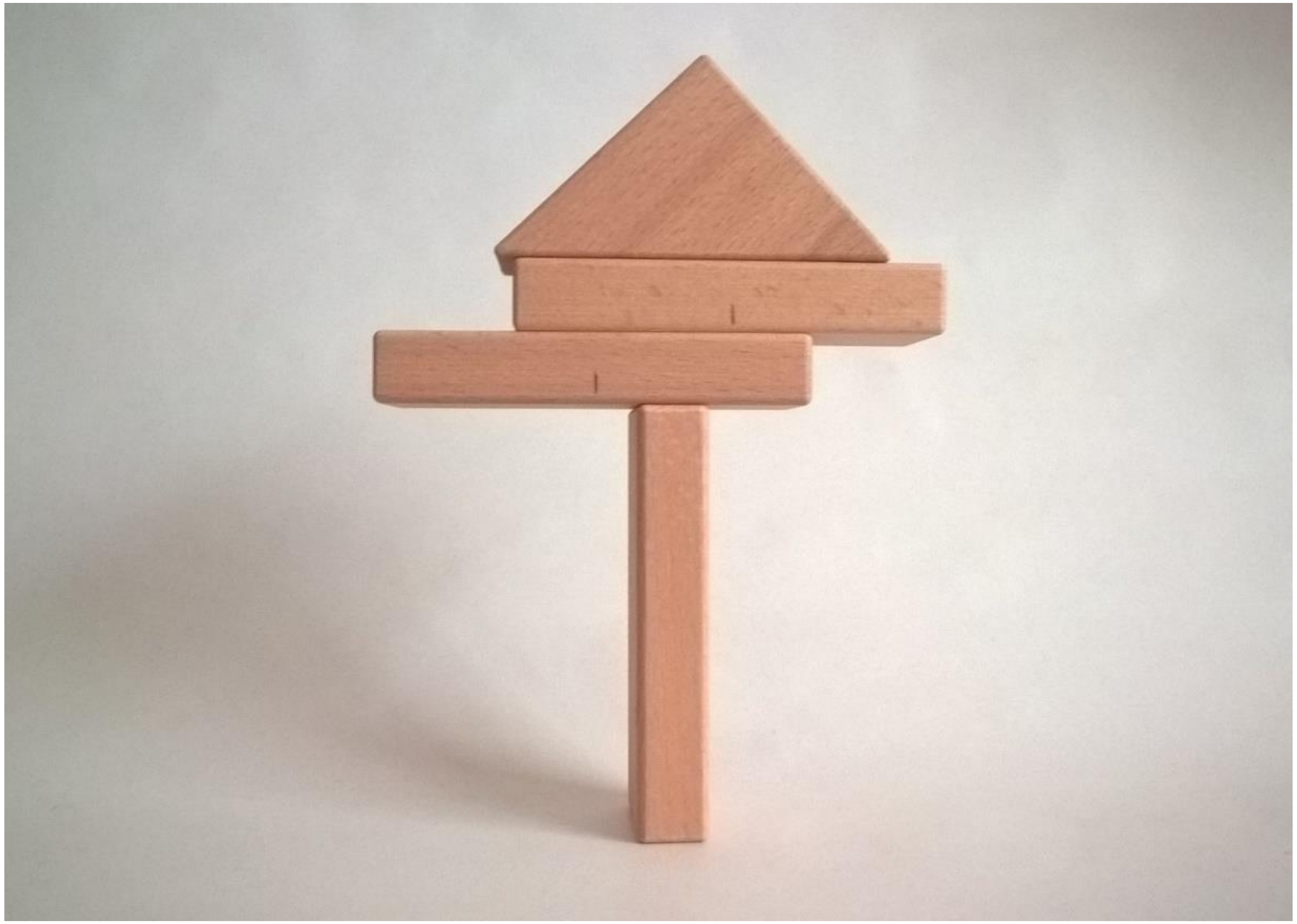

Supplementary Material 2. Script for the verbal scaffolds used during the intervention in the Verbal group.

Activating prior knowledge	Have you ever seen something like this?
	What do you think? Will this remain stable or will it tumble over?
	Do you remember the games we played last time? Now you can try it out yourself.
	When you play with blocks, sometimes blocks tumble over. How high did you build without them tumbling?
	Can you slide these blocks very carefully? Let go of them when they are just supported on the other block.
Encouraging comparisons	Look! What is the difference?
	This building looks different than this one, doesn't it? What is different?
	Look, x built this and y that. Try sliding your blocks along so your building looks like x's.
	Show me, how you have to slide these blocks along.
	How can you compare …?
	What is heavier/lighter/wider/narrower/etc.?
Asking for reasons	Why do you think that?
	What do you mean?
	Why?
	What did you find out?
	Why does it tumble/stand?
	Can you explain this to (finger puppet)?
	Can you explain this to me more clearly?
	Why is that so?
	How did you do this?
	Where are more blocks/is it heavier? On the brown block or on the black block?
	How can you balance this out?
Providing explanations	Exactly! It depends on how the building blocks stand, how much they touch each other.
	Exactly! The building blocks don't always have to stand on the middle to remain standing.
	The building will only remain intact if there are more blocks on the brown block/it is heavier on the brown block.
	If the side with more blocks/the heavier side is dangling in the air, everything tumbles over.
Modelling	Look at these black lines on the blocks. They indicate the block's middle. How do you have to slide these blocks along so that they remain standing or tumble?
	Look at this! (Experimenter looks closely)
	(Experimenter points)
	(Experimenter guides child's gestures)
	(Experimenter turns building blocks with lines and shows how to use them)
	Observe closely.

Supplementary Material 3. Example excerpts from the playful activities (translated from German).

Verbal group, 1 girl and 2 boys from a kindergarten in the periphery:

The children are all building and loudly singing a funny children's song, occasionally they look at each other and chuckle.

Child 1: I will show you that this will remain standing.

Experimenter (to child 1): Look! Do you see this little line (on the picture provided)? There is also a line on this (shows child 1 a line drawn on a building block). This line shows you where you need to place the block's middle.

Child 1: Like this.

Child 2 (motions to his building and looks at child 1 excitedly): Will it tumble or not, child 1?

Child 1: Show me. That will not tumble.

Child 2: I agree.

Experimenter: Child 3, what do you think?

Child 3: It tumbles…?

Child 2 starts to remove the black block.

Experimenter (to child 2): Wait for child 3.

Child 3: Doesn't tumble.

Experimenter: Why not?

Child 1: Here is more burden again (motions to the weight).

Child 3: I don't know.

Experimenter: Okay, but maybe what child 1 said is correct.

Child 2 (excited): Because there is more space, because there is more space!

Child 1 (leans over the table to child 2 and pointing at the building explains to him): There is quite a lot more here (motions to heavier side). There and there is only a bit.

Child 2 removes the black block, smiles and waves his arms: It remains standing!

Experimenter: Super! Ace!

Child 2 smiles proudly.

Experimenter: Because there are more blocks on this side, it remains standing.

Child 2: Yeah.

Material group, 2 girl and 2 boys from a kindergarten in a village:

The children are building in pairs.

Child 1 (excitedly to child 2): That will stand, I know that.

Child 2: I agree.

Child 3 (to child 3): I know that this will stand. And you?

Child 4: Hm, that has to be farther away from another.

Child 3: Yeah.

Child 1 (smiling proudly at experimenter): Yes, it falls. We did know that.

Experimenter nods.

Child 1: Should we build that next? (pulls blocks and picture to himself and child 2)

Experimenter: Sure.

Child 1: I know that this will tumble.

Child 3 and 4 have finished building, they smile at each other and then at the experimenter proudly.

Child 4: Will it stand or tumble?

Child 3: I think that it won't stand.

Child 1 starts singing a made-up song.

Child 4 removes black block, while child 3 stabilizes building. She lets go, building tumbles.

Both children smile a bit sadly at each other.

Child 3 (to child 4 in a comforting voice): Oh well. That's not that bad.

Child 1 restarts singing his made-up song.

Child 3 and 4 choose another picture and the blocks.

Child 3: Eh? What do we have to do there? Do we have to put the black block over here and on there?

Experimenter: Exactly.

Free play group, 2 boys from a kindergarten in a city:

Child 1: What should we be building?

They both build in silence.

Child 1: Oh, I need that one.

Child 2: I need this one.

Child 1 accidentally shakes the table, while he reaches for more blocks. His building tumbles, he looks at child 2 accusingly. Child 2 starts to laugh and child 1 joins in. Both resume building.

Child 1 starts singing while building.

Child 2 reaches for the blocks and accidentally shakes the table, his building and child 1's building both tumble. Child 2 laughs.

Child 1: Child 2, you shake everything!

Child 2: No, I don't.

Child 1 starts to build something different: Look, I'll build it like that.

Supplementary Material 4. Items of the reasoning test.

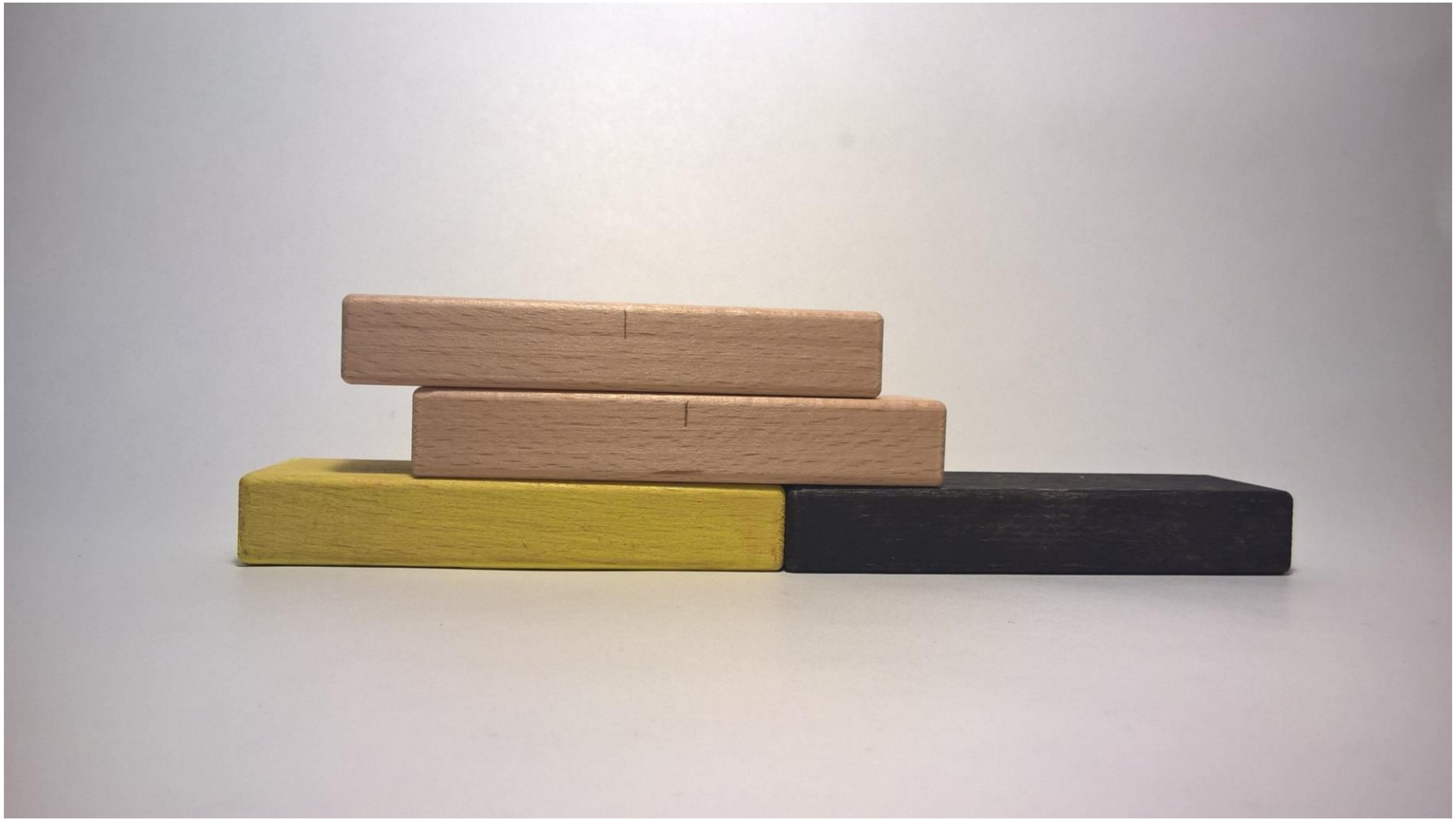

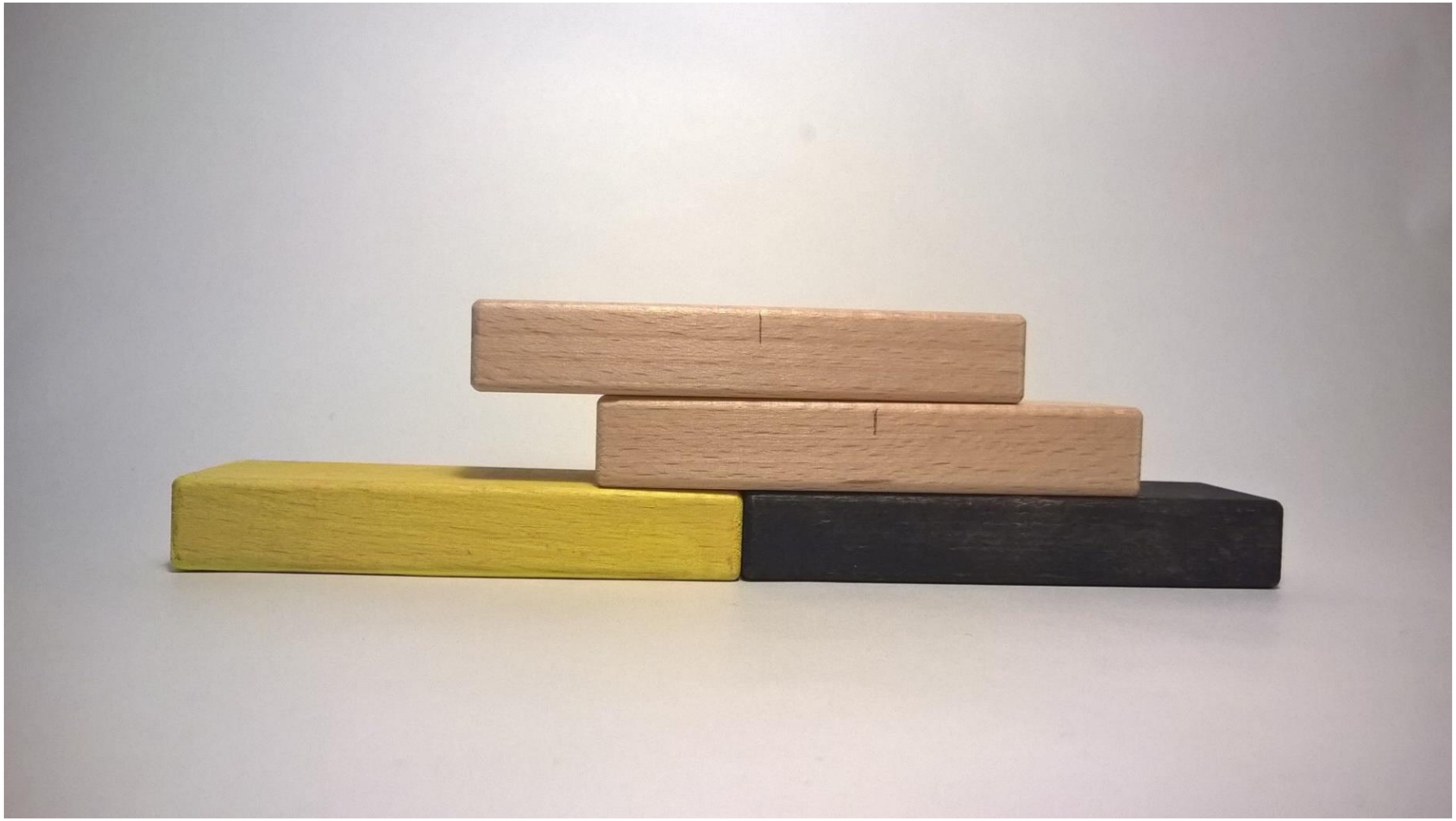

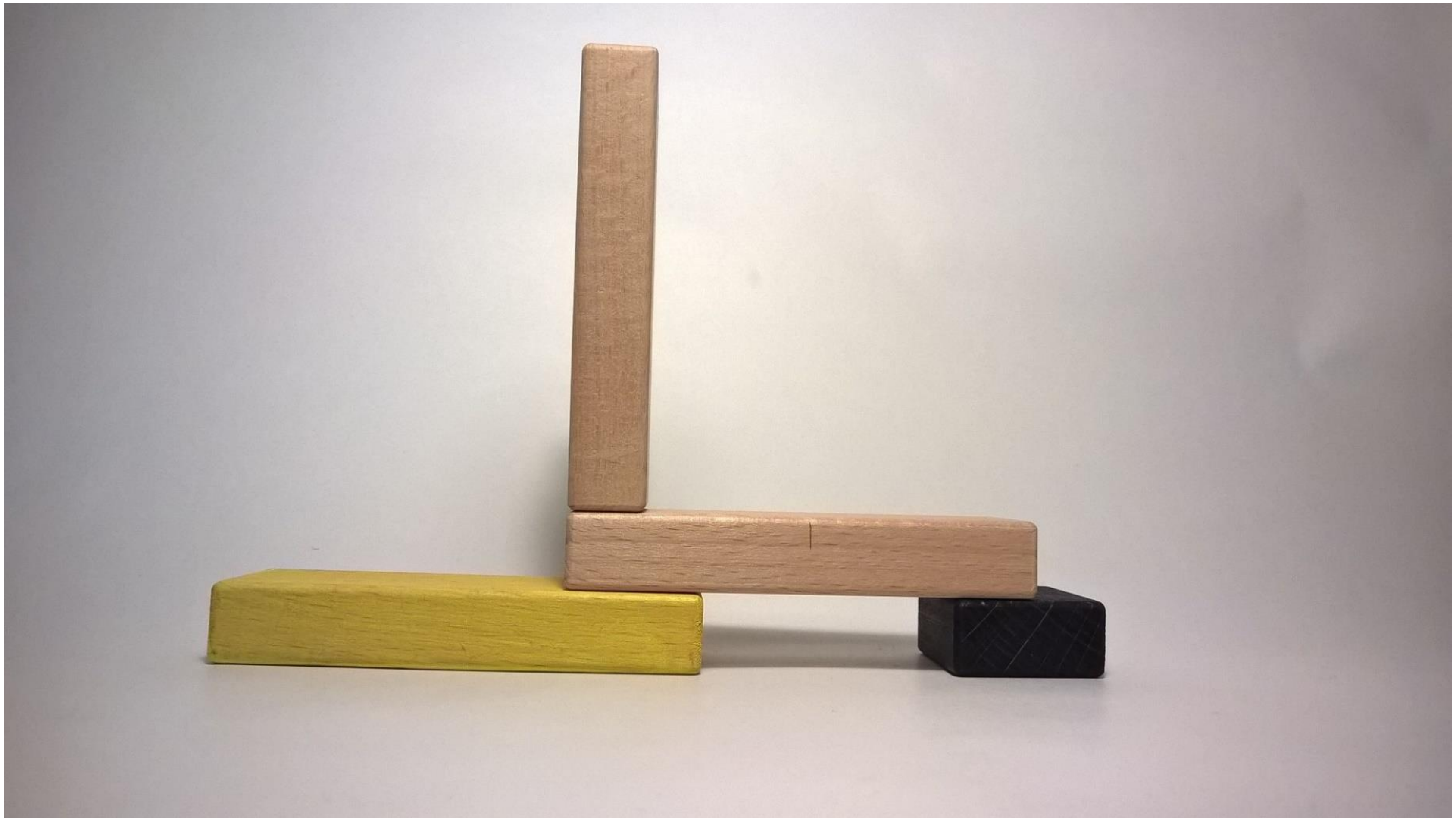

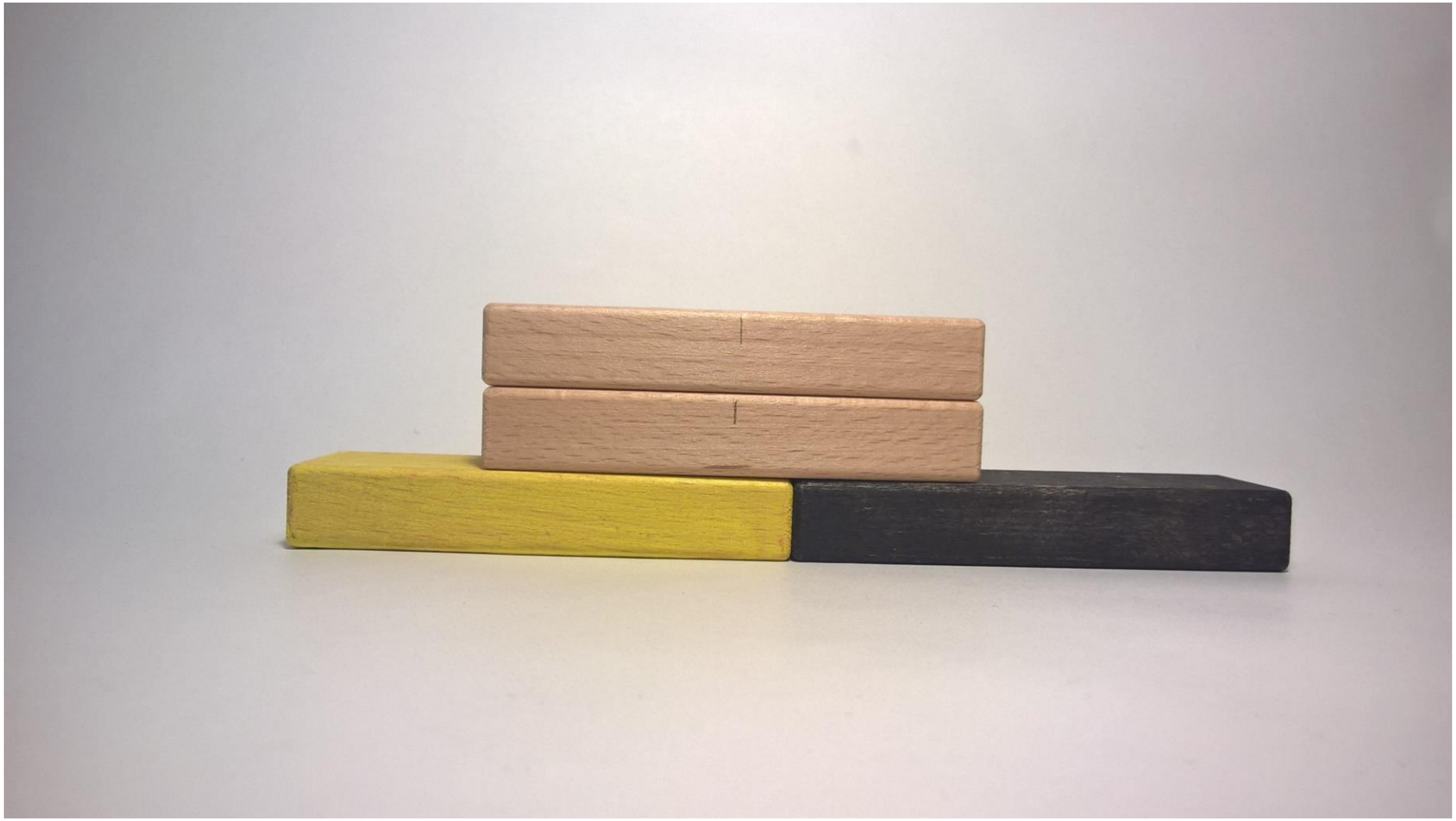

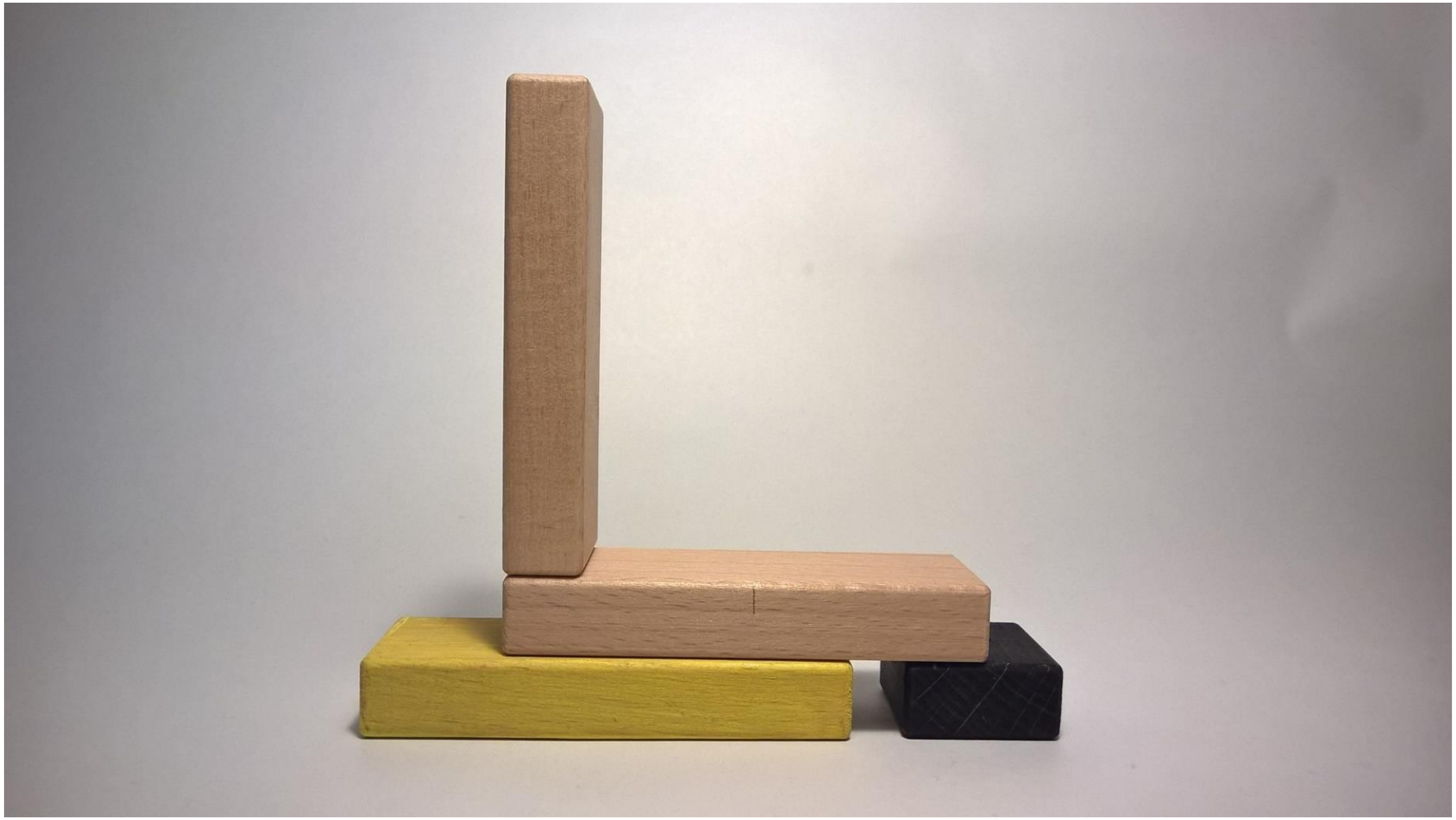

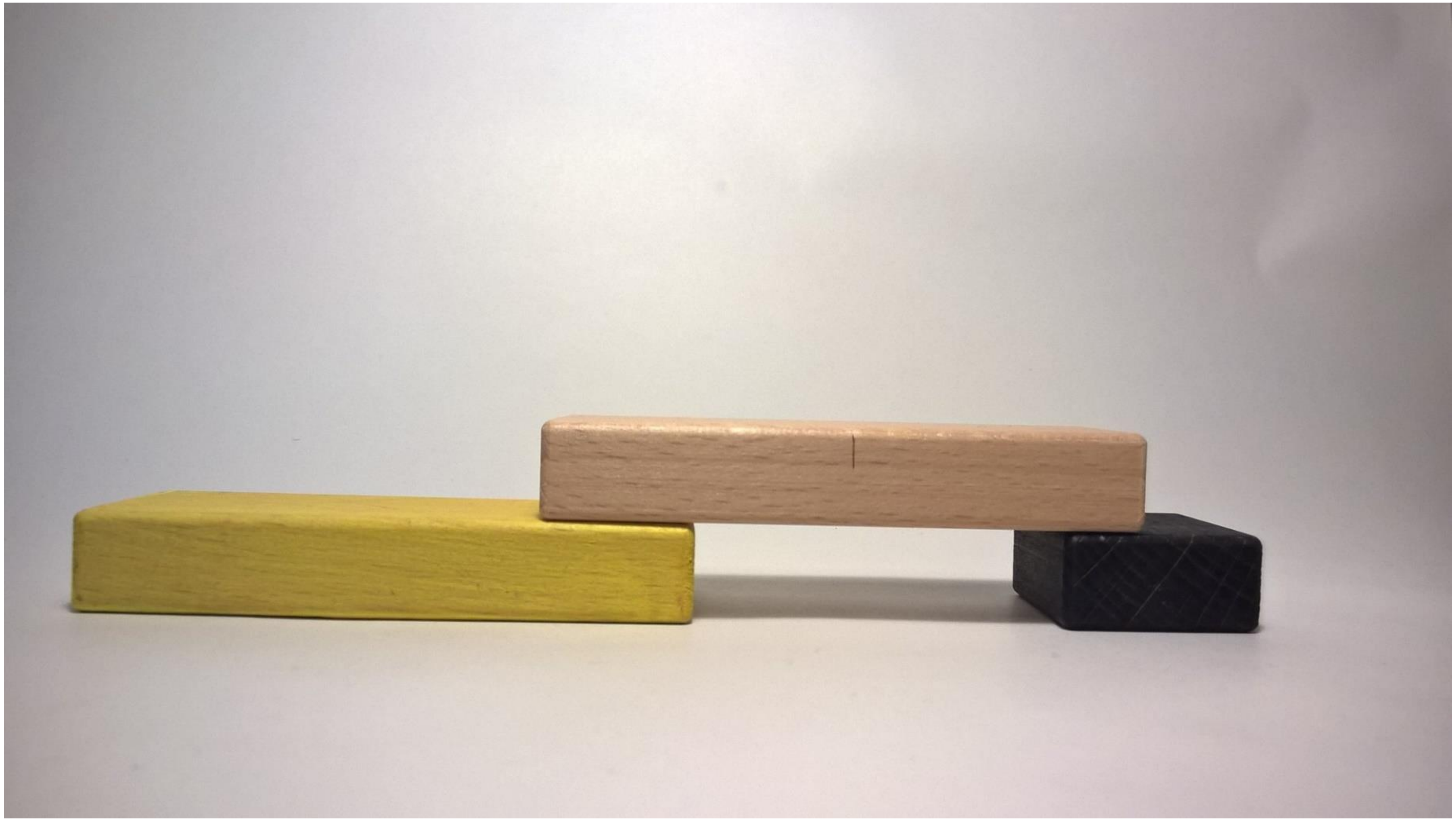

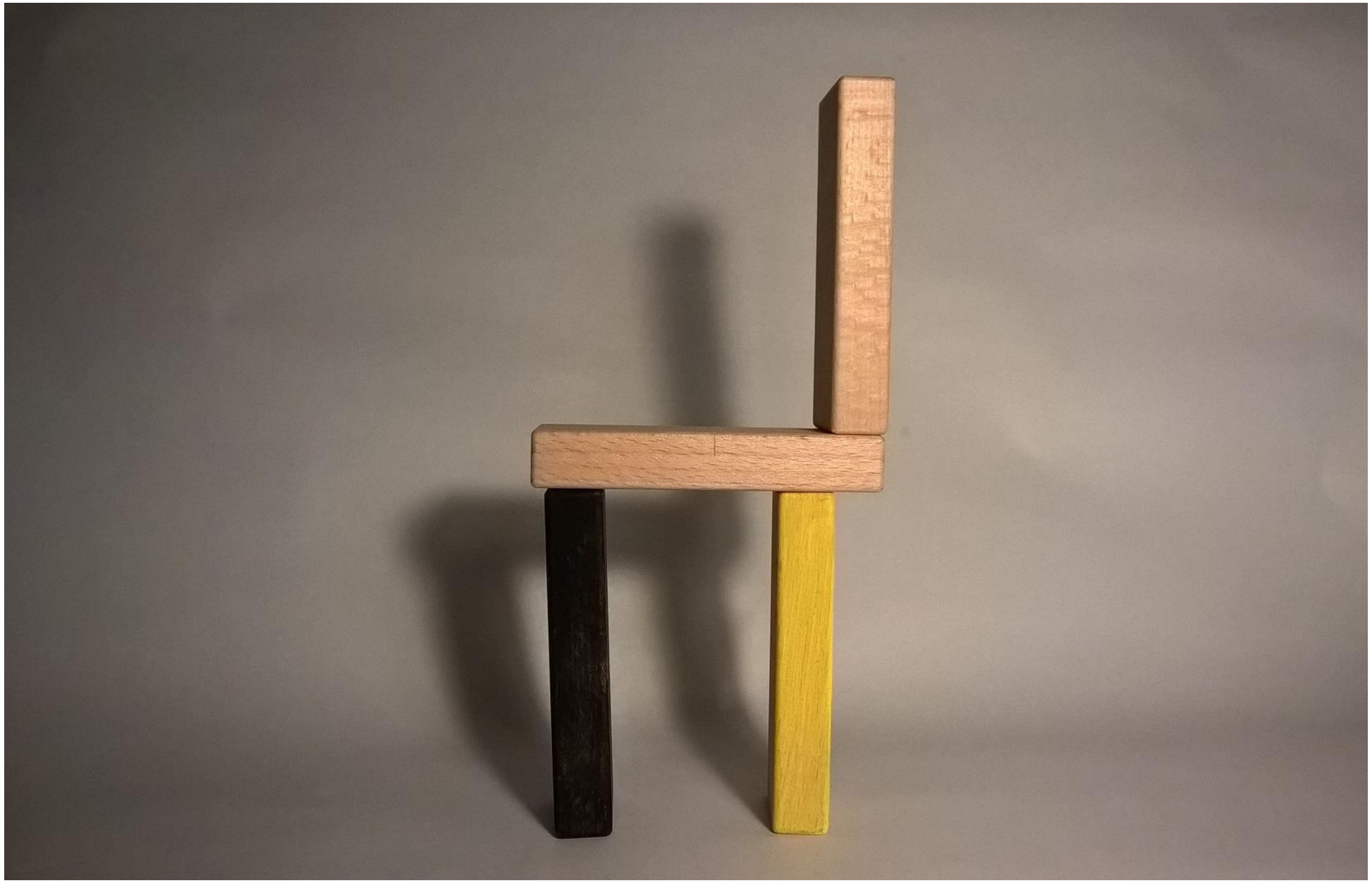

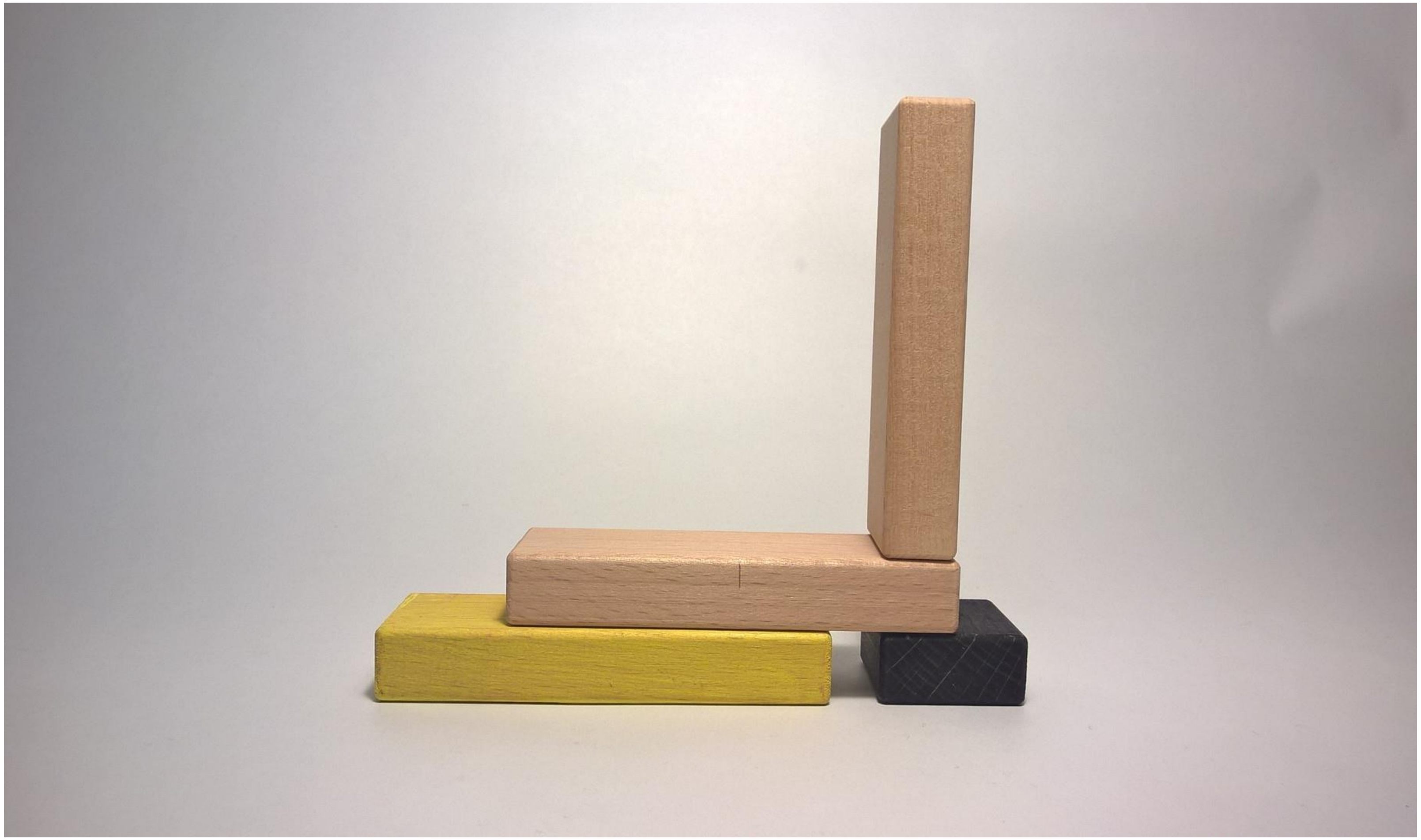

Supplementary Material 5. Items of the transfer test.

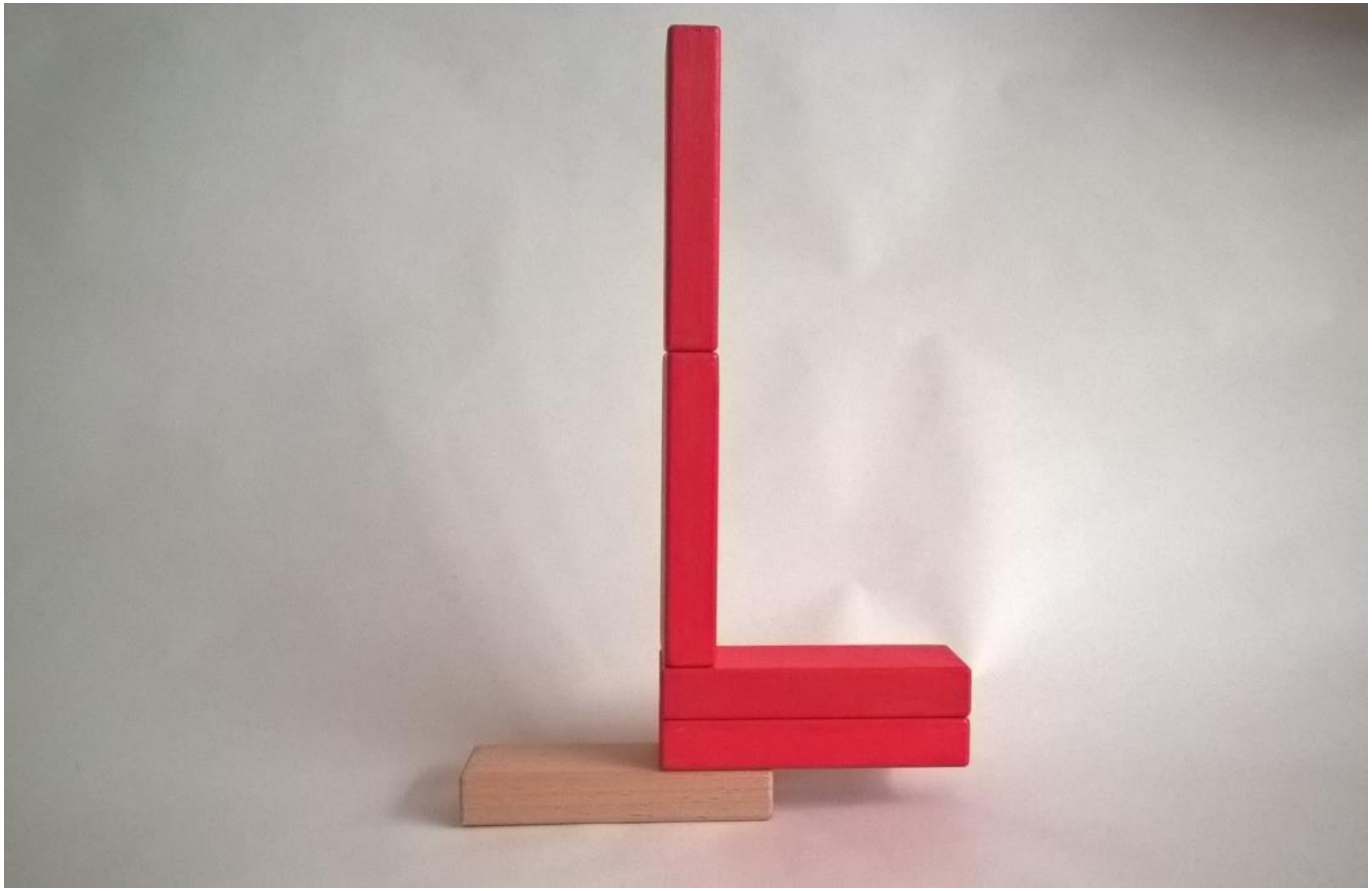

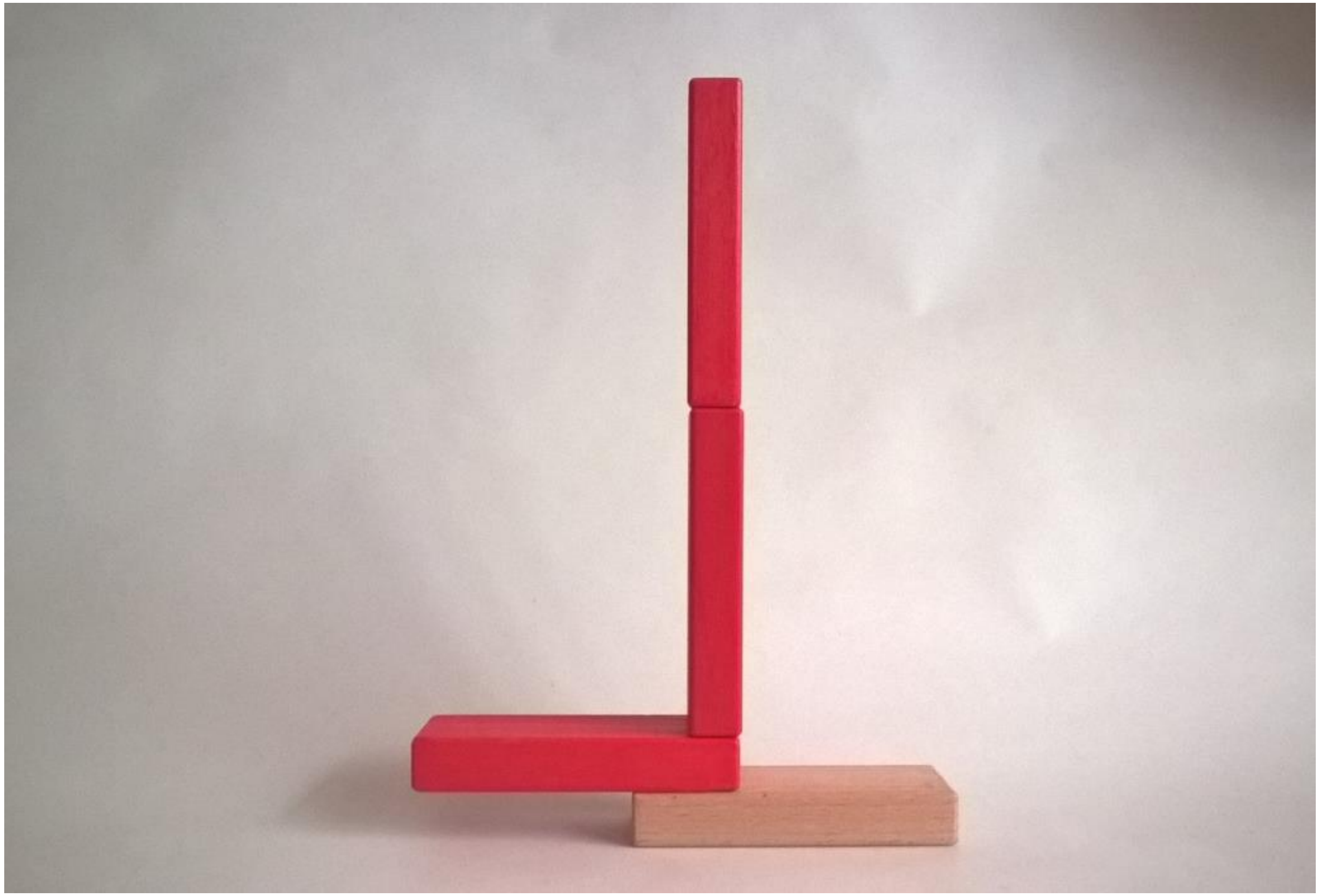

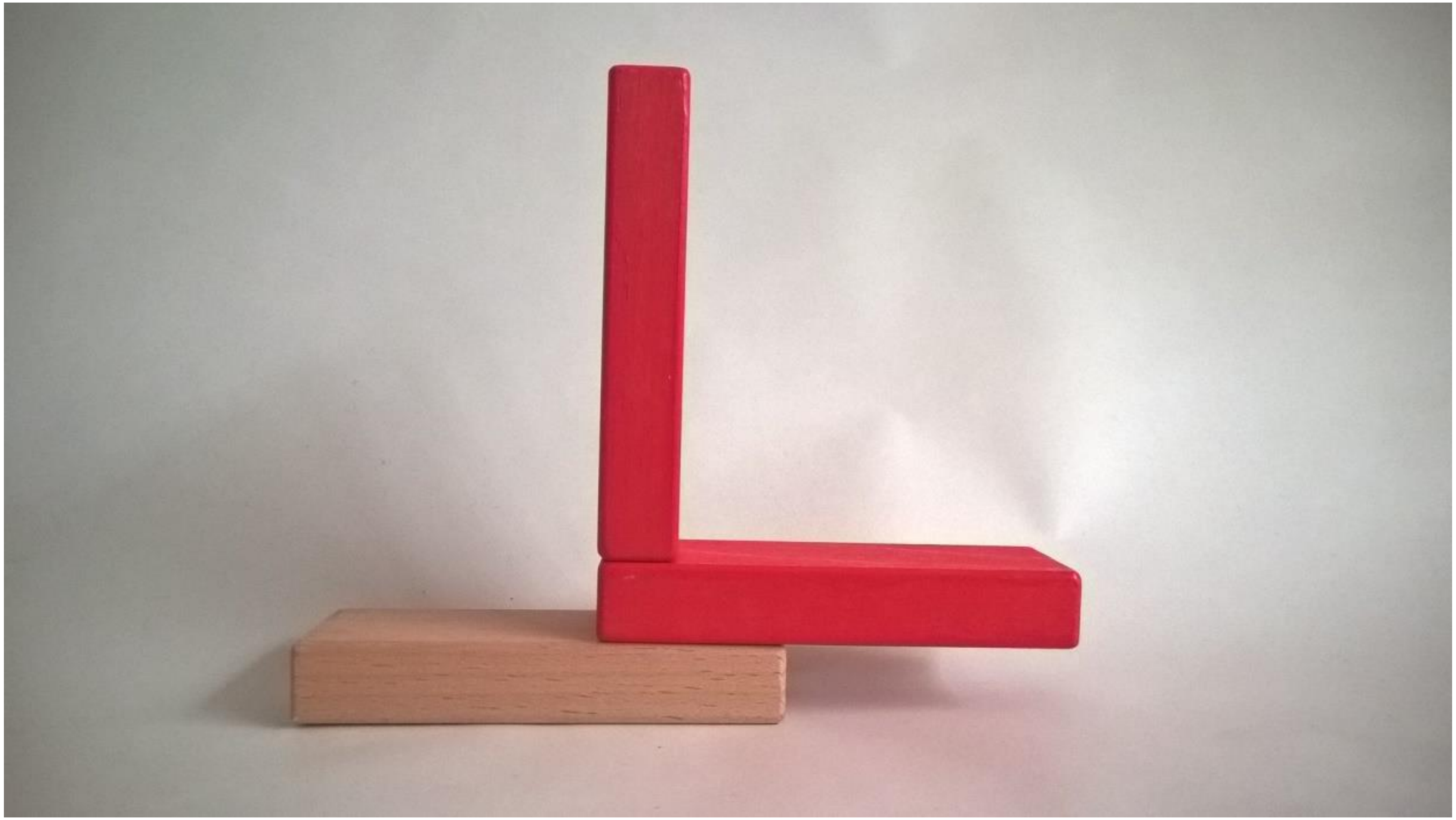

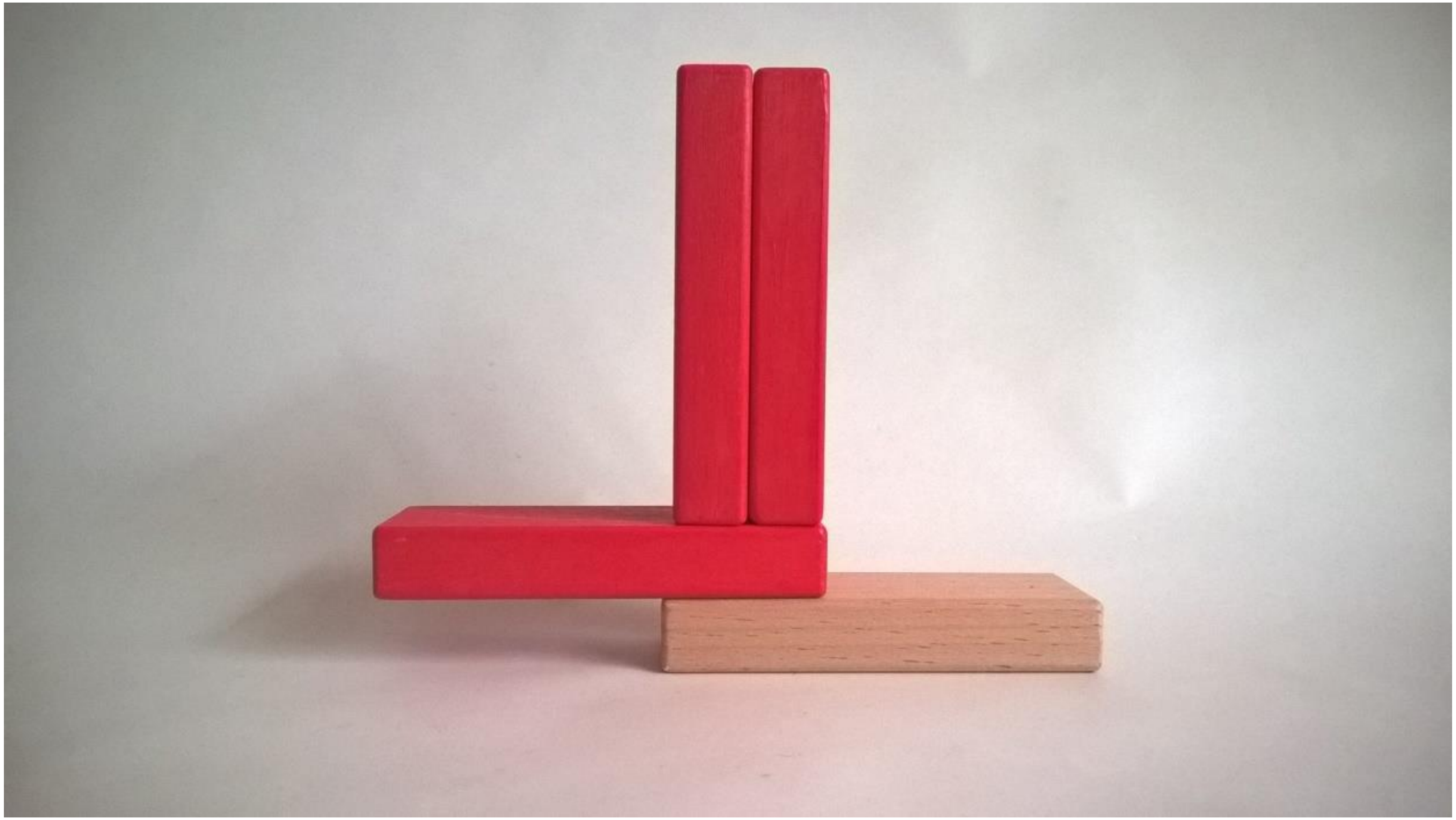

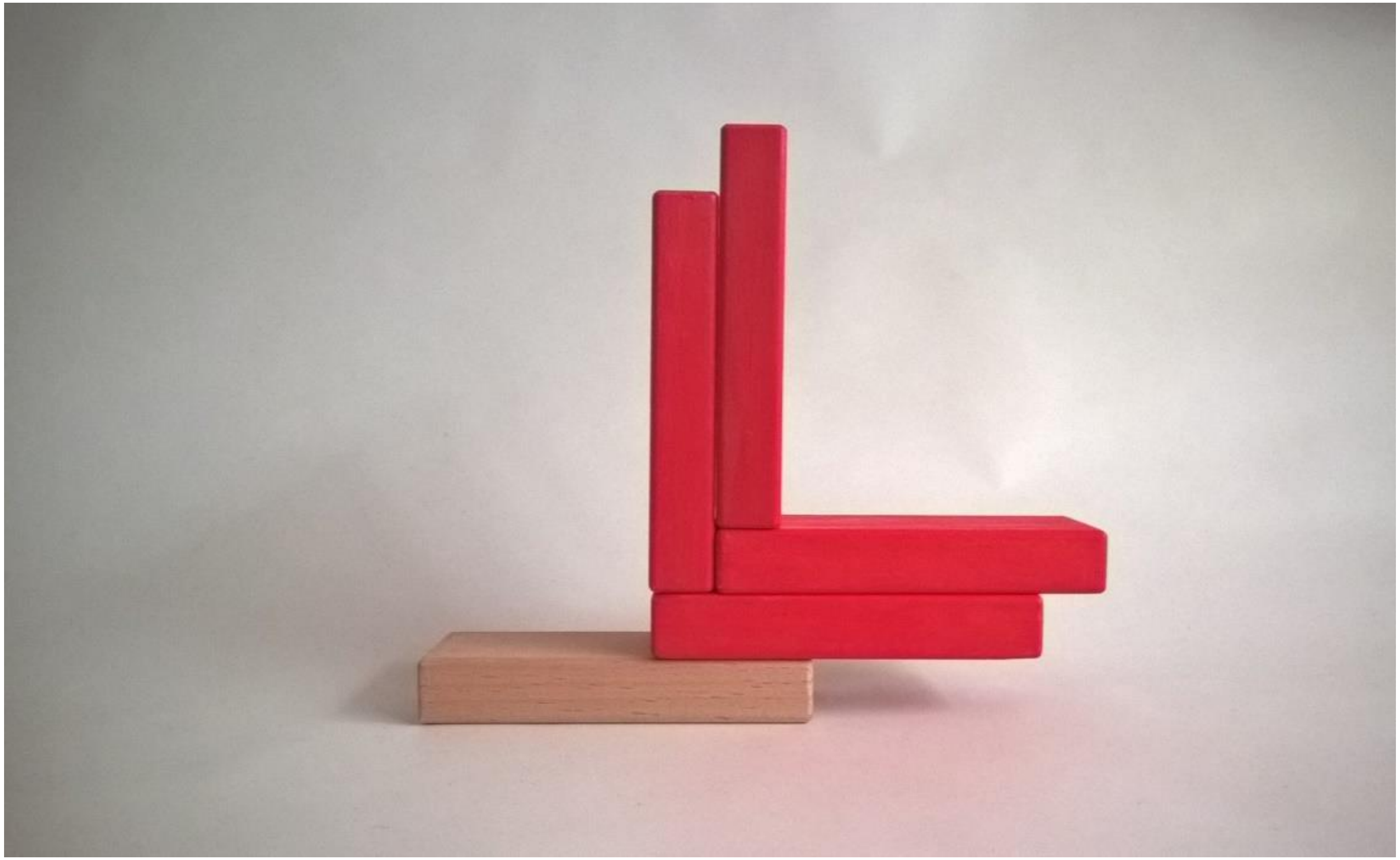

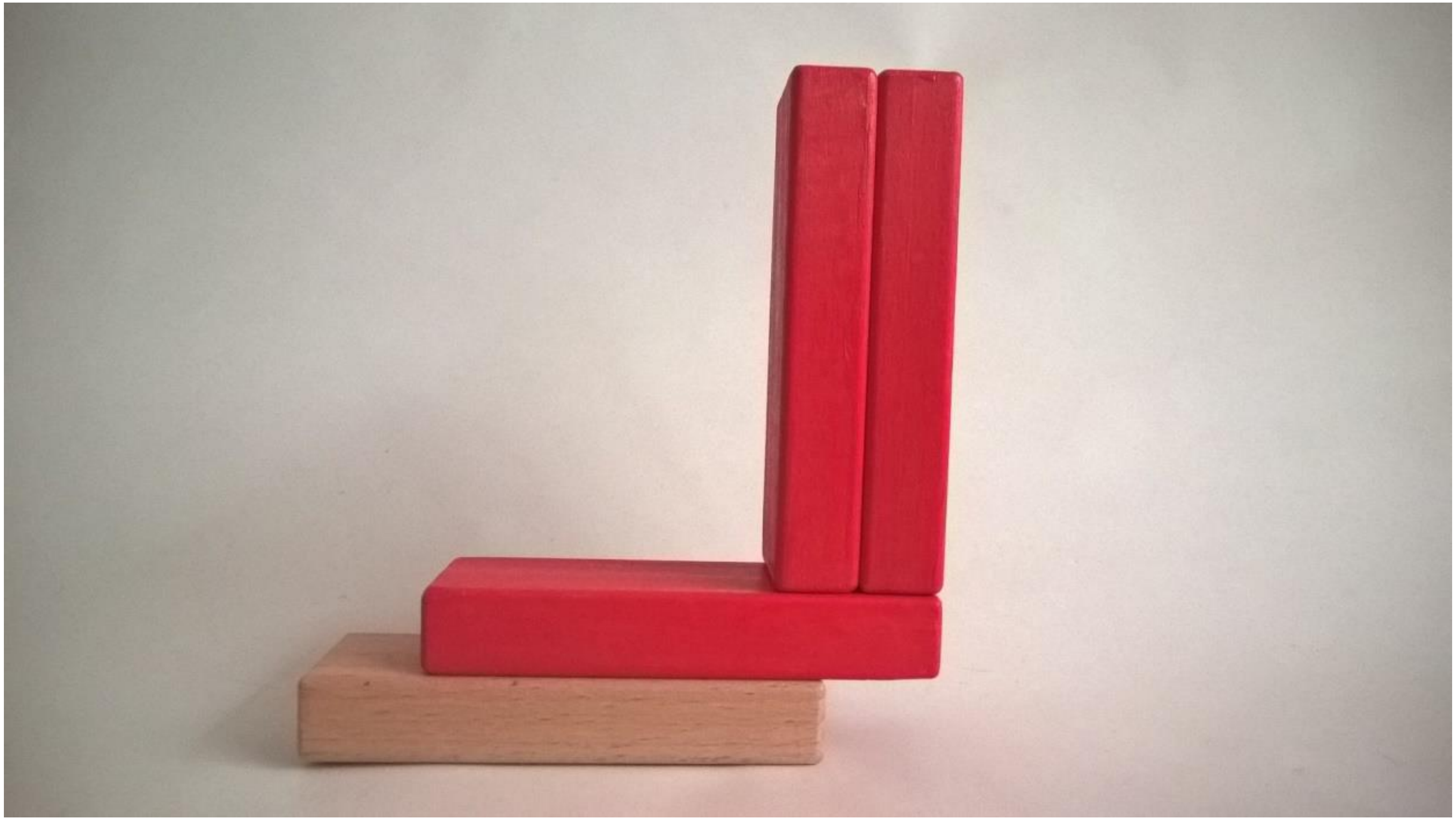

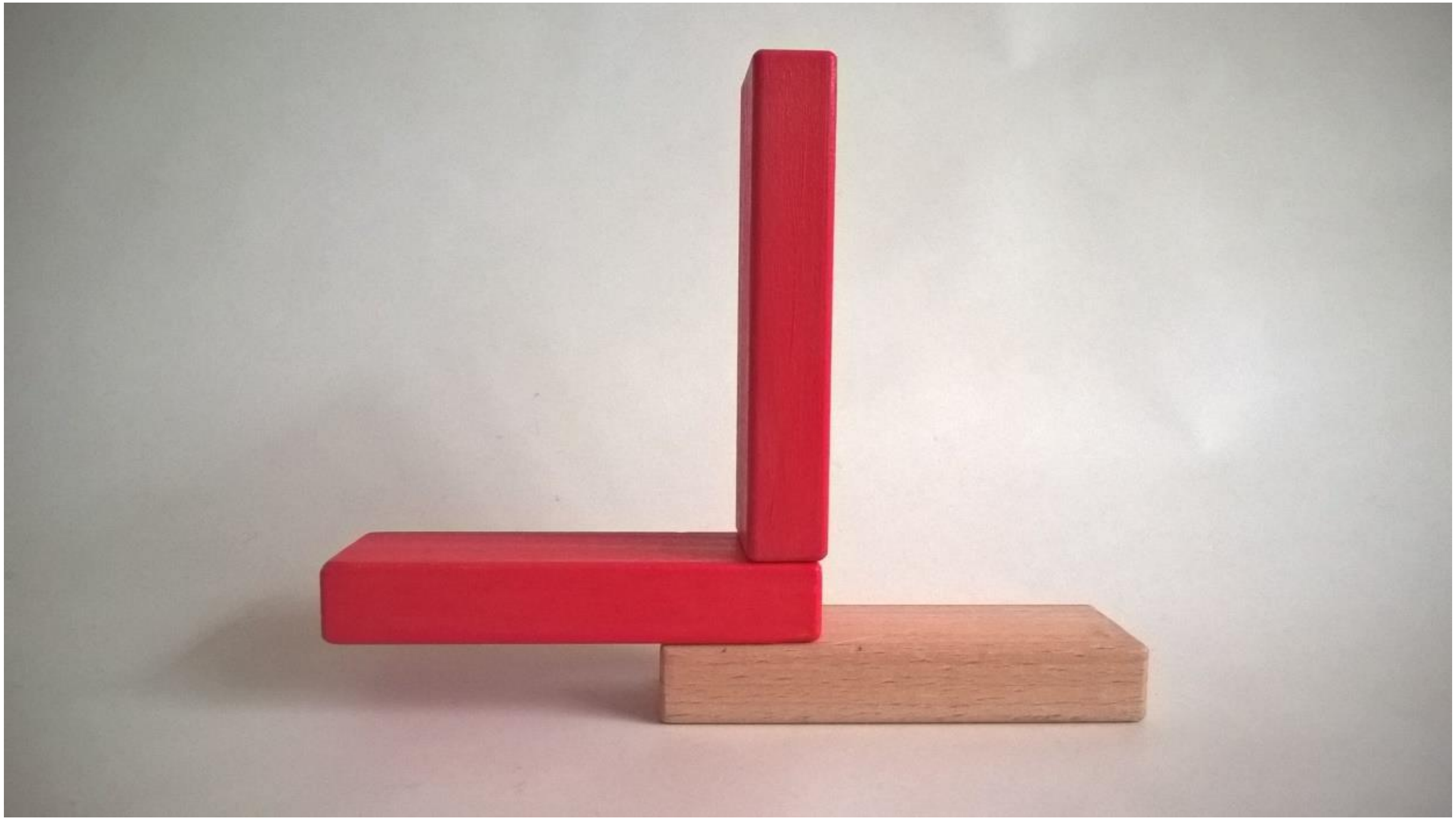

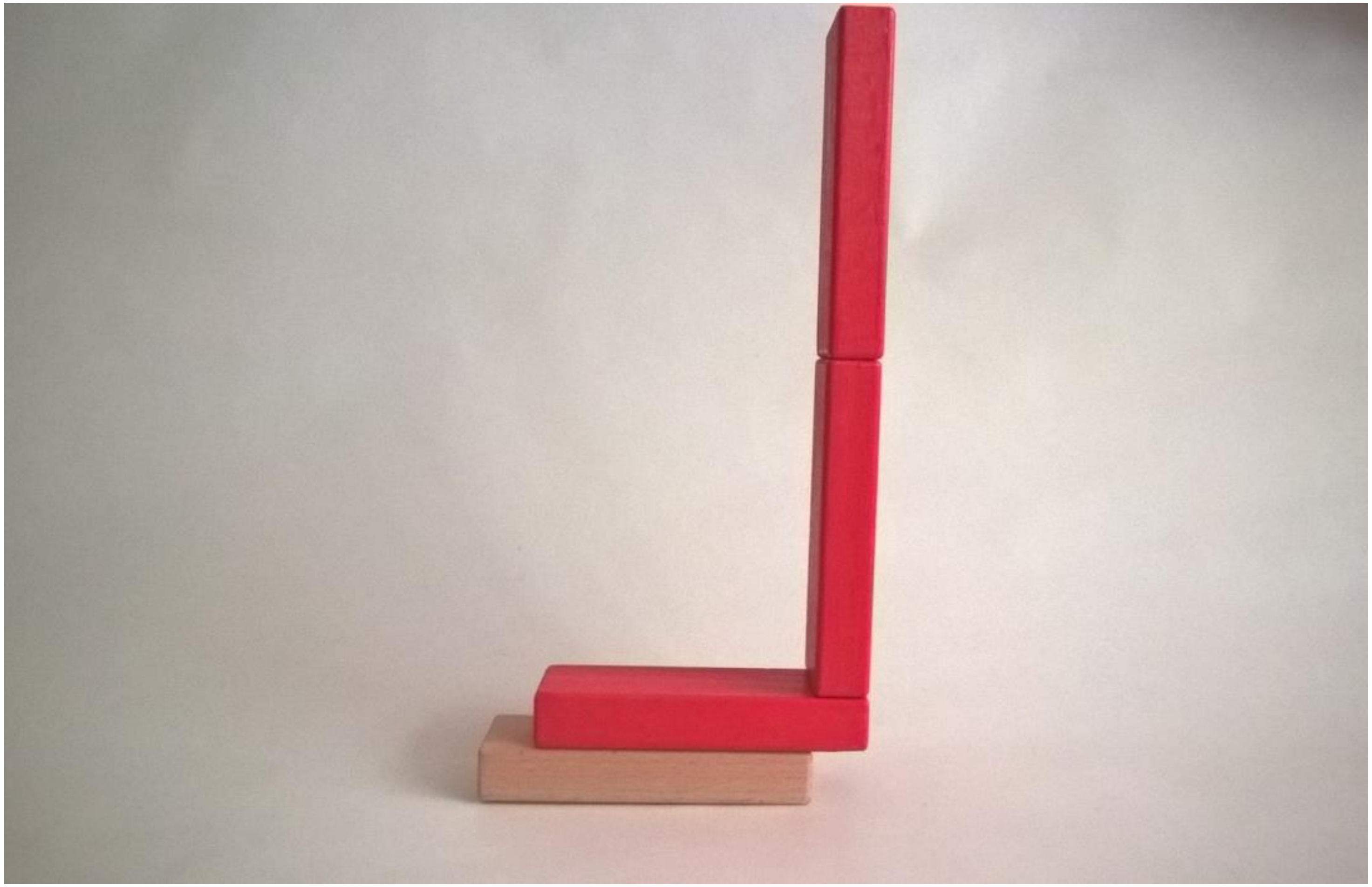

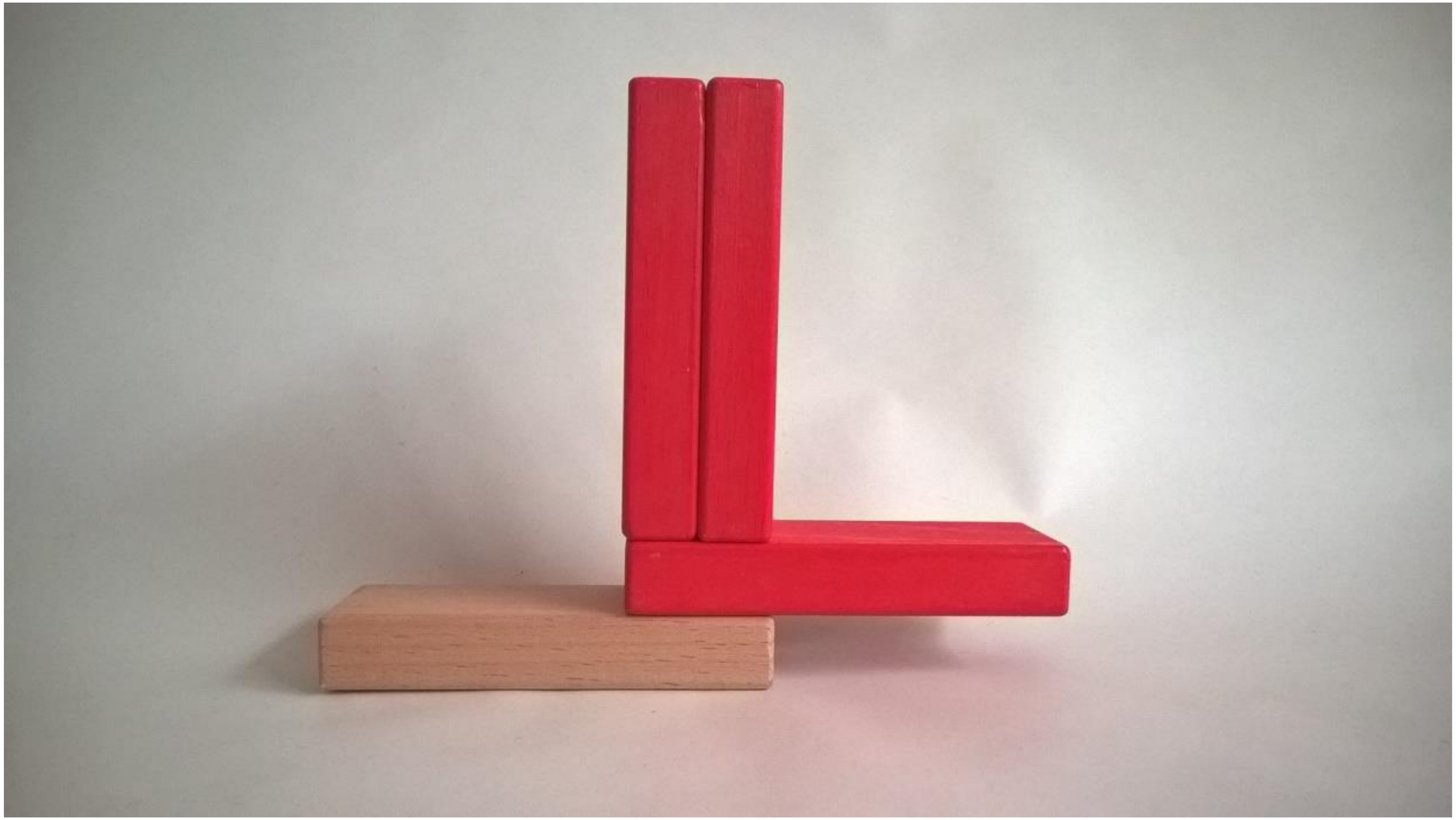

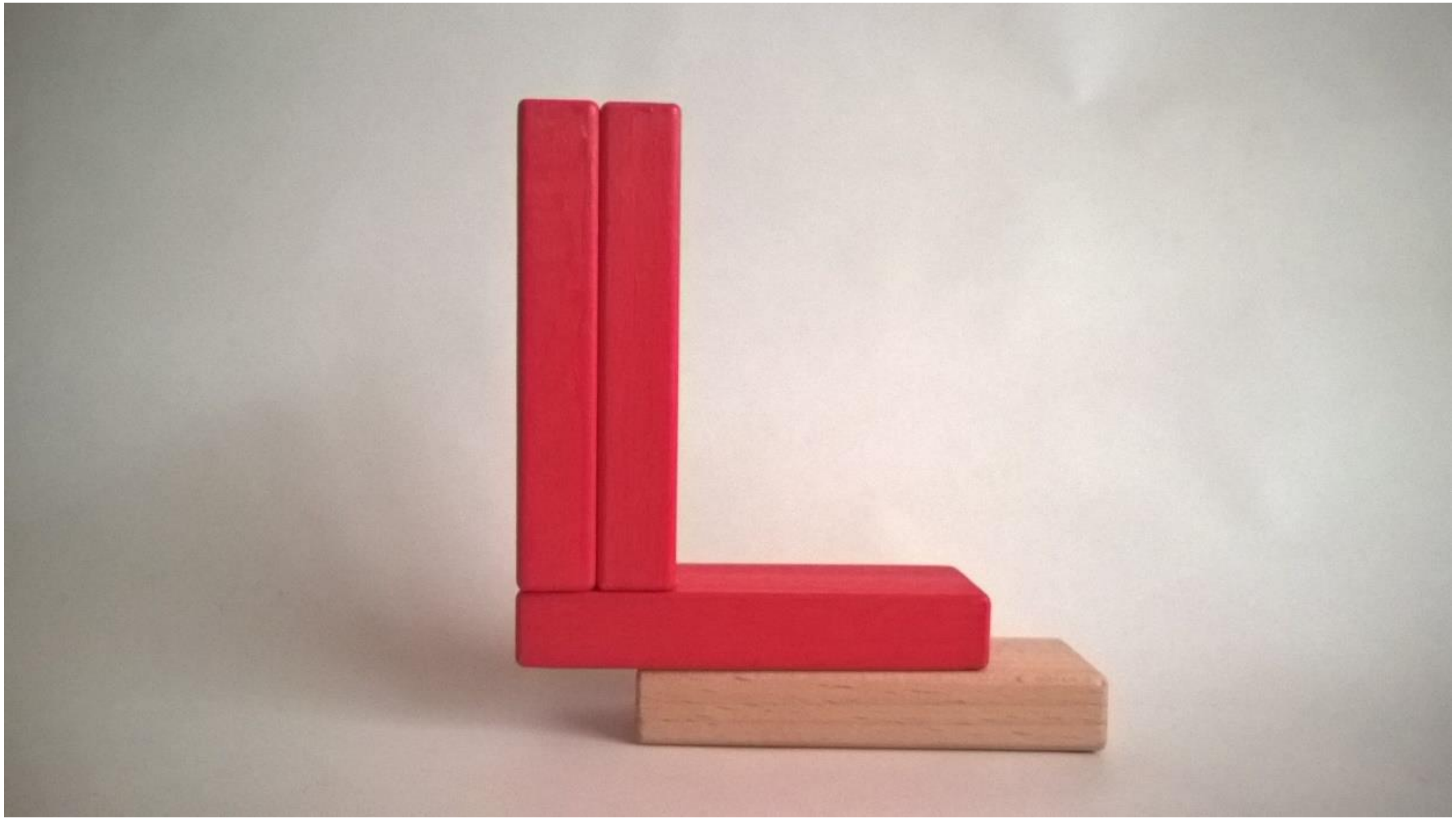

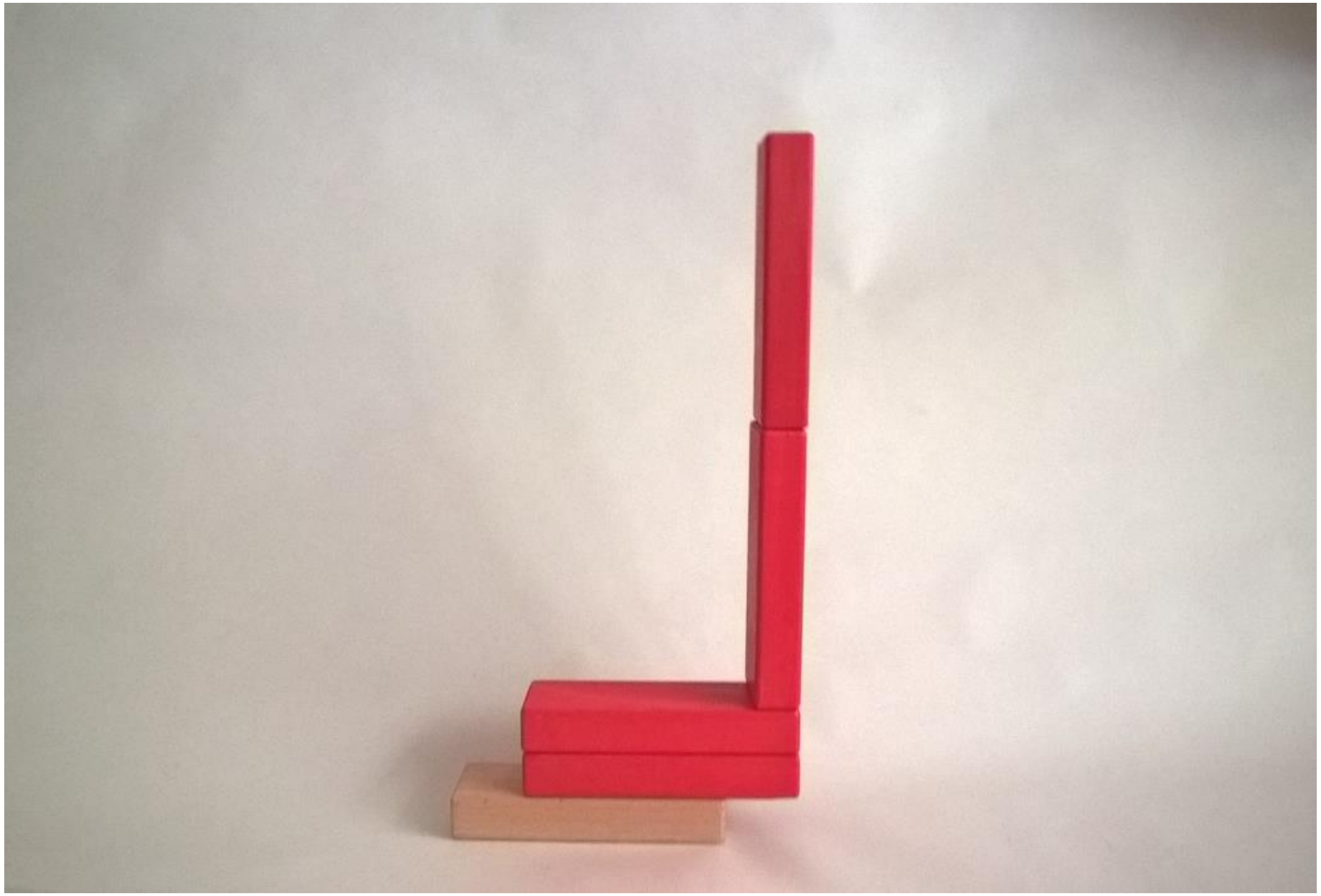

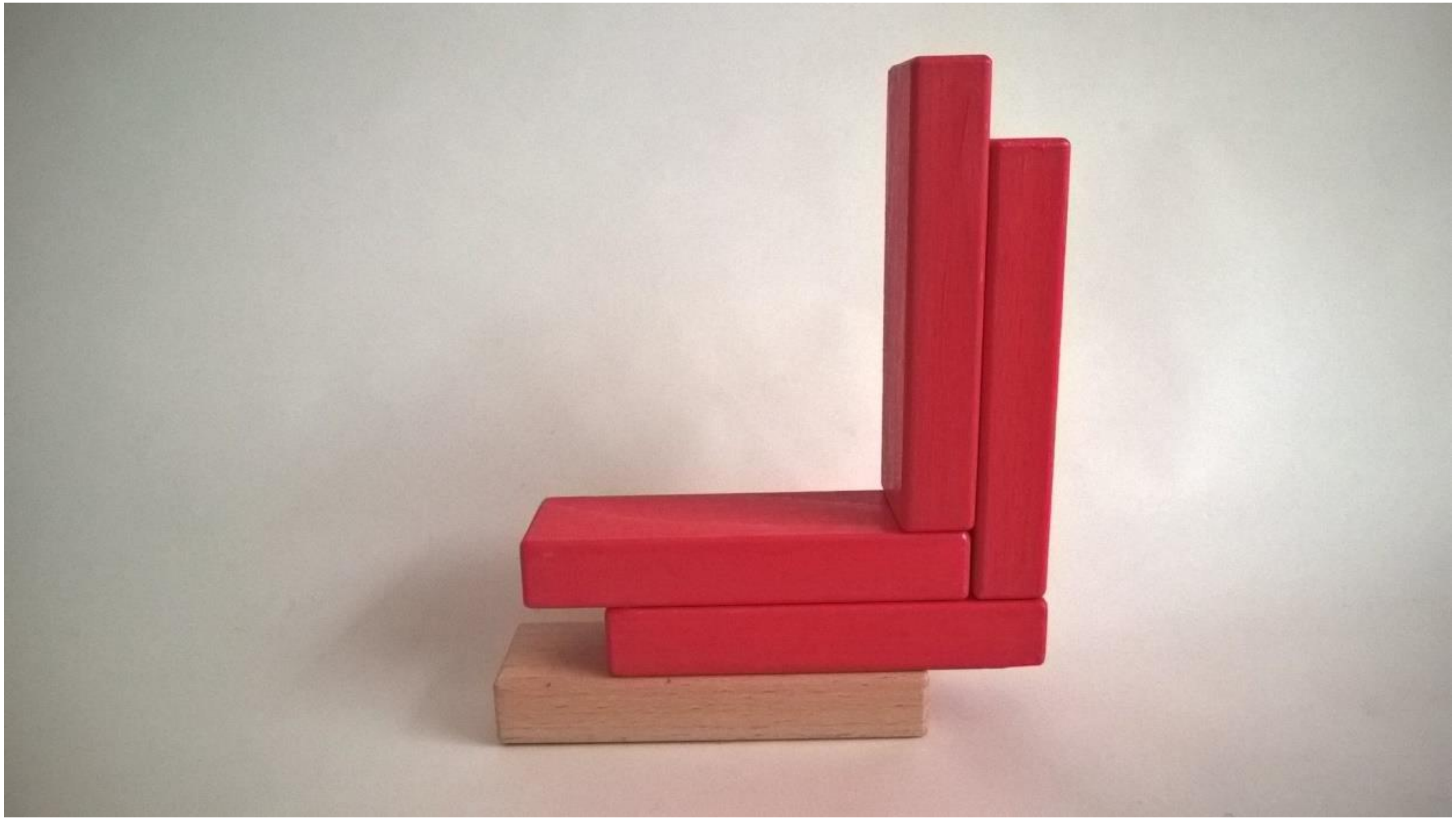

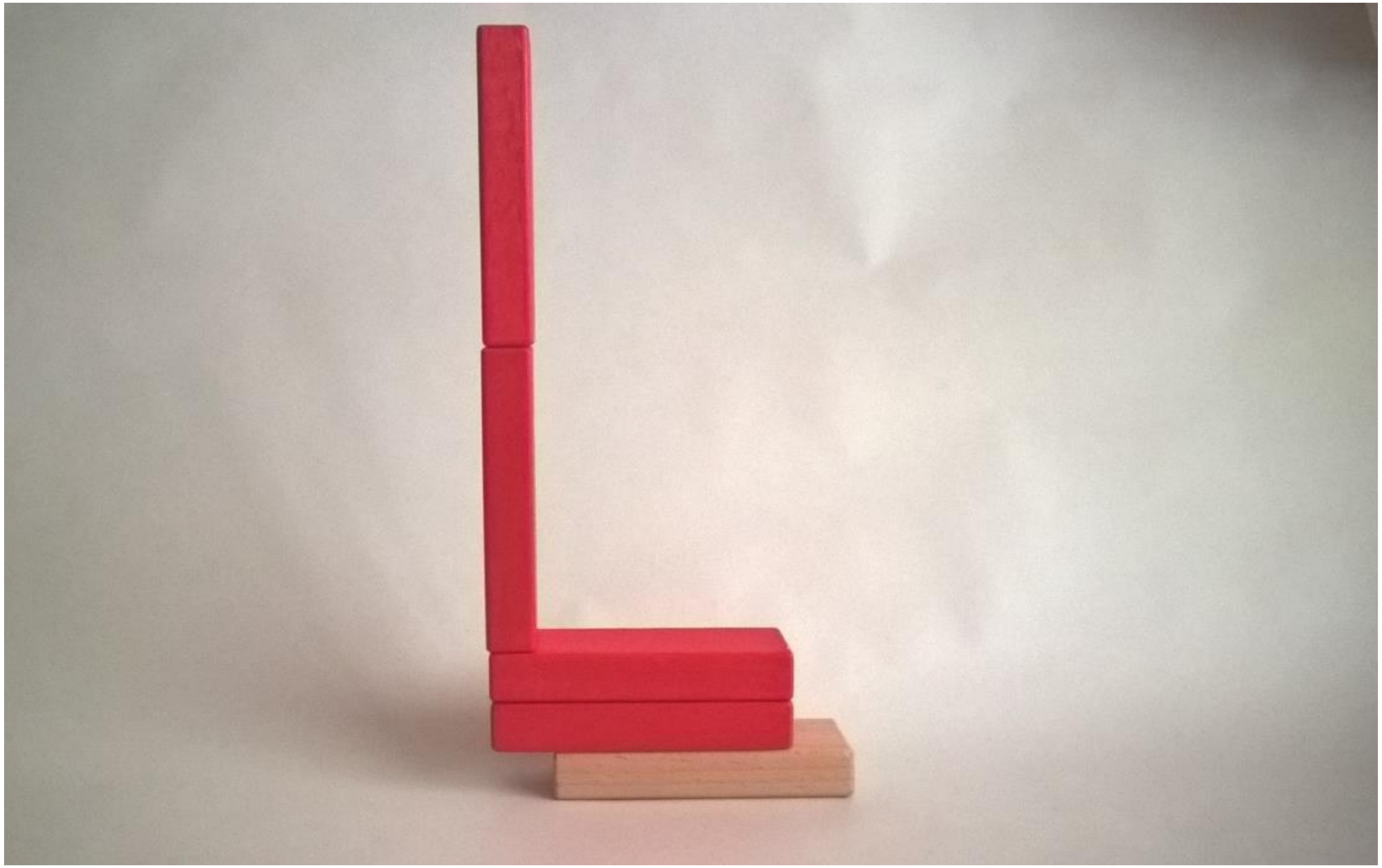

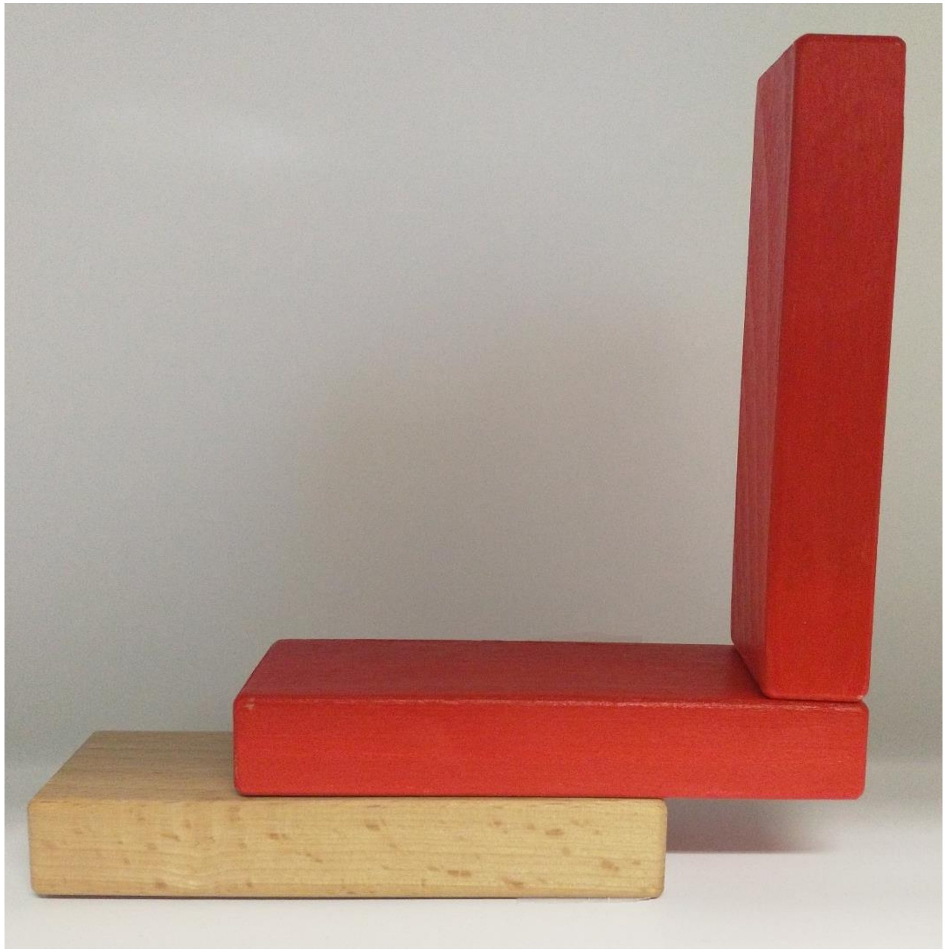

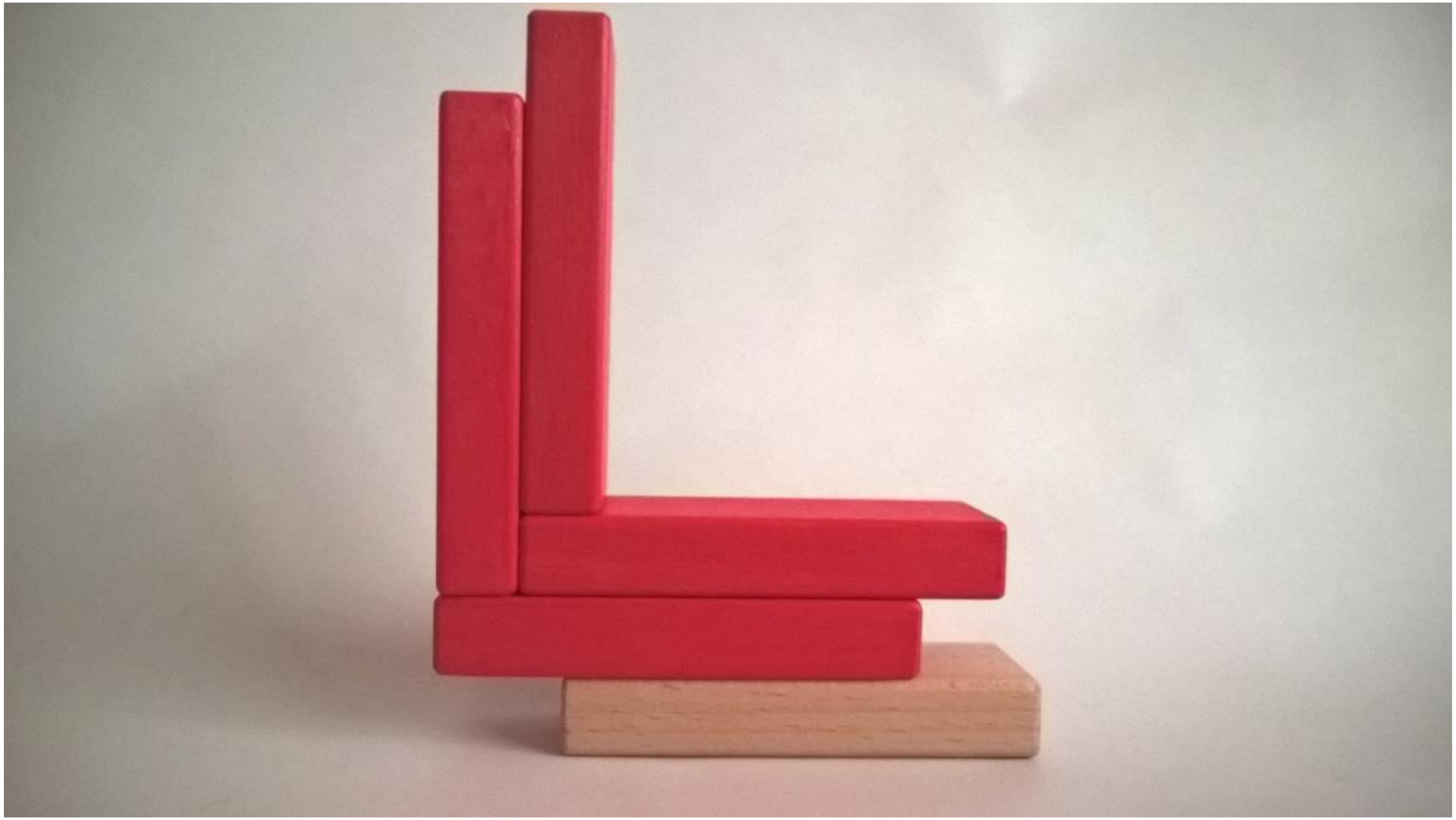

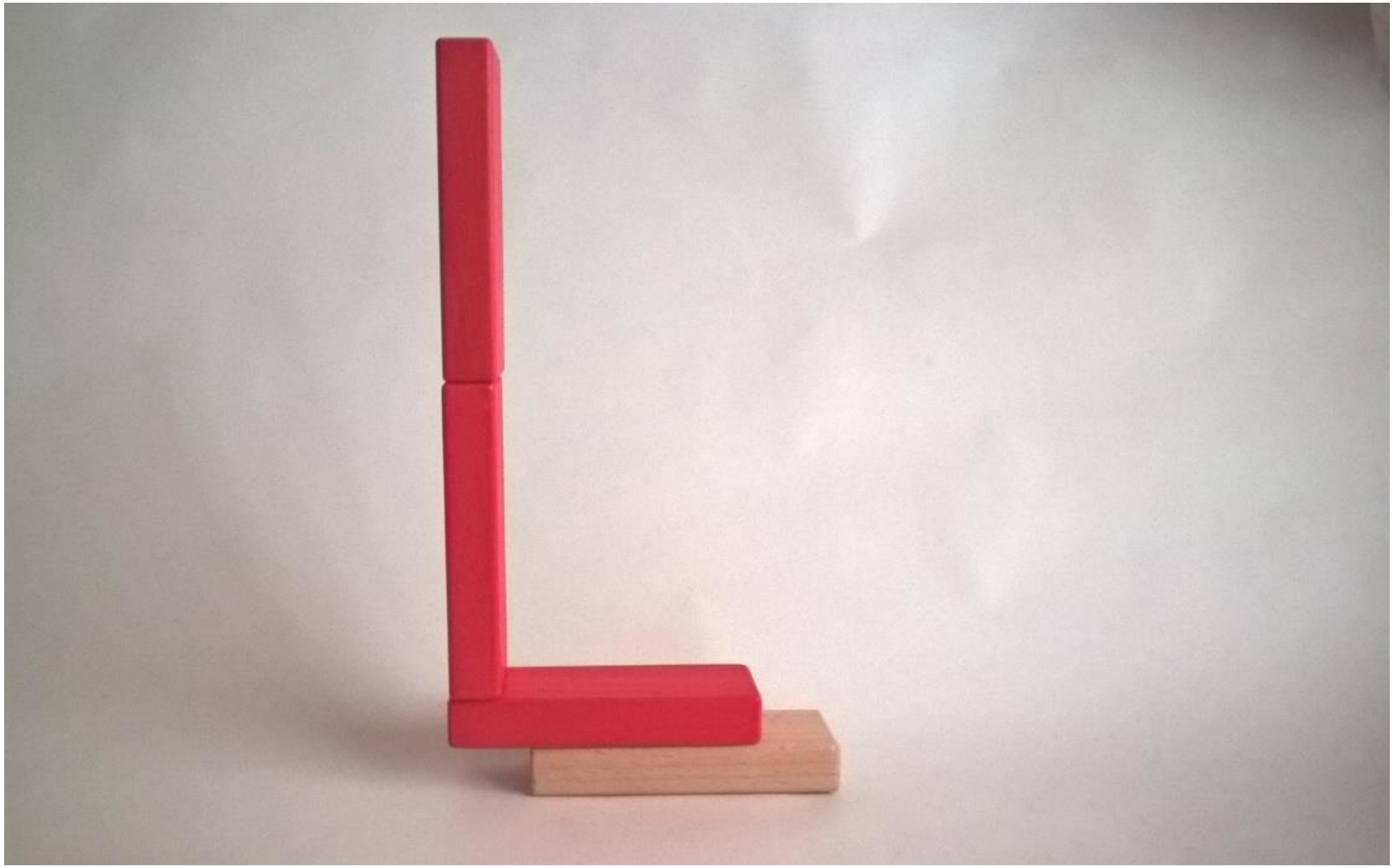

Supplementary Material 6. Percentages of correct answers on the reasoning test.

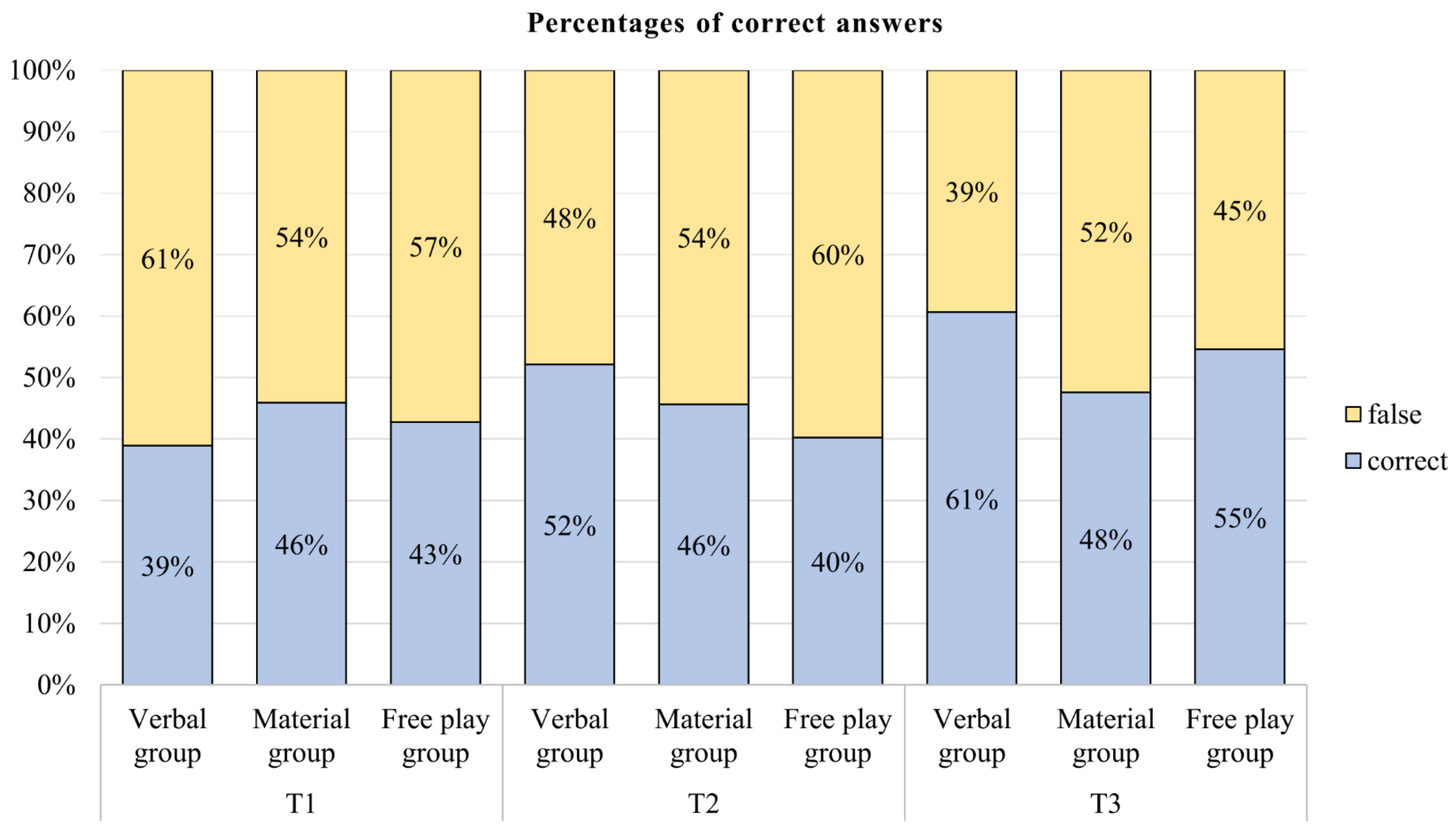

Supplementary Material 7. Results of the Kaplan-Meier analyses.

Table

Kaplan-Meier analysis for children's acquisition of Mass theory at T2

Survival function as estimated by the Kaplan-Meier analysis for children's acquisition of Mass theory at T2																		
	Verbal group						Material group						Free play group					
					95% CI						95% CI						95% CI	
time	n.risk	n.event	surv	SE	LL	UL	n.risk	n.event	surv	SE	LL	UL	n.risk	n.event	surv	SE	LL	UL
3	53	12	.77	.06	.67	.89	47	4	.91	.04	.84	1.00	51	3	.94	.03	.88	1.00
Survival function as estimated by the Kaplan-Meier analysis for children's acquisition of Mass theory at T2 and T3																		
	Verbal group						Material group						Free play group					
					95% CI						95% CI						95% CI	
time	n.risk	n.event	surv	*SE*	LL	UL	n.risk	n.event	surv	*SE*	LL	UL	n.risk	n.event	surv	*SE*	LL	UL
4	47	11	.77	.06	.65	.90	43	2	.95	.03	.89	1.00	47	3	.94	.04	.87	1.00
5	36	4	.68	.07	.56	.83	41	3	.88	.05	.79	.99	44	2	.89	.05	.81	.99
6	32	4	.60	.07	.47	.75	38	5	.77	.06	.65	.91	42	2	.85	.05	.76	.96

Notes. N.risk = Number of children/group. N.event = number of children explaining with Mass consistently. Surv = percentage of children who did not answer consistently. *SE* = standard error. LL = lower level 95% confidence interval. UL = upper level 95% confidence interval.

Table

Kaplan-Meier analysis for children's acquisition of Mass theory over T2 and T3

	Consistent																	
	Verbal group						Material group						Free play group					
					95% CI						95% CI						95% CI	
time	n.risk	n.event	surv	SE	LL	UL	n.risk	n.event	surv	SE	LL	UL	n.risk	n.event	surv	SE	LL	UL
4	16	2	.88	.08	.73	1.00	18	1	.94	.05	.84	1.00	12	1	.92	.08	.77	1.00
5	-	-	-	-	-	-	17	1	.89	.07	.76	1.00	-	-	-	-	-	-
6	-	-	-	-	-	-	16	2	.78	.10	.61	1.00	11	1	.83	.11	.65	1.00
	Inconsistent																	
	Verbal group						Material group						Free play group					
					95% CI						95% CI						95% CI	
time	n.risk	n.event	surv	SE	LL	UL	n.risk	n.event	surv	SE	LL	UL	n.risk	n.event	surv	SE	LL	UL
4	29	8	.72	.08	.58	.91	26	2	.92	.05	.83	1.00	34	2	.94	.04	.87	1.00
5	21	4	.59	.09	.43	.80	24	2	.85	.07	.72	1.00	32	2	.88	.06	.78	1.00
6	17	3	.48	.09	.33	.70	22	4	.69	.09	.54	.90	30	1	.85	.06	.74	.98

Notes. N.risk = Number of children/group. N.event = number of children explaining with Mass consistently. Surv = percentage of children who did not answer consistently. *SE* = standard error. LL = lower level 95% confidence interval. UL = upper level 95% confidence interval.
